# Reliability‐Based Evaluation of the Robustness of Constructed Wetlands for Sustainable Sanitation: Implications for Design and Operation

**DOI:** 10.1002/wer.70572

**Published:** 2026-09-16

**Authors:** Clélio Rodrigo Paiva Rafael, Joan Garcia, Eduardo Lucas Subtil

**Affiliations:** ^1^ Laboratory of Urban Wastewater Treatment and Water Reuse (LabTAUS), Center of Engineering, Modeling and Applied Social Sciences, Federal University of ABC Santo André São Paulo Brazil; ^2^ GEMMA ‐ Group of Environmental Engineering and Microbiology, Department of Civil and Environmental Engineering Universitat Politècnica de Catalunya Barcelona Tech Barcelona Spain

**Keywords:** decentralized sanitation, nature‐based solutions, performance variability, reliability coefficient, wastewater reliability, water reuse

## Abstract

Constructed wetlands (CWs) are low‐cost, low‐energy nature‐based solutions for wastewater treatment and are commonly regarded as robust; however, this perception is often based on removal efficiencies or mean effluent concentrations, without accounting for effluent variability or the probability of maintaining concentrations below regulatory limits. This study applied the reliability coefficient (RC) to 34 vertical‐flow constructed wetland (VFCW), horizontal‐flow constructed wetland (HFCW), and hybrid constructed wetland (HCW) systems treating domestic wastewater identified through a systematic literature review. Removal efficiencies and final effluent concentrations were evaluated against Brazilian discharge and non‐potable reuse benchmarks. Reliability was calculated separately for each system–parameter combination at a 95% target non‐exceedance probability. Grouped comparisons examined only descriptive patterns, dispersion, and overlap among configurations. Sensitivity to alternative target probabilities and analytical applications to design and monitoring were also examined. Mean removal efficiencies ranged from 79.8%–94.5% for biochemical oxygen demand (BOD), 76.9%–87.8% for chemical oxygen demand (COD), 73.6%–90.8% for total suspended solids (TSS), 59.6%–65.9% for total nitrogen (TN), and 68.0%–75.4% for total phosphorus (TP). Under the stringent scenario, mean effluent concentrations were below the selected limits in 21/22 systems for BOD, 33/34 for COD, 22/22 for TSS, 4/25 for TN, and 6/18 for TP. Under the assumed log‐normal model, incorporating effluent variability at 95% reduced probabilistic compatibility to 20/22, 27/34, 21/22, 2/25, and 4/18 systems, respectively. Grouped mean RC values ranged from 0.38 to 0.69, but substantial within‐configuration dispersion and overlap showed that no single RC can represent a flow configuration or treatment train. At target probabilities of 95% or higher, increasing variability consistently reduced the RC; at lower probabilities, the non‐monotonic response of the equation altered system rankings. Incorporating the RC into first‐order HFCW and VFCW models converted regulatory limits into system‐ and parameter‐specific allowable mean effluent concentrations, providing an analytical basis for reliability‐informed design and monitoring, although the framework was not experimentally validated as an independent sizing method. The results support comparatively wide operating margins for organic matter and suspended solids, whereas sustained nutrient compatibility remained less frequent. Removal efficiency, residual concentration, and reliability should therefore be interpreted jointly using RC values specific to each system and parameter.

## Summary

- Constructed wetlands reliably remove BOD, COD, and TSS under varying operational conditions.
- Nutrient removal (TN, TP) remains variable, requiring additional treatment or operational adjustments for strict compliance.
- Reliability coefficient (RC) enables probabilistic assessment of treatment performance and regulatory compliance.
- Incorporating RC into design improves sizing, ensuring performance under variable influent conditions.
- RC supports operational decision‐making by identifying performance variability and guiding targeted system adjustments.

## Graphical abstract

Reliability analysis of 34 constructed wetlands showed wide operating margins for organic matter and suspended solids, but less frequent sustained compliance for nitrogen and phosphorus. Robustness should therefore be assessed using system‐ and parameter‐specific reliability, together with removal efficiency and final effluent concentration.

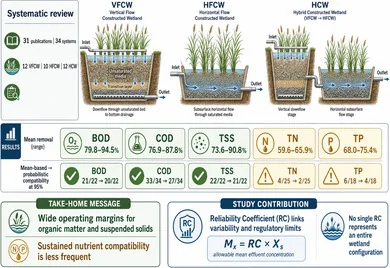

## 1 Introduction

Globally, wastewater management remains a major environmental and public health challenge. The need for sustainable and low‐energy treatment technologies has intensified as developing and emerging economies struggle to expand sanitation coverage while minimizing environmental impacts. Within this context, constructed wetlands (CWs) have gained prominence as nature‐based solutions (NbS) capable of combining cost‐effectiveness, operational simplicity, and strong ecological integration (Prost‐Boucle et al. 2023). They also deliver ecosystem services and socioeconomic benefits consistent with the International Union for Conservation of Nature framework for NbS (International Union for Conservation of Nature (IUCN) 2020), which helps distinguish genuine NbS from more interventionist approaches (Rafael et al. 2025). Unlike broader green infrastructure, CWs are specifically designed and operated to treat wastewater through controlled wetland processes while providing these additional benefits.

These systems reproduce the functions of natural wetlands through physical, chemical, and biological processes, including filtration, sedimentation, and biofilm‐mediated degradation (Gorgoglione and Torretta 2018; Sanchez et al. 2018) and can remove organic matter, nutrients, and pathogens (Vymazal 2022; Xu et al. 2023).

Although CWs are already a consolidated technology, their performance is commonly evaluated using mean removal efficiencies and key hydraulic and loading parameters, such as hydraulic retention time (HRT) and loading rates, whereas temporal variability and the probability of maintaining effluent quality over time are less frequently assessed. The Brazilian design consensus reports expected global removal efficiencies for the treatment system as a whole when horizontal and conventional vertical‐flow CWs are preceded by primary or low‐efficiency secondary treatment. Under adequate design, construction, and operation, the expected efficiencies are > 85% for biochemical oxygen demand (BOD), > 80% for Chemical Oxygen Demand (COD), and > 85% for Total Suspended Solids (TSS), whereas Total Nitrogen (TN) and Total Phosphorus (TP) removal generally remain < 50% and < 20%, respectively (von Sperling and Sezerino 2018). These values represent expected performance ranges rather than global averages or medians. CW performance is influenced by system configuration, climatic conditions, influent characteristics, hydraulic conditions, and design and operational criteria (von Sperling and Sezerino 2018). Beyond pollutant removal, CWs provide environmental and social co‐benefits, including water quality improvement, biomass valorization for handicrafts (Damon 2024; Chuma Basimine et al. 2024), ornamental plant production (Zurita et al. 2009), and the enhancement of green public spaces (Alikhani et al. 2021). These attributes reinforce their role as decentralized, adaptable, and climate‐resilient wastewater treatment systems (Wei et al. 2025).

In Latin America, Brazil accounts for approximately 37% of regional CW publications (Rodriguez‐Dominguez et al. 2020), although only 52.2% of the sewage generated in the country is treated (Sistema Nacional de Informações sobre Saneamento (SNIS), n.d.). Achieving universal access by 2033, as established by the New Legal Framework for Basic Sanitation (Brasil 2020), requires the expansion of low‐cost and reliable treatment systems. However, the widespread adoption of CWs has been constrained by the historical absence of technical standards, limited professional training, and inadequate operational practices (Vymazal 2022; Rodriguez‐Dominguez et al. 2020). The Brazilian Association of Technical Standards (ABNT) recently issued ABNT NBR 17076:2024 (Associação Brasileira de Normas Técnicas (ABNT) 2024), which provides specific guidelines for design, operation, and maintenance and supports broader implementation. Although several studies have reported mean efficiencies for CWs, reliability, that is, the probability that effluent concentrations remain within legal or reuse standards, has received less attention.

Reliability analysis has long been used for conventional wastewater treatment systems to quantify performance variability and the risk of noncompliance (Śliz and Bugajski 2022). Among the available methods, the probabilistic analytical model recommended by the U.S. Environmental Protection Agency (USEPA) employs the reliability coefficient (RC) to establish compliance thresholds and estimate future performance, supporting operational and regulatory strategies (Oliveira and von Sperling 2008; Padalkar and Kumar 2018; Sojitra et al. 2023).

The RC model has been applied to multiple treatment technologies, such as waste stabilization ponds, activated sludge, physicochemical processes, disinfection, sequencing batch reactors, membrane bioreactors, and extended‐aeration systems (Padalkar and Kumar 2018; Alves et al. 2021; Carvalho et al. 2022; Hamza et al. 2022; Owusu‐Ansah et al. 2015). In Brazil, RC‐based assessments have been mostly limited to conventional systems (Alves et al. 2021; Alderson et al. 2015). However, comparative applications of a common RC framework to vertical‐, horizontal‐, and hybrid‐flow CWs under different levels of discharge restriction remain limited (Lombard‐Latune et al. 2020).

In the analytical formulation adopted in this study, the RC is determined by the coefficient of variation (CV) of the effluent concentration and the selected target non‐exceedance probability. At the 95% non‐exceedance probability adopted in the primary analysis, higher CV values result in lower RC values and, consequently, a lower allowable mean effluent concentration. The sensitivity of the RC to other target probabilities is evaluated in the Materials and Methods section.

Against this background, this study presents a systematic comparative application of the RC to vertical‐flow constructed wetlands (VFCWs), horizontal‐flow constructed wetlands (HFCWs), and hybrid constructed wetlands (HCWs) under Brazilian regulatory conditions. It provides an integrated assessment of CW efficiency and reliability using regulatory performance bands based on federal, state, and reuse standards. In this study, reliability refers to the probability that an effluent concentration remains below a selected regulatory limit, whereas robustness refers more broadly to the capacity of a system to maintain acceptable performance under variable conditions. The RC is therefore interpreted as a probabilistic measure of reliability relative to a selected concentration limit, not as a direct measure of removal efficiency or temporal performance stability. By quantifying reliability under variable conditions, the study provides empirical evidence for assessing the robustness commonly attributed to CWs. It also examines the incorporation of the RC into CW design models. The regulatory findings are specific to the Brazilian benchmarks adopted in this study, whereas the analytical procedure may be adapted to other regulatory contexts using locally applicable limits and system‐specific variability data.

Considering these aspects, this study aims to evaluate the efficiency and reliability of vertical‐, horizontal‐, and hybrid‐flow CWs in relation to Brazilian discharge and reuse standards and corresponding state regulations. Data were obtained through a systematic review of published studies. Removal efficiencies and RC values were calculated and compared among system configurations and regulatory criteria. Additionally, an analytical RC‐based design framework for CWs is proposed. The findings may support reliability‐informed design and operation of CWs under variable conditions and contribute to the definition of technically achievable discharge targets.

## 2 Materials and Methods

This study comprised a systematic literature review and a system‐level reliability assessment of CWs treating domestic wastewater. Three configurations were evaluated: VFCWs, HFCWs, and HCWs combining vertical‐ and horizontal‐flow units. The evaluated water‐quality parameters were BOD, COD, TSS, TN, and TP.

The analytical framework comprised four components: system identification and characterization; evaluation of removal performance and compatibility of the reported final effluent concentrations with Brazilian discharge and reuse benchmarks; calculation of system‐ and parameter‐specific reliability indicators; and exploratory assessment of the incorporation of the RC into established CW design equations and operational monitoring. Reliability was calculated separately for each system and water‐quality parameter. Grouped comparisons by flow configuration were used only to examine tendencies, dispersion, and overlap within the reviewed dataset.

Before analysis, each system was characterized using the information reported in the source publication. The extracted descriptors included country and climatic setting, experimental scale, wastewater type, pretreatment, treatment level, flow configuration and treatment‐stage sequence, filter media, vegetation, aeration or ventilation strategy, wetland surface area, HRT, hydraulic loading rate (HLR), and monitoring or operating period. These variables were compiled to document inter‐system heterogeneity and support interpretation of the results. They were not used as adjustment covariates because their reporting was incomplete and inconsistent among studies, and missing descriptors were not imputed. The available characteristics are reported in Tables S1 and S2, whereas influent concentrations are presented in Table S3.

### 2.1 Data Collection and Dataset Construction

A systematic review was conducted in the Scopus database between April and May 2024, without restriction on publication year. Titles, abstracts, and keywords were searched using three configuration‐specific strategies, one each for VFCWs, HFCWs, and HCWs. The searches combined terms related to domestic wastewater and the evaluated performance parameters (BOD, COD, TSS, TN, and TP) with terms identifying vertical‐, horizontal‐, or hybrid‐flow CWs.

Studies were excluded when they were not openly accessible, were written in languages other than Portuguese or English, reported fewer than three of the five evaluated parameters, did not provide a standard deviation (SD), CV, or other temporal information from which dispersion could be obtained, or evaluated non‐domestic wastewater. After application of the eligibility criteria, no Brazilian case study retrieved from the Scopus search remained in the final dataset. The main reasons were incomplete reporting of the summary statistics required for RC calculation, particularly mean effluent concentrations accompanied by SD, CV, or equivalent temporal dispersion information, or reporting of fewer than three of the five target parameters. Therefore, complementary Brazilian studies meeting the same eligibility criteria were identified through Scientific Electronic Library Online (SciELO) and ScienceDirect databases.

**FIGURE 1:**
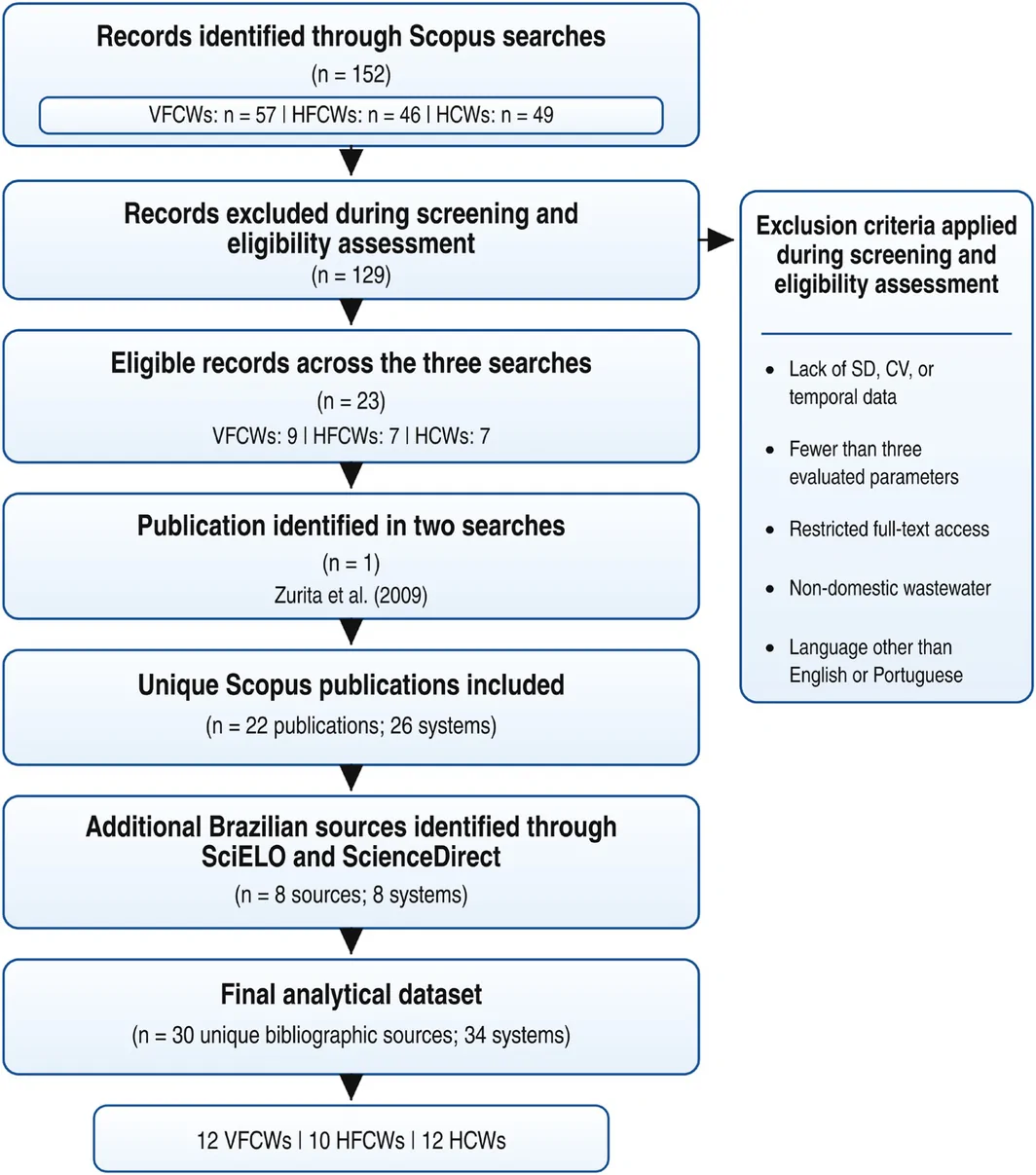
Flow diagram of study identification, screening, eligibility assessment, and inclusion.

The three Scopus searches identified 152 records: 57 related to vertical‐flow systems, 46 to horizontal‐flow systems, and 49 to hybrid systems. After screening, eligibility assessment, and reconciliation of the included sources, 22 unique Scopus publications reporting 26 CW systems were retained. Eight additional Brazilian sources identified through SciELO and ScienceDirect contributed eight systems. The final analytical dataset therefore comprised 30 unique bibliographic sources and 34 CW systems, including 12 VFCWs, 10 HFCWs, and 12 HCWs (Figure 1). The complete traceability of the systems retained in the analytical dataset, including the source study, the exact wetland configuration, the information extracted, and the DOI or access link, is provided in Table S24.

For each eligible system and available parameter, the mean influent concentration, mean final effluent concentration, SD, CV, and removal efficiency were extracted or calculated. A hybrid constructed wetland was defined as a treatment train containing sequential vertical‐ and horizontal‐flow CW units. Twenty‐eight systems treated real domestic or predominantly domestic urban wastewater, whereas six systems used synthetic wastewater formulated to represent domestic sewage. The synthetic subset comprised five VFCWs and one HFCW; all HCWs treated real wastewater.

The individual CW system, rather than the publication, sampling event, or laboratory measurement, was adopted as the primary unit of analysis. A publication was allowed to contribute more than one system only when physically or operationally distinct treatment units or treatment trains were reported with separate performance statistics. Repeated measurements from the same treatment unit were not treated as independent observations. One system‐level value was retained for each available system–parameter combination before any grouped comparison was performed.

The number of systems differed among parameters because the source studies did not consistently report all five variables. No additional minimum group‐size threshold was imposed after application of the eligibility criteria. All available system‐level values were retained, but analyses involving small or unbalanced groups were interpreted cautiously (Table 1).

**TABLE 1:** Number of constructed wetland systems contributing system‐level data for each water‐quality parameter and flow configuration.

Parameter	VFCW	HFCW	HCW
BOD	6	8	8
COD	12	10	12
TSS	6	7	9
TN	10	5	10
TP	7	5	6

*Note:* Values represent individual CW systems reporting the summary statistics required for the corresponding parameter.

### 2.2 Removal Performance, Regulatory Benchmarks, and Exploratory Grouped Comparisons

Removal efficiency was extracted from the source publication when reported or calculated from the system‐level mean influent and effluent concentrations as follows:

(1) $$\eta =\frac{C_{i}-C_{e}}{C_{i}}\times 100$$

where η is removal efficiency (%), Cᵢ is the mean influent concentration, and C_e_ is the mean final effluent concentration. Removal efficiency was interpreted jointly with influent and final effluent concentrations because proportional attenuation does not, by itself, establish compatibility with a concentration‐based threshold.

The mean final effluent concentrations were compared with Brazilian state discharge standards classified according to the restriction bands proposed by Morais and Santos (Morais and dos Santos 2019), the federal criteria established by Brazilian National Environment Council (CONAMA) Resolution No. 430/2011 (Brasil 2011), and the non‐potable reuse criteria established by Joint Resolution No. 1/2017 of the São Paulo State Departments of Health, Environment, and Sanitation and Water Resources (SES/SMA/SSRH) (São Paulo (Estado) 2017). The state discharge standards were classified as very restrictive, restrictive, or less restrictive according to the concentration ranges identified by Morais and Santos (Morais and dos Santos 2019) (Table 2).

**TABLE 2:** State and federal standards for sewage discharge and state criteria for non‐potable wastewater reuse.

Parameter (unit)	Discharge standards				Reuse standards	
	State legislation (1)			CONAMA 430/2011	SES/SMA/SSRH No. 1/2017	
	Very restrictive	Restrictive	Less restrictive		Moderate restriction (2)	Severe restriction (3)
COD (mg O_2_ L^−1^)	< 120 AM, PE	120 ≤ C ≤ 200 AM, AL, ce, ES, MG, PE, PR, RS	> 200 MG, PE, PR, RS	—	—	—
BOD (mg O_2_ L^−1^)	< 60 PE, PR, RJ, RS	60 ≤ C ≤ 90 AL, AM, GO, MG, PE, PR, RJ, RS, SC, SP	> 90 ce, MS, PE, PR, RJ, RS	120	≤ 10	≤ 20
TSS (mg L^−1^)	< 100 AM, RJ, RS	100 ≤ C ≤ 150 AM, ce, ES, MG, RJ, RS	> 150 RJ, RS	—	—	≤ 30
TN (mg L^−1^)	< 10—	10 ≤ C ≤ 20 RJ, RS, SC	> 20—	—	—	—
TP (mg L^−1^)	< 1—	C = 1 RJ, RS	> 1 RS, SC	1	—	—

*Note:* (1) Restriction bands adapted from Morais and Santos (Morais and dos Santos 2019) based on Brazilian state effluent discharge standards. (2) Use after secondary treatment, disinfection, and filtration; the effluent must not contain measurable levels of pathogens. (3) Use after secondary treatment, disinfection, and filtration. C = concentration. A dash indicates that no corresponding standard was identified.

Abbreviations: AL = Alagoas; AM = Amazonas; ce = Ceará; ES = Espírito Santo; GO = Goiás; MG = Minas Gerais; MS = Mato Grosso do Sul; PE = Pernambuco; PR = Paraná; RJ = Rio de Janeiro; RS = Rio Grande do Sul; SC = Santa Catarina; and SP = São Paulo.

In the mean‐based assessment, the expressions ‘meeting a benchmark’ and ‘compatibility’ indicate only that the published system‐level mean final concentration was within the selected reference value. Means were classified as compatible when they were below the strict thresholds delimiting the very restrictive bands and at or below the upper limits adopted for the less stringent scenario.

The systems presenting the highest removal efficiencies for BOD, COD, TSS, TN, and TP were identified within each configuration. Ties and near‐ties were retained when the reported precision did not support an unambiguous ranking. The available information on media, vegetation, hydraulic conditions, HRT, aeration, treatment‐stage sequence, operating strategy, and monitoring period was then examined to identify process conditions associated with the reported performance. These comparisons were mechanistic and descriptive. A causal effect was inferred only when the source study included a controlled comparison of the corresponding design or operational variable.

Flow configuration was deliberately retained as the primary grouping criterion because it was a defining design characteristic available for all systems. Systems assigned to the same configuration were not assumed to be homogeneous experimental replicates. They differed in scale, wastewater type and strength, pretreatment, treatment function, climate, filter media, vegetation, aeration, hydraulic conditions, operating period, and monitoring design. No normalization or covariate adjustment was applied because these potential confounders were not reported consistently.

A formal meta‐analysis or random‐effects model was not applied because the source studies did not consistently provide monitoring sample sizes, standard errors, sampling variances, or directly comparable effect estimates required to quantify within‐study uncertainty. The grouped analyses were therefore not intended to estimate pooled configuration‐specific effects. They were exploratory procedures used to determine whether the reviewed system‐level distributions suggested general tendencies, dispersion, or overlap that could complement the individual‐system assessment.

Kruskal–Wallis tests were used for overall comparisons among VFCWs, HFCWs, and HCWs. When the global test was significant, Dunn pairwise tests were applied with Benjamini–Hochberg adjustment within the corresponding parameter‐specific family. The global effect size was expressed as epsilon‐squared:

(2) $$\varepsilon^{2}=\max\left[0,\frac{H-k+1}{N-k}\right]$$

where H is the Kruskal–Wallis statistic, k is the number of groups, and N is the number of systems included in the comparison. Negative estimates resulting from small samples were truncated at zero. A two‐sided significance level of 0.05 was used, but interpretation considered the adjusted *p*‐value, effect size, sample size, distributional overlap, and consistency with the system‐level observations. Confidence intervals were not estimated because the grouped comparisons were exploratory, based on small and unbalanced system‐level samples, and the source studies did not consistently report sampling variances or monitoring sample sizes required for comparable interval estimation. The tests were not interpreted as evidence of an intrinsic or causal effect of flow configuration.

### 2.3 RC Analysis

#### 2.3.1 Distributional Assumption and System‐Specific Calculations

The analytical RC formulation assumes that effluent concentrations follow a log‐normal distribution. This model has been widely applied in wastewater‐treatment reliability analyses because concentration data are non‐negative and frequently right‐skewed (Oliveira and von Sperling 2008; Dean and Forsythe 1976; Niku et al. 1979). The source studies, however, generally reported only system‐level means and SDs or CVs and did not provide the underlying monitoring series. Consequently, log‐normality could not be evaluated separately for each system–parameter combination using probability plots, goodness‐of‐fit tests, or comparisons with alternative distributions.

The analytical formulation was retained because it was developed to estimate treatment reliability from the arithmetic mean and SD through CV = SD/x̄ and therefore does not require the original time series for computation. Computational applicability does not validate the distributional assumption. The RC values, allowable means, and non‐exceedance probabilities reported in this study are conditional estimates under the log‐normal model, not distribution‐free measures. If the actual effluent distribution differs materially from the assumed log‐normal distribution, particularly in the upper tail, these estimates may be biased. They also inherit uncertainty associated with monitoring duration, sampling frequency, seasonal coverage, analytical procedures, and the accuracy of the summary statistics reported by each source.

The standard deviation on the logarithmic scale was obtained from the effluent CV as follows:

(3) $$\sigma_{\ln}={\left[\ln\left(1+{\mathrm{CV}}^{2}\right)\right]}^{\frac{1}{2}}$$

For a selected target non‐exceedance probability (1 − α), the RC was then calculated as (Oliveira and von Sperling 2008; Padalkar and Kumar 2018; Alves et al. 2021; Carvalho et al. 2022; Hamza et al. 2022; Alderson et al. 2015; Niku et al. 1979):

(4) $$\mathrm{RC}={\left(1+{\mathrm{CV}}^{2}\right)}^{\frac{1}{2}}\exp\left[-Z_{1-\alpha }\sigma_{\ln}\right]$$

where *σ*_ln_ is the standard deviation on the logarithmic scale and Z_1 − α_ is the standard normal quantile corresponding to the selected target non‐exceedance probability. The term 1 − α denotes the probability that the effluent concentration does not exceed the selected reference concentration; it is not a statistical confidence interval.

For each system–parameter combination, the probability that the final effluent concentration would not exceed a selected reference concentration was estimated from the reported mean and SD. The standard normal statistic was calculated as follows:

(5) $$Z_{s}=\frac{\ln\left(\frac{X_{s}}{\overset{`}{x}}\right)+\frac{1}{2}\ln\left(1+{\mathrm{CV}}^{2}\right)}{\sigma_{\ln}}$$

where $\bar{x}$ is the arithmetic mean final effluent concentration and $X_{s}$ is the selected discharge or reuse reference concentration. The system‐specific non‐exceedance probability was obtained as $\Phi \left(z_{s}\right)$, using the cumulative standard normal distribution.

For HCWs, the mean, SD, CV, RC, and non‐exceedance probability were calculated exclusively from the final effluent discharged by the last unit of the treatment train. Intermediate‐stage values were retained only for process characterization.

#### 2.3.2 Primary Target Probability and Probabilistic Compatibility

A target non‐exceedance probability of 95% was prespecified as the primary scenario for regulatory and design analyses, corresponding to Z_₁ ₋ α_ = 1.645. At this probability, the complete CV range observed in the dataset remained on the decreasing branch of the RC function; higher CV values therefore produced lower RC values and a more restrictive allowable mean concentration.

The RC converted the selected reference concentration into the allowable arithmetic mean effluent concentration:

(6) $$M_{x}=\mathrm{RC}\times X_{s}$$

where M_x_ is the mean effluent concentration that should not be exceeded if X_s_ is to be maintained at the selected target non‐exceedance probability. M_x_ is a system‐ and parameter‐specific operating or design target derived from the selected limit and reported relative variability; it is not an additional legal threshold. The observed and allowable means were integrated through the ratio:

(7) $$R_{m}=\frac{\overset{`}{x}}{M_{x}}$$

**FIGURE 2:**
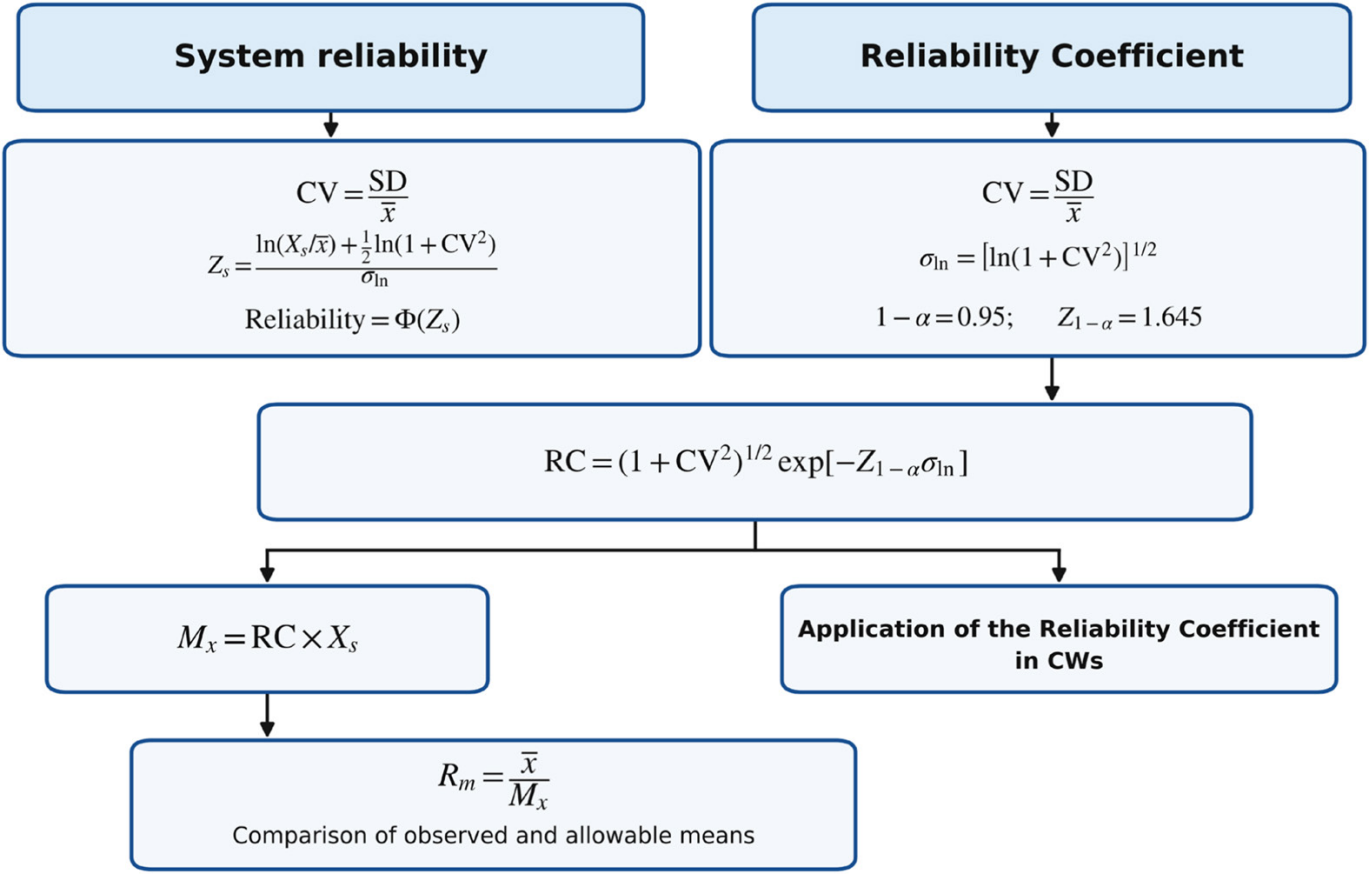
Procedure for calculating system reliability.

Values of R_m_ ≤ 1 indicate that the observed mean retained the operating margin required by the selected probability and reference concentration. Values of R_m_ > 1 indicate that the observed mean exceeded the allowable mean. This condition was defined as probabilistic incompatibility with the selected scenario, not as a formal determination of legal noncompliance. Removal efficiency, final mean concentration, RC, non‐exceedance probability, and R_m_ were interpreted as distinct performance measures (Figure 2).

Two composite regulatory scenarios were derived from the restriction bands reported by Morais and Santos (Morais and dos Santos 2019). The stringent scenario adopted limits of 60 mg O_2_ L^−1^ for BOD, 120 mg O_2_ L^−1^ for COD, 100 mg L^−1^ for TSS, 10 mg L^−1^ for TN, and 1 mg L^−1^ for TP. The less stringent scenario adopted corresponding limits of 90 mg O_2_ L^−1^ for BOD, 200 mg O_2_ L^−1^ for COD, 150 mg L^−1^ for TSS, 20 mg L^−1^ for TN, and 4 mg L^−1^ for TP. These combinations were used to examine the response of mean‐based and probabilistic compatibility to regulatory stringency and do not reproduce the complete discharge standard of a single Brazilian jurisdiction.

#### 2.3.3 Sensitivity to the Target Non‐Exceedance Probability and Ranking Stability

Sensitivity of the RC to the selected target probability was evaluated using the 121 observed CV values available across all system–parameter combinations. Each CV retained its association with the corresponding system, flow configuration, and water‐quality parameter. The observed values ranged from approximately 0.023 to 2.435 and were sorted only for graphical presentation, not as a temporal sequence. The RC was recalculated at target non‐exceedance probabilities of 50%, 60%, 70%, 80%, 90%, 95%, 98%, 99%, and 99.9%, using the corresponding standard normal quantiles. The 95% probability was retained as the primary regulatory and design scenario; the remaining probabilities were used exclusively to characterize the mathematical sensitivity of the RC and the stability of system rankings and were not treated as recommended regulatory or design targets.

The probability‐specific critical coefficient of variation was obtained analytically from Equation (S4). Ranking stability was assessed separately for BOD, COD, TSS, TN, and TP by comparing the system‐specific ordering at each probability with the 95% reference ordering. Agreement was evaluated using Spearman's rank correlation coefficient (ρ), Kendall's tau‐b (τ_b_), the number of discordant system pairs, and retention of the highest‐ranked system. Configuration‐level means were used only for scenario‐specific descriptive comparisons and were not interpreted as transferable configuration coefficients. The analytical derivation, turning‐point behavior, and complete ranking procedure are detailed in Method S1.

#### 2.3.4 Sensitivity Analyses for Wastewater Type and Source Dependence

Synthetic wastewater may be supplied under more controlled conditions than real domestic wastewater and could influence effluent variability. Its possible effect was evaluated for COD, TN, and TP, the three parameters reported for all six synthetic‐wastewater systems. BOD and TSS were not compared by wastewater type because no synthetic‐wastewater system contributed data for these parameters.

Effluent CV distributions were compared between synthetic‐ and real‐wastewater systems using exact two‐sided Mann–Whitney tests. Cliff's δ was reported as the effect‐size measure, with positive values indicating higher CVs in the synthetic‐wastewater group. Benjamini–Hochberg adjustment was applied across the COD, TN, and TP tests. At the primary 95% probability, the RC was a strictly decreasing transformation of CV over the observed range; rank‐based inference for RC was therefore equivalent to inference for CV, with the direction reversed.

Because five of the six synthetic systems were VFCWs and one was an HFCW, an exact stratified permutation test was also applied to log (CV). The synthetic‐wastewater coefficient was estimated in a linear model containing flow configuration as a fixed effect. Wastewater‐type labels were permuted within the VFCW and HFCW strata while preserving the observed number of synthetic systems in each configuration. HCWs were not included in this test because all HCWs treated real wastewater.

Potential dependence among systems reported by the same source was examined for the wastewater‐type comparison by aggregating multiple system‐level CVs from the same publication using the median and repeating the exact Mann–Whitney tests at the source level. Finally, configuration‐level RC comparisons were repeated after exclusion of all synthetic‐wastewater systems using the same Kruskal–Wallis and adjusted Dunn procedures. These analyses evaluated sensitivity to wastewater type and dataset composition; they did not remove the broader heterogeneity associated with scale, pretreatment, climate, media, loading, or operation. Detailed results are reported in Table S21.

Statistical analyses were conducted in RStudio. Deterministic RC calculations, normal‐distribution probabilities, allowable means, regulatory‐scenario calculations, and independent verification of the system‐level values were performed in Microsoft Excel.

### 2.4 Analytical Application to CW Design and Operational Monitoring

The potential incorporation of the RC into CW design and operational monitoring was evaluated as a conceptual, analytical, and exploratory framework. It was not experimentally validated as an independent sizing method. The purpose was to determine how the system‐specific allowable mean concentration derived from Equation (6) could be introduced into established hydraulic and kinetic calculations without replacing the process‐specific requirements governing CW design (von Sperling and Sezerino 2018; Associação Brasileira de Normas Técnicas (ABNT) 2024).

For HFCWs, M_x_ was substituted for the deterministic effluent target in a simplified first‐order plug‐flow model, allowing the corresponding HRT and surface area to be derived. For VFCWs, M_x_ was introduced into a calibrated areal first‐order relationship to examine its effect on allowable HLR and surface area. These analytical substitutions were considered only when the model structure and kinetic coefficient were applicable to comparable wastewater, temperature, media, loading, hydraulic, and operating conditions. The RC modified the required mean effluent concentration; it was not multiplied directly by area, HRT, HLR, or kinetic coefficients.

The hydraulic and kinetic design of HCW stages was retained as configuration‐specific. Vertical‐ and horizontal‐flow units were not collapsed into a single kinetic coefficient or weighted CV because their hydraulic regimes, oxygen transfer, redox conditions, and dominant transformation pathways differ. Stage‐specific models may be linked through the expected effluent of the upstream unit, whereas reliability of the final discharge must be assessed from the mean and dispersion of the effluent released by the last unit.

For operational interpretation, RC and R_m_ were considered as complementary indicators that could be recalculated over successive monitoring windows of comparable duration, sampling frequency, and sample size. At a fixed target probability and regulatory limit, a decrease in RC indicates increased relative variability over the monotonic range of the equation, whereas an increase in R_m_ indicates loss of the probabilistic margin because of deterioration of the mean concentration, increased variability, or both. No moving‐window analysis was performed for the literature‐derived systems because the original monitoring series were unavailable.

Neither RC nor R_m_ identifies the cause of a performance change. They may indicate loss of probabilistic margin, but diagnosis of seasonality, start‐up instability, clogging, substrate exhaustion, oxygen limitation, carbon limitation, or nutrient‐process imbalance requires complementary hydraulic, chemical, microbiological, and operational measurements. Start‐up observations should not be combined with mature operation when defining a stable baseline, and the monitoring window must contain sufficient observations to represent the upper tail of the effluent distribution.

The proposed design and monitoring framework remains conditional on the assumptions of the selected process model and on the log‐normal representation of effluent concentrations. Configuration‐level mean RC values were not treated as universal design coefficients. Long‐term validation with site‐specific data from pilot‐ and full‐scale VFCWs, HFCWs, and hybrid treatment trains is required before formal adoption in design standards.

## 3 Results and Discussion

The results are organized into three sections: (i) system characteristics and influent wastewater composition; (ii) removal performance and compatibility of final effluent quality with the selected Brazilian regulatory benchmarks; and (iii) system‐specific reliability analysis. Grouped comparisons by flow configuration were used only to describe tendencies, dispersion, and overlap within the reviewed dataset. Reliability was calculated and interpreted at the system–parameter level, and the grouped results were not used to define a universal RC for any CW configuration.

### 3.1 Characterization of the Systems and Composition of the Influent Wastewater

**FIGURE 3:**
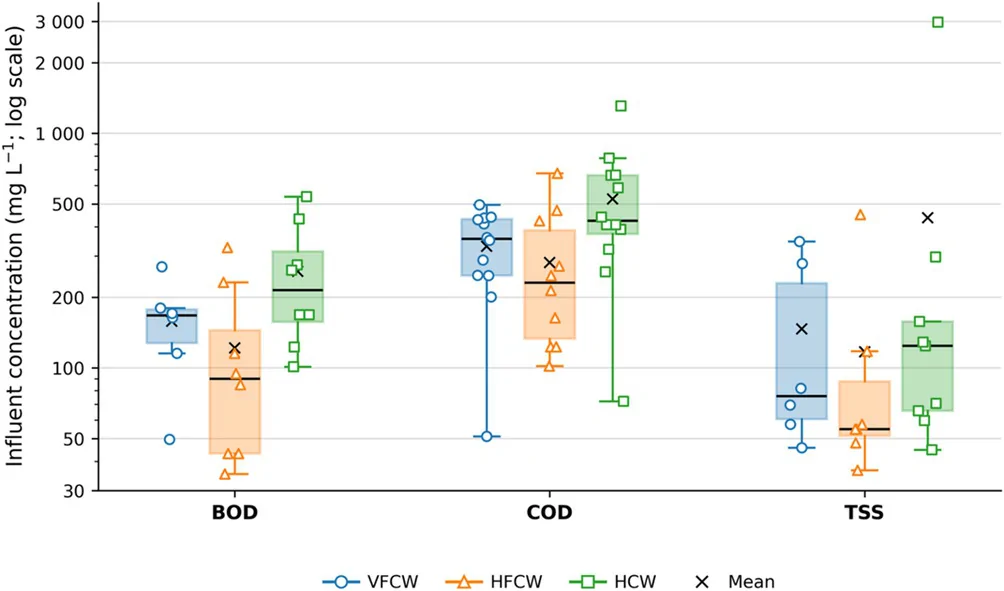
Influent BOD, COD, and TSS concentrations in VFCWs, HFCWs, and HCWs. Boxes indicate the interquartile range, horizontal lines the median, × the mean, and symbols individual systems.

**FIGURE 4:**
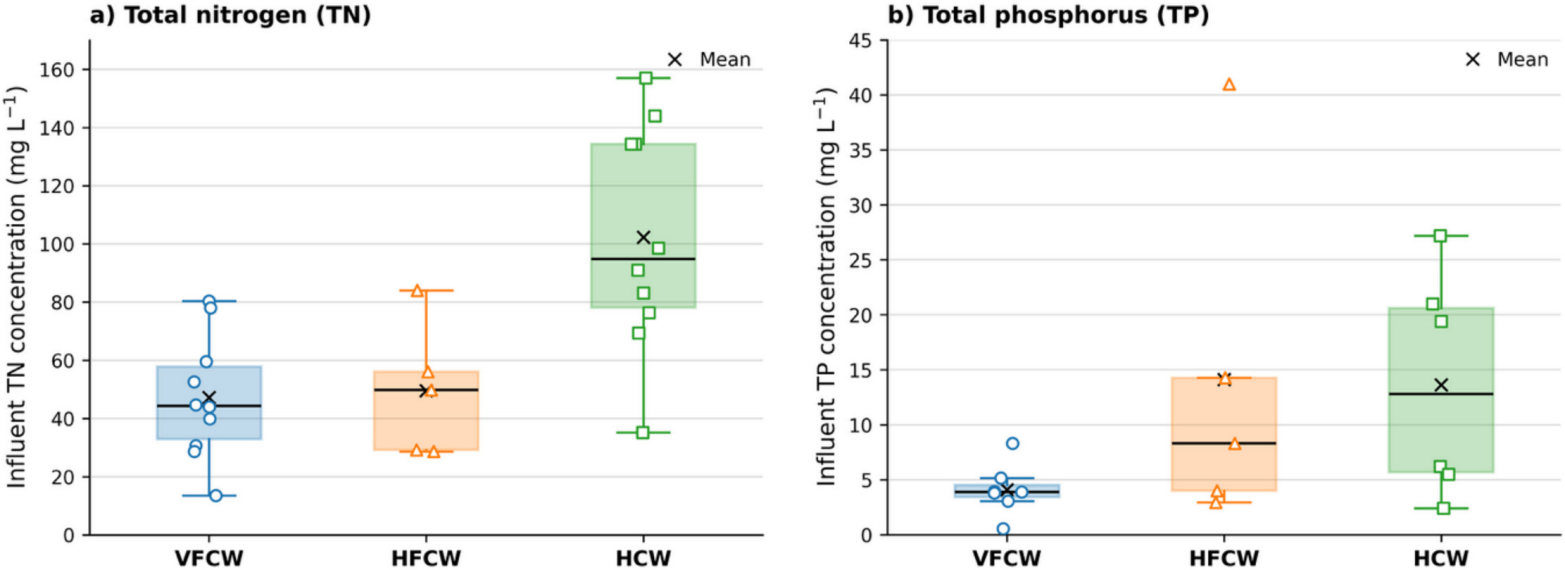
Influent concentrations of (a) total nitrogen (TN) and (b) total phosphorus (TP) in VFCWs, HFCWs, and HCWs. Boxes show the interquartile range, horizontal lines the median, × the mean, and symbols individual systems.

The dataset comprised 34 CW systems, including 12 VFCWs, 10 HFCWs, and 12 HCWs, reported across 10 countries. The studied systems treated either real domestic or urban wastewater or synthetic wastewater designed to simulate domestic sewage. Twenty‐eight systems treated real wastewater, whereas six used synthetic wastewater. The synthetic subset comprised five VFCWs and one HFCW; all HCWs treated real wastewater. All six synthetic‐wastewater systems were pilot, mesocosm, or microcosm units and contributed CV and RC data for COD, TN, and TP, but not for BOD or TSS. Most systems were applied as secondary treatment, commonly preceded by preliminary or primary treatment stages, and a smaller number were employed as tertiary treatment. The mean influent concentrations for VFCWs, HFCWs, and HCWs are presented in Figures 3 and 4, and the synthetic‐wastewater subset is detailed in Table S21. The system‐level influent concentrations are listed in Table S3, and the corresponding descriptive statistics are summarized in Table S4 (VFCWs and HFCWs) and Table S5 (HCWs).

The VFCW systems received a broad range of influent concentrations (Figure 3 and Figure 4). These systems were fed with either synthetic or real domestic wastewater originating from households, small communities, or municipal treatment facilities. In some studies, wastewater underwent pretreatment before entering the VFCW, either within the same treatment train or as part of a preceding unit, whereas other systems received untreated or prepared influent. The concentration ranges within the VFCW group therefore reflect not only vertical‐flow operation but also differences in wastewater source, upstream treatment, scale, and experimental control.

The HFCW systems received influent concentrations that largely overlapped those of the VFCWs, with lower descriptive central values for BOD, COD, and TSS and higher values for TN and TP in the reviewed dataset (Figures 3 and 4). Only one HFCW used synthetic wastewater. In the remaining systems, the wastewater underwent pretreatment, primary treatment, dilution, or secondary treatment before entering the horizontal bed. The HCWs had the highest median influent concentrations for all five parameters and the highest arithmetic means for BOD, COD, TSS, and TN. For TP, the mean concentration was slightly higher in HFCWs than in HCWs (14.10 and 13.62 mg L^−1^, respectively), whereas the median was higher in HCWs (12.81 and 8.30 mg L^−1^, respectively). The observed concentration ranges overlapped among configurations. All HCWs operated with real wastewater, and only two did not include pretreatment before the wetland units. Some systems treated wastewater from urban sewer networks consisting mainly of domestic sewage with minor commercial or light‐industrial contributions.

The systems also differed in scale, monitoring duration, filter media, vegetation, aeration strategy, climatic setting, and hydraulic conditions (Tables S1–S3). Among systems reporting these characteristics, wetland surface area ranged from approximately 0.018 to 3448 m^2^ and monitoring periods from approximately 60 to 2190 days. Filter media ranged from conventional sand and gravel to reactive or organic materials, whereas oxygen supply varied from non‐aerated or passively ventilated operation to intermittent aeration, tidal flow, and flood‐and‐drain cycles. HRT and hydraulic loading rate were not consistently reported. Flow regime was retained as the primary grouping criterion because it was consistently available and represents a defining design characteristic; however, the groups were not homogeneous experimental replicates. No normalization or covariate adjustment was possible for incompletely reported descriptors. The comparisons are therefore exploratory, and the broad within‐group dispersion in Figures 3 and 4 indicates that subsequent differences in efficiency, residual concentration, or reliability cannot be attributed exclusively to flow configuration.

### 3.2 Removal Performance and Compatibility of Final Effluent Quality With Brazilian Regulatory Benchmarks

The Brazilian regulatory references adopted in this study are expressed primarily as final effluent concentrations (Morais and dos Santos 2019; Brasil 2011; São Paulo (Estado) 2017). Removal efficiency was therefore interpreted jointly with influent and effluent concentrations. Percentage removal quantifies proportional attenuation but does not, by itself, establish compatibility with a concentration‐based criterion. A system receiving a concentrated influent may achieve high removal while retaining an effluent concentration above a stringent threshold, whereas a polishing unit treating a previously treated influent may show moderate percentage removal but a low final concentration. Accordingly, “meeting” a benchmark in this section denotes compatibility of the published system‐level mean effluent concentration with the selected reference value, not formal legal compliance. System‐level influent and effluent concentrations and removal efficiencies are reported in Table S3 (influent) and in Tables S6 and S7 (VFCWs), S8 and S9 (HFCWs), and S10 and S11 (HCWs).

Across the three configurations, descriptive mean removal efficiencies ranged from 79.8% to 94.5% for BOD, 76.9%–87.8% for COD, and 73.6%–90.8% for TSS. The corresponding ranges were 59.6%–65.9% for TN and 68.0%–75.4% for TP. These grouped means summarize the systems included in the review and were not interpreted as pooled treatment effects.

**FIGURE 5:**
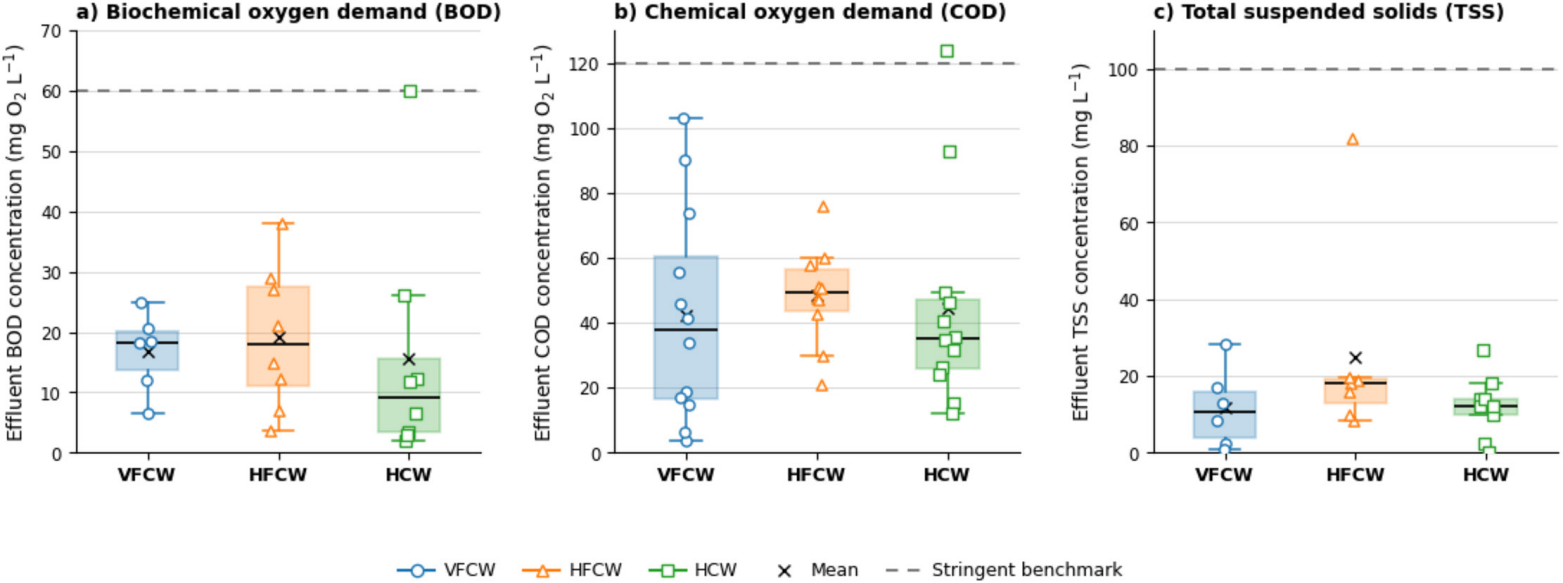
Effluent concentrations of (a) biochemical oxygen demand (BOD), (b) chemical oxygen demand (COD), and (c) total suspended solids (TSS) in VFCWs, HFCWs, and HCWs.

Figure 5 compares the final BOD, COD, and TSS concentrations with the stringent state discharge benchmarks adopted in this study.

For BOD, all six VFCWs and all eight HFCWs had mean concentrations below 60 mg O_2_ L^−1^, as did seven of the eight HCWs. The remaining HCW had a mean BOD concentration of exactly 60 mg O_2_ L^−1^ and was therefore not counted within the < 60 mg O_2_ L^−1^ band. For COD, all 12 VFCWs, all 10 HFCWs, and 11 of the 12 HCWs remained below 120 mg O_2_ L^−1^. The same HCW that reached 60 mg O_2_ L^−1^ for BOD produced a mean COD concentration of 124 mg O_2_ L^−1^. Its BOD concentration nevertheless remained below the federal limit of 120 mg O_2_ L^−1^ established by CONAMA Resolution No. 430/2011 (Brasil 2011). All 22 systems with TSS data (six VFCWs, seven HFCWs, and nine HCWs) had mean concentrations below 100 mg L^−1^.

Compatibility decreased when the concentration criteria adopted for non‐potable reuse were considered. Mean BOD concentrations were ≤ 20 mg O_2_ L^−1^ in four of six VFCWs, four of eight HFCWs, and six of eight HCWs. At ≤ 10 mg O_2_ L^−1^, the corresponding counts were one of six, two of eight, and four of eight systems. The TSS criterion of ≤ 30 mg L^−1^ was met by all six VFCWs, six of seven HFCWs, and all nine HCWs. Compatibility with these BOD and TSS values does not, by itself, establish suitability for reuse because the applicable resolution also requires treatment, filtration, disinfection, and microbiological control (São Paulo (Estado) 2017).

The broad compatibility observed for organic matter and suspended solids is consistent with the dominant treatment pathways in subsurface‐flow CWs. TSS is retained through sedimentation, interception, and filtration within the pore space, whereas biodegradable BOD and COD fractions are transformed by microbial communities attached to the media, roots, and accumulated solids (Gorgoglione and Torretta 2018; Sanchez et al. 2018; Xu et al. 2023). Intermittent feeding and drainage in VFCWs can renew oxygen within the bed and support aerobic degradation. HFCWs provide prolonged contact with media and biofilm, although oxygen availability commonly decreases along the saturated bed. In HCWs, sequential vertical and horizontal units can combine particle retention, aerobic oxidation, and downstream polishing under complementary redox conditions (Masharqa et al. 2023; Chen et al. 2024). These mechanisms are consistent with the observed performance pattern but do not isolate an intrinsic configuration effect.

Table 3 places the final concentrations in the context of influent concentration and proportional removal. The HCWs included in the dataset had the highest descriptive mean influent concentrations for BOD, COD, and TSS. These values represent concentrations rather than mass or areal loads because flow and hydraulic loading data were not reported consistently across the source studies.

**TABLE 3:** Mean system‐level influent and effluent concentrations and removal efficiencies for BOD, COD, and TSS in VFCWs, HFCWs, and HCWs.

Parameter	Mean influent concentration			Mean effluent concentration			Mean removal efficiency (%)		
	VFCW	HFCW	HCW	VFCW	HFCW	HCW	VFCW	HFCW	HCW
BOD	158.4	121.9	258.3	16.8	19.1	15.6	87.5	79.8	94.5
COD	330.0	281.6	525.1	42.1	48.4	44.4	86.6	76.9	87.8
TSS	146.7	117.2	436.6	11.6	24.8	12.1	89.8	73.6	90.8

*Note:* BOD and COD concentrations are expressed as mg O_2_ L^−1^; TSS concentrations are expressed as mg L^−1^. Means were calculated from the system‐level values reported in Tables S3 and S6–S11.

Despite higher mean influent concentrations, HCWs produced mean effluent concentrations of 15.6 mg O_2_ L^−1^ for BOD, 44.4 mg O_2_ L^−1^ for COD, and 12.1 mg L^−1^ for TSS, with corresponding mean removals of 94.5%, 87.8%, and 90.8%. VFCWs produced mean residuals of 16.8, 42.1, and 11.6 mg L^−1^, whereas HFCWs produced 19.1, 48.4, and 24.8 mg L^−1^ for BOD, COD, and TSS, respectively. HCWs therefore had the lowest descriptive mean BOD concentration, VFCWs had the lowest COD concentration, and VFCWs and HCWs produced similarly low TSS concentrations. The low residual concentrations observed in HCWs are compatible with sequential removal and polishing across multiple units. However, the HCWs also differed from the single‐stage systems in pretreatment, scale, media, vegetation, operating period, and hydraulic conditions. The pattern remains descriptive and cannot be attributed exclusively to the hybrid configuration.

**FIGURE 6:**
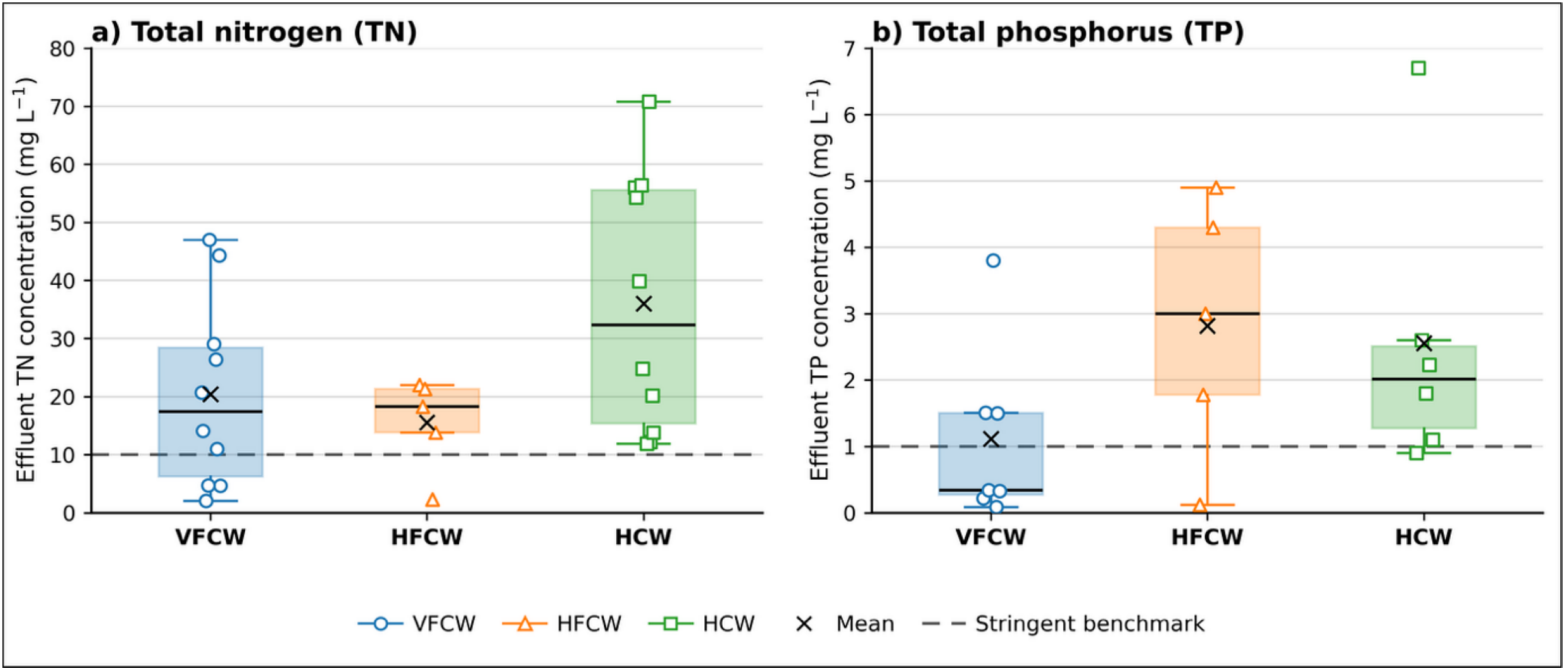
Effluent concentrations of (a) total nitrogen (TN) and (b) total phosphorus (TP) in VFCWs, HFCWs, and HCWs.

The stringent nutrient benchmarks were met less frequently. For TN, three of 10 VFCWs had mean concentrations below 10 mg L^−1^, two were between 10 and 20 mg L^−1^, and five exceeded 20 mg L^−1^. Among the five HFCWs, one was below 10 mg L^−1^, two were between 10 and 20 mg L^−1^, and two exceeded 20 mg L^−1^. None of the 10 HCWs was below 10 mg L^−1^; three were between 10 and 20 mg L^−1^, and seven exceeded 20 mg L^−1^. Thus, only four of the 25 systems with TN data had published mean concentrations below the stringent 10 mg L^−1^ benchmark. For TP, four of seven VFCWs, one of five HFCWs, and one of six HCWs had mean concentrations below 1 mg L^−1^. Overall, six of the 18 systems with TP data reached the stringent benchmark. These proportions should be interpreted cautiously because the nutrient subsets were small and unbalanced. Figure 6 compares the final TN and TP concentrations with the stringent nutrient benchmarks adopted in this study.

The grouped influent and effluent concentrations in Table 4 further show that substantial proportional nutrient removal did not necessarily produce low final concentrations, particularly when the influent was concentrated.

**TABLE 4:** Mean system‐level influent and effluent concentrations and removal efficiencies for TN and TP in VFCWs, HFCWs, and HCWs.

Parameter	Mean influent concentration			Mean effluent concentration			Mean removal efficiency (%)		
	VFCW	HFCW	HCW	VFCW	HFCW	HCW	VFCW	HFCW	HCW
TN	47.2	49.6	102.3	20.4	15.5	36.0	59.6	59.8	65.9
TP	4.1	14.1	13.6	1.1	2.8	2.6	75.4	68.0	74.3

*Note:* TN and TP concentrations are expressed as mg L^−1^. Means were calculated from the system‐level values reported in Tables S3 and S6–S11.

The HCWs included in the dataset had a mean influent TN concentration of 102.3 mg L^−1^ and the highest descriptive mean removal efficiency (65.9%), but they also retained the highest mean effluent concentration (36.0 mg L^−1^). VFCWs and HFCWs received mean influent concentrations of 47.2 and 49.6 mg L^−1^ and produced mean effluent concentrations of 20.4 and 15.5 mg L^−1^, respectively, at approximately 60% removal. The slightly higher proportional removal in HCWs did not offset their higher influent concentration. This pattern is consistent with evidence that influent TN and COD concentrations, HRT, HLR, and media characteristics jointly affect TN removal (Lam et al. 2024).

TN removal depends on the coupling of processes with different environmental requirements. Nitrification requires oxygen, suitable temperature, sufficient retention time, and an active nitrifying community, whereas denitrification requires anoxic conditions and a biodegradable electron donor (Xu et al. 2023; Fan et al. 2013; Xia et al. 2025). VFCWs commonly favor oxygen transfer and nitrification, but strongly oxic operation or limited carbon availability can constrain subsequent denitrification. Hybrid systems can combine aerobic and anoxic stages, but the presence of vertical and horizontal units does not ensure low TN residuals when stage order, recirculation, carbon availability, hydraulic retention, or influent strength are not aligned with both processes (Fan et al. 2013; Xia et al. 2025).

For TP, VFCWs received a mean influent concentration of 4.1 mg L^−1^ and produced a mean effluent concentration of 1.1 mg L^−1^ at 75.4% removal. HFCWs and HCWs received mean influent concentrations of 14.1 and 13.6 mg L^−1^ and produced mean residual concentrations of 2.8 and 2.6 mg L^−1^, at 68.0% and 74.3% removal, respectively. The lower residual concentration in VFCWs cannot be attributed solely to vertical flow because it coincided with a substantially lower influent concentration and with differences in filter media among systems.

Phosphorus is retained through sedimentation, adsorption, surface complexation, and precipitation, with additional incorporation into microbial and plant biomass. The relative contribution of these pathways depends strongly on the mineral composition and reactive surface of the media, particularly the availability of calcium, iron, and aluminum, as well as pH and redox conditions (Ran 2025). Media‐associated retention is finite, and progressive occupation of reactive sites may reduce phosphorus capture during long‐term operation. Substrate saturation was not measured consistently in the reviewed systems and therefore remains a mechanistic explanation rather than a demonstrated cause of the observed TP variability.

Grouped non‐parametric tests were used only to complement the system‐level assessment. No overall differences among configurations were detected for effluent BOD (H = 2.458, *p* = 0.293, ε^2^ = 0.024), COD (H = 2.183, *p* = 0.336, ε^2^ = 0.006), TSS (H = 2.789, *p* = 0.248, ε^2^ = 0.042), TN (H = 3.326, *p* = 0.190, ε^2^ = 0.060), or TP (H = 3.830, *p* = 0.147, ε^2^ = 0.122). The broad overlap among the distributions indicates that differences between descriptive means did not identify a configuration with consistently lower final concentrations.

For removal efficiency, overall differences were detected for BOD (H = 8.020, *p* = 0.018, ε^2^ = 0.317) and TSS (H = 8.794, *p* = 0.012, ε^2^ = 0.358). Dunn tests with Benjamini–Hochberg adjustment indicated higher BOD removal in HCWs than in HFCWs (p_adj = 0.017). For TSS, HFCWs had lower removal than VFCWs (p_adj = 0.024) and HCWs (p_adj = 0.020). COD removal approached, but did not reach the 0.05 significance level (H = 5.976, *p* = 0.050, ε^2^ = 0.128), and the corresponding pairwise comparisons were not significant after adjustment. No overall differences were detected for TN (H = 0.341, *p* = 0.843) or TP (H = 0.119, *p* = 0.942) removal. These tests describe the systems included in the review and do not estimate an intrinsic effect of flow configuration.

The comparisons remain exploratory because the systems differed in wastewater source and concentration, pretreatment, intended treatment function, scale, climate, media, vegetation, oxygen supply, hydraulic conditions, and operating period. No normalization or covariate adjustment was possible for descriptors that were incompletely reported, and the small, unbalanced group sizes limited statistical power, particularly for TN and TP. Most systems were designed and operated outside Brazil and were not intended to meet the specific combinations of Brazilian benchmarks used here. The results therefore indicate compatibility of published mean effluent concentrations with selected Brazilian reference values, not formal legal compliance, population‐level performance estimates for each configuration, or guaranteed performance under Brazilian operating conditions.

Across this heterogeneous dataset, high removal of organic matter and TSS was frequent, and nearly all published mean effluent concentrations were compatible with the selected stringent discharge benchmarks. Compatibility decreased under the more restrictive BOD reuse criteria and was markedly lower for the stringent TN and TP benchmarks. The same process characteristics also provide a mechanistic basis for the reliability patterns examined in Section 3.3. Physical retention and established biofilms can buffer short‐term variation in BOD, COD, and TSS, supporting lower effluent variability. TN removal depends on coordinated aerobic and anoxic conditions, temperature, retention time, and biodegradable carbon availability, whereas TP retention can change as reactive sites are progressively occupied. These mechanisms were not measured consistently across all systems and should therefore be interpreted as plausible explanations for parameter‐specific variability rather than as confirmed causes.

#### 3.2.1 High‐Performing CW Systems and Associated Design and Operational Features

To identify process features associated with high treatment performance without attributing the results solely to flow configuration, representative upper‐performing systems were examined (Table S26). These cases illustrate the upper range of the reviewed dataset rather than universal maxima, an exhaustive ranking, or configuration‐specific benchmarks. The systems differed in wastewater source, influent concentration, scale, pretreatment, media, vegetation, hydraulic operation, and monitoring period. Reported features were therefore interpreted as conditions associated with the observed performance. A direct causal effect was inferred only when the source study included a controlled comparison of the corresponding design or operational variable.

The upper‐performing VFCWs combined oxygen renewal with hydraulic cycling, reactive media, or controlled carbon supply. Intermittent aeration and feeding–resting cycles supported organic oxidation and nitrification, whereas controlled influent COD/N ratios and non‐aerated conditions favored the coupling of nitrification and denitrification. The upper‐performing HFCWs reflected combinations of hydraulic residence, vegetation, and filter media, with longer contact time, root‐associated biofilms, and porous or reactive media supporting biodegradation, particle retention, and nutrient removal. Among HCWs, high efficiencies were associated with sequential treatment and process complementarity: intermittently fed VF beds favored oxygen transfer, organic‐matter oxidation, and ammonium oxidation, whereas downstream HF beds provided additional filtration, polishing, and conditions more favorable to nitrate reduction. These associations were not uniform across systems and do not demonstrate an intrinsic advantage of any flow configuration.

Overall, high efficiencies were not restricted to one CW configuration and were obtained through different process‐specific combinations. Organic‐matter removal was favored by oxygen renewal, established biofilms, sufficient contact time, and staged polishing. TSS removal was associated mainly with pretreatment, granular filtration, sedimentation, and sequential beds. High TN removal required coupling ammonium oxidation with nitrate reduction while preserving an adequate carbon source, whereas TP removal depended primarily on media composition and remaining sorption capacity. Plant uptake was generally secondary to microbial transformation, filtration, and media‐associated retention for the loads represented in this dataset (Xu et al. 2023; Xia et al. 2025; Ran 2025). Because the selected systems differed substantially in scale, wastewater type, influent strength, pretreatment, climate, and operating period, the cases in Table S26 should not be used to rank VFCWs, HFCWs, and HCWs or as directly transferable design benchmarks. Their value is to identify design and operational features that can be tested under technically comparable conditions and to provide process‐based explanations for the parameter‐specific variability examined in the subsequent reliability analysis.

### 3.3 RC Analysis

#### 3.3.1 System‐Specific Non‐Exceedance Probabilities Under the Stringent Brazilian Regulatory Reference Scenario

System‐specific reliability was first evaluated as the estimated probability that the final effluent concentration would not exceed the selected stringent regulatory reference threshold. For each system–parameter combination, the probability was calculated from the reported final effluent mean, SD, and reference concentration under the assumed log‐normal distribution (Oliveira and von Sperling 2008; Padalkar and Kumar 2018; Alves et al. 2021; Carvalho et al. 2022; Hamza et al. 2022; Alderson et al. 2015; Niku et al. 1979; Marzo et al. 2018). The composite scenario adopted thresholds of 60 and 120 mg O_2_ L^−1^ for BOD and COD and 100, 10, and 1 mg L^−1^ for TSS, TN, and TP, respectively (Morais and dos Santos 2019). These thresholds delimit highly restrictive Brazilian state concentration bands and do not represent the complete discharge standard of a single jurisdiction. For HCWs, only the final effluent discharged by the last unit was used. The estimates remain conditional on the distributional assumption and on the accuracy of the reported summary statistics.

**FIGURE 7:**
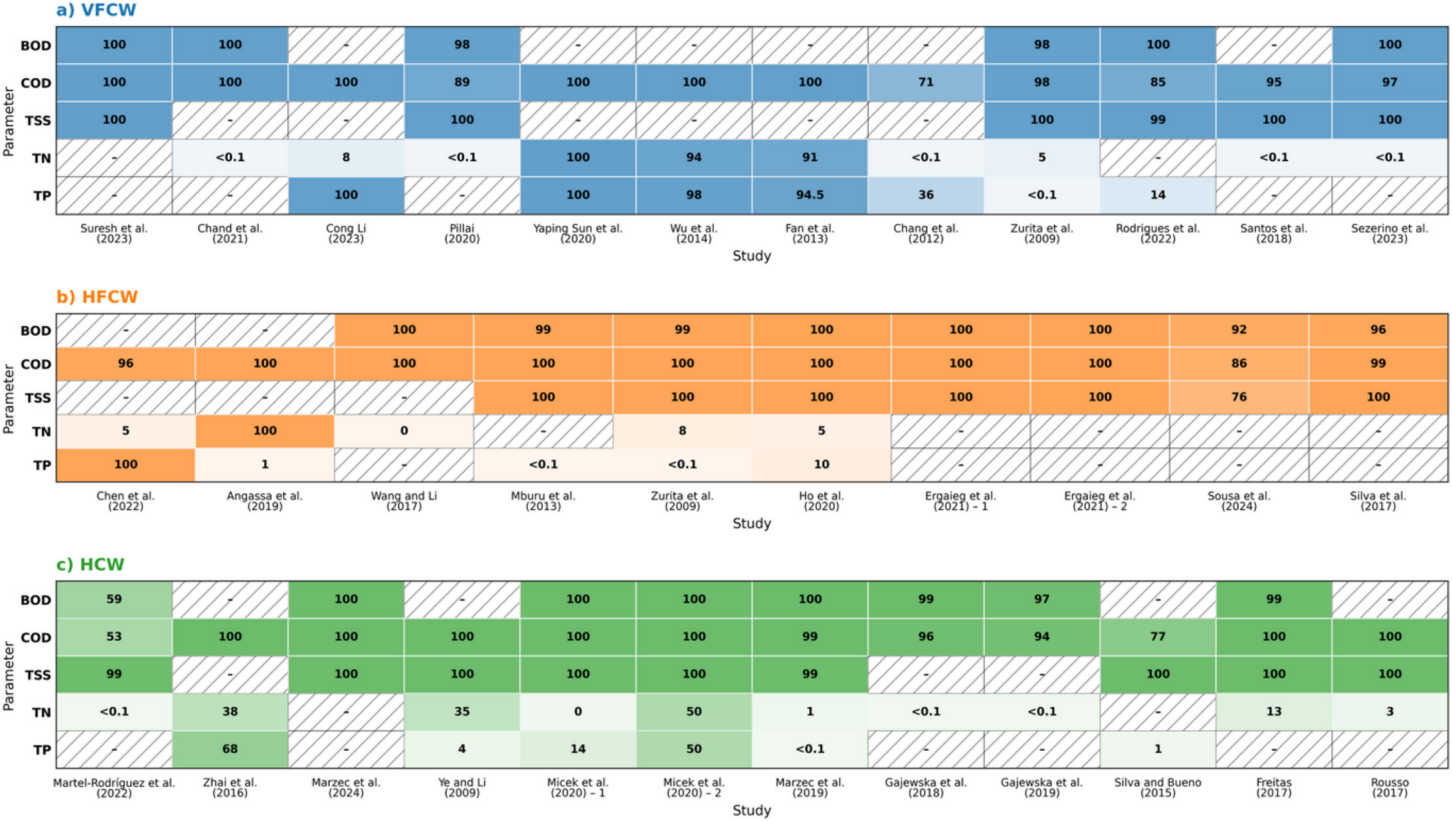
System‐specific estimated non‐exceedance probabilities (1 − α,%) for BOD, COD, TSS, TN, and TP under the stringent Brazilian regulatory reference scenario in (a) VFCWs, (b) HFCWs, and (c) HCWs. Note: Cell values show the estimated non‐exceedance probability (%); darker shading indicates a higher probability, and hatched cells indicate unavailable data. Values below 0.1% are displayed as < 0.1. Values were rounded to the nearest whole percentage, except when rounding would incorrectly indicate achievement of the 95% target. Classification relative to the 95% target was based on the unrounded probabilities. Probabilities were calculated under the assumed log‐normal distribution using the final effluent mean and coefficient of variation. For HCWs, only the final effluent discharged by the last unit of the treatment train was used.

Organic matter and suspended solids exhibited high estimated non‐exceedance probabilities in most systems (Figure 7), with values of at least 95% in 20/22 BOD, 27/34 COD, and 21/22 TSS system–parameter combinations. The standard normal deviates (Zs) and the corresponding non‐exceedance probabilities obtained for each system–parameter combination are reported in Table S12 (VFCWs), Table S13 (HFCWs), and Table S14 (HCWs). For BOD, all VFCWs and HFCWs presented probabilities of at least 92.0%, and seven of the eight HCWs exceeded 97%; the exception was the HCW evaluated by Martel‐Rodríguez et al. (2022), with 59.3%, where the mean BOD concentration equaled the 60 mg O_2_ L^−1^ threshold. COD was more dispersed, with the lowest probabilities observed for Martel‐Rodríguez et al. (2022) at 53.1%, Chang et al. (2012) at 71.1%, Silva and Bueno (2015) at 76.8%, and Rodrigues et al. (2022) and Sousa et al. (2024) at approximately 85%–86%. For TSS, only the HFCW evaluated by Sousa et al. (2024) remained below 95%, at 76.0%; all other estimates were at least 98.8%.

Nutrient reliability was markedly lower and more heterogeneous, with only 2/25 TN and 4/18 TP system–parameter combinations reaching an estimated probability of 95%. For TN, three VFCWs ranged from 91.1% to 100%, whereas the other seven did not exceed 8.3%; the HFCW evaluated by Angassa et al. (2019) reached 99.97%, whereas the remaining four ranged from 0.17% to 8.23%. No HCW reached 50%: the highest estimates were 49.96% for one system evaluated by Micek et al. (2020), 37.9% for Zhai et al. (2016), and 35.0% for Ye and Li (2009), with the remaining values at 13.0% or lower. For TP, four VFCWs ranged from 94.5% to 100%, although only three reached the 95% target, whereas the other three were 36.3% or lower. One HFCW reached approximately 100%, whereas the other four did not exceed 9.5%. HCWs ranged from less than 0.1% to 68.3%, and none attained the 95% target.

The estimated probability was jointly determined by the distance between the observed mean and X_s_ and by the relative dispersion of the effluent concentration. Means substantially below the threshold generally retained high probabilities despite moderate variability, whereas means near or above it produced narrow or absent reliability margins. Variability remained relevant below the threshold: the HFCW evaluated by Sousa et al. (2024) had a mean TSS concentration of 82 mg L^−1^, below the 100 mg L^−1^ reference value, but an SD of 33 mg L^−1^ reduced the estimated probability to 76.0% by broadening the upper tail of the assumed distribution. Mean concentration and relative dispersion must therefore be interpreted jointly.

Taken together, these results indicate wide probabilistic margins for BOD, COD, and TSS in most reviewed systems, but substantially narrower margins for TN and TP under the stringent scenario. This pattern supports the reliability of many CWs for organic‐matter and solids control but does not demonstrate resilience or an intrinsic effect of flow configuration because the systems differed in wastewater source, pretreatment, scale, climate, filter media, hydraulic operation, monitoring duration, and other design and operational attributes. The calculated probabilities also summarize aggregate variability and do not resolve seasonal, start‐up, clogging‐related, or other time‐dependent causes. The following section examines the RC at a fixed target probability of 95%, isolating the effect of the system‐specific CV on the allowable mean effluent concentration.

#### 3.3.2 Distribution of RC Among CW Configurations at a Target Non‐Exceedance Probability of 95%

**FIGURE 8:**
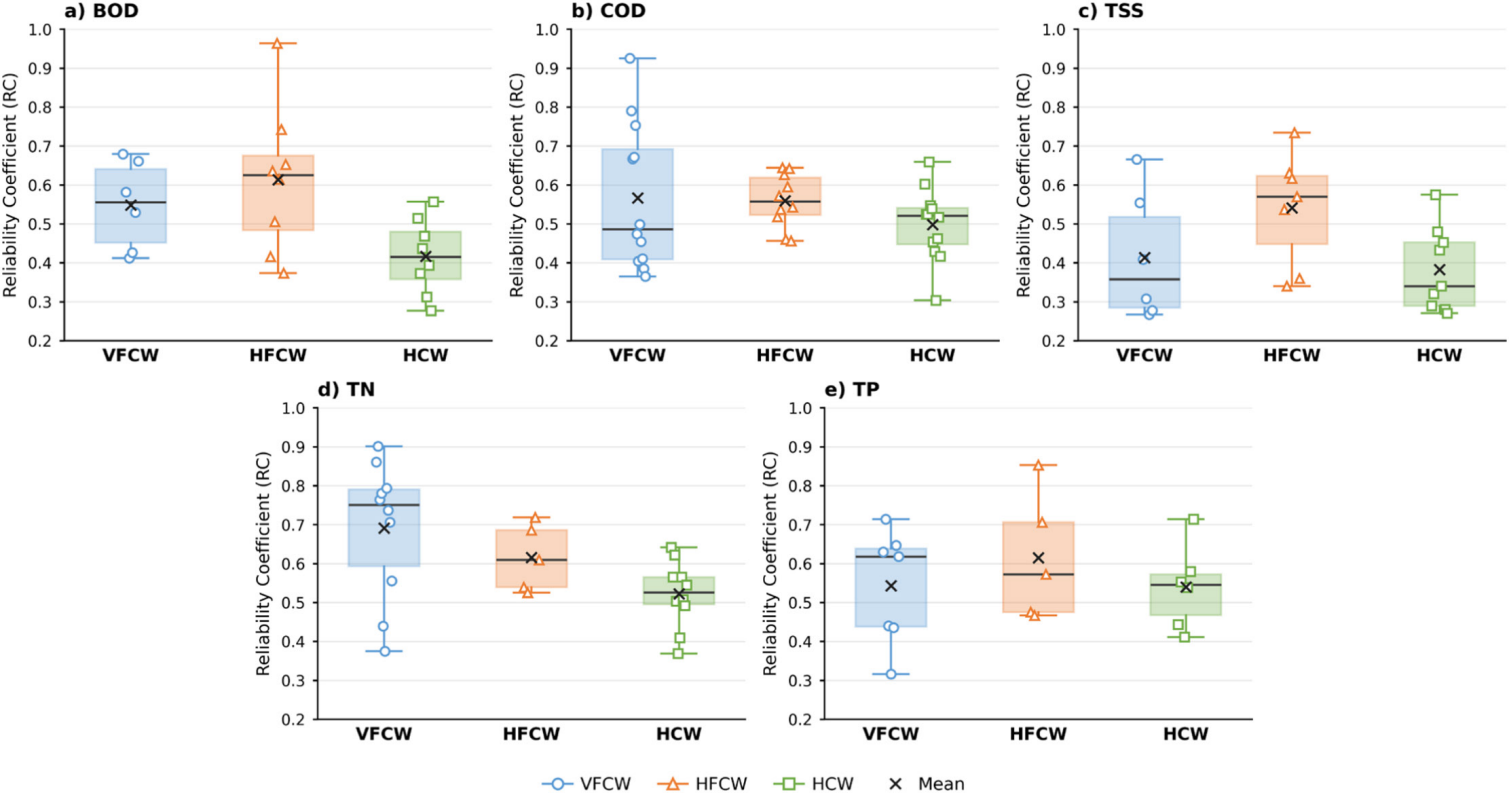
Distribution of system‐ and parameter‐specific reliability coefficients (RCs) at a target non‐exceedance probability of 95% for vertical‐flow constructed wetlands (VFCWs), horizontal‐flow constructed wetlands (HFCWs), and hybrid constructed wetlands (HCWs): (a) BOD; (b) COD; (c) TSS; (d) TN; and (e) TP. Boxes indicate the interquartile range, horizontal lines the median, x symbols the mean, and individual symbols the system‐level values.

At a target non‐exceedance probability of 95%, the RC was calculated separately for each system‐parameter combination. The RC converts the selected concentration limit $X_{s}$ into the allowable mean effluent concentration $M_{x}$ according to $M_{x}=\mathrm{RC}\times X_{s}$ (Oliveira and von Sperling 2008; Padalkar and Kumar 2018; Alves et al. 2021; Hamza et al. 2022; Alderson et al. 2015). At a fixed target probability, the RC is determined by the effluent CV and does not incorporate the observed mean concentration. It therefore quantifies the operating margin required to accommodate relative variability, rather than the non‐exceedance probability itself. For HCWs, the mean, SD, CV, and RC were calculated exclusively from the final effluent discharged by the last unit of the treatment train. The system‐specific RCs were subsequently grouped as VFCWs, HFCWs, and HCWs to examine dispersion, overlap, and exploratory configuration‐level patterns (Figure 8). These groups included systems that differed in scale, wastewater source and strength, pretreatment, climate, media, vegetation, hydraulic operation, and monitoring duration. Accordingly, the grouped distributions were not treated as homogeneous replicates or as causal estimates of an intrinsic flow‐configuration effect.

The RC varied widely among configurations, among parameters within the same configuration, and among parameters measured within individual systems. For BOD, values ranged from 0.964 in the HFCW evaluated by Ho et al. (Ho et al. 2020) (CV = 0.023) to 0.277 in the HCW evaluated by Gajewska et al. (Gajewska et al. 2018) (CV = 2.009). COD ranged from 0.925 in the VFCW assessed by Suresh et al. (Suresh et al. 2023) (CV = 0.048) to 0.303 in the same HCW of Gajewska et al. (Gajewska et al. 2018) (CV = 1.485). For TSS, the highest value was 0.734 in one of the HFCWs evaluated by Ergaieg et al. (Ergaieg et al. 2021) (CV = 0.202), whereas the lowest was 0.267 in the VFCW evaluated by Santos et al. (dos Santos et al. 2018) (CV = 2.435). TN ranged from 0.901 in the VFCW evaluated by Li et al. (Li et al. 2023) (CV = 0.065) to 0.369 in the second HCW evaluated by Micek et al. (Micek et al. 2020) (CV = 0.949). TP ranged from 0.853 in the HFCW evaluated by Mburu et al. (Mburu et al. 2013) (CV = 0.100) to 0.316 in the VFCW evaluated by Fan et al. (Fan et al. 2013) (CV = 1.333). Thus, the highest and lowest RCs were distributed among different configurations, indicating that RC magnitude was primarily system‐ and parameter‐specific.

For BOD and COD, lower CVs may reflect the capacity of the treatment train to attenuate variations in hydraulic and organic loading and to maintain comparatively stable conditions for retention and biodegradation. The highest BOD RC, reported for the HFCW evaluated by Ho et al. (Ho et al. 2020), coincided with septic‐tank pretreatment, controlled pumping, flow regulation, maintenance of a subsurface water level, and continuous contact with a stratified sand‐gravel bed. These conditions may have buffered variations in flow and particulate organic matter before and during contact with the biofilm. Conversely, the highest BOD and COD CVs occurred in the full‐scale VF‐HF system evaluated by Gajewska et al. (Gajewska et al. 2018), based on multiyear monitoring of real wastewater under seasonal and loading variation. The sequential systems evaluated by Micek et al. (Micek et al. 2020) further illustrate how primary settling, predominant oxidation in the VF bed, and subsequent HF polishing can progressively reduce the absolute dispersion of residual organic matter. This interpretation is consistent with evidence that feeding mode regulates oxygen renewal and consumption in VFCWs and that vertical and horizontal beds provide complementary kinetics for organic‐matter transformation (Gajewska et al. 2018; Chand et al. 2021).

For TSS, variability depends on the balance between physical retention and hydraulic integrity. The low CV in one of the HFCWs evaluated by Ergaieg et al. (Ergaieg et al. 2021) occurred during tertiary treatment of previously clarified municipal effluent, with comparatively low solids loading, granular media, and established vegetation. These conditions may have limited the propagation of influent fluctuations through sedimentation, interception, and filtration. Inlet blockage, solids accumulation, and preferential flow were nevertheless reported, showing that hydraulic deterioration may coexist temporarily with low effluent variability and may represent a risk of subsequent instability rather than the immediate cause of the observed CV. The high TSS CV reported by Santos et al. (dos Santos et al. 2018) was obtained in a partially saturated VFCW during a monitoring period that included the initial operating phase and intermittent feeding‐resting cycles. Bed maturation and changes in flow distribution and particle retention may have contributed to the dispersion, although the source study did not isolate these effects. Long‐term evidence from subsurface‐flow CWs similarly indicates that progressive solids and biomass accumulation can alter porosity, hydraulic conductivity, and flow distribution without causing an immediate deterioration in mean effluent quality.

For TN, effluent variability reflects the stability of the coupling between nitrification and denitrification. The low CV reported by Li et al. (Li et al. 2023) coincided with controlled synthetic wastewater, a fixed HRT, prior acclimation, and intermittent aeration, which may have reduced between‐cycle variability and maintained more regular nitrogen transformations. Fan et al. (Fan et al. 2013), by contrast, reported high mean TN removal under intermittent aeration, but the residual nitrate concentration remained highly dispersed and accounted for most of the effluent TN. Relatively small shifts in ammonium oxidation, biodegradable‐carbon availability, or nitrate reduction may therefore increase TN variability even when mean removal remains high. Aeration, recirculation, and saturation modify oxygen availability, redox gradients, and the activity of nitrifying and denitrifying communities. Stable TN performance requires sufficient nitrification while preserving the anoxic conditions and electron‐donor availability required for denitrification (Wu et al. 2015; Sezerino et al. 2023).

For TP, variability may be associated with the number, stability, and remaining capacity of the retention barriers within the treatment train. The low CV in the VF‐HF system of Silva and Bueno (Silva and Bueno 2015) occurred after two sequential mineral beds, which may have distributed phosphorus retention among filtration, sorption, and prolonged media contact. The high CV reported by Fan et al. (Fan et al. 2013) occurred in a gravel‐based VFCW with intermittent aeration, but the source study did not isolate the phosphorus‐retention mechanisms; the dispersion cannot therefore be attributed specifically to aeration, vegetation, or substrate. Chen et al. (Chen et al. 2022) provide more direct evidence: in HFCWs containing ceramsite and zeolite, neither aeration position nor plant species significantly affected TP removal, indicating that media‐related retention predominated under the evaluated conditions. Mineral composition, reactive surface area, contact time, pH, redox conditions, and cumulative phosphorus loading may therefore affect the temporal stability of TP retention. Progressive occupation of reactive sites remains a plausible source of long‐term drift, but substrate saturation should not be inferred without direct measurement because retention persistence depends on media mineralogy and local chemical conditions (Ran 2025).

The mechanistic interpretation must also account for the mathematical sensitivity of the CV when the mean effluent concentration is very low. Under these conditions, small absolute deviations represent large relative changes and may substantially reduce the RC without indicating persistently poor treatment or process deterioration. This effect was particularly evident for TSS in Santos et al. (dos Santos et al. 2018) and for TN and TP in Fan et al. (Fan et al. 2013). The mechanisms discussed above should therefore be interpreted as plausible contributors to effluent variability, whereas the influence of near‐zero means must be considered when interpreting extreme RC values.

The within‐configuration ranges further demonstrate this parameter dependence. For COD, RC values ranged from 0.365 to 0.925 in VFCWs, from 0.456 to 0.644 in HFCWs, and from 0.303 to 0.659 in HCWs. Within HCWs, the ranges were 0.277–0.557 for BOD, 0.303–0.659 for COD, 0.271–0.575 for TSS, 0.369–0.641 for TN, and 0.411–0.714 for TP. Variability therefore occurred not only among configurations but also among systems assigned to the same configuration and among parameters evaluated within the same treatment train. These distributions do not support the use of a single RC to represent an entire configuration or treatment train.

Grouped means were highest in HFCWs for BOD (0.613), TSS (0.541), and TP (0.615), and in VFCWs for COD (0.566) and TN (0.691). HCWs presented means of 0.416, 0.498, 0.382, 0.522, and 0.540 for BOD, COD, TSS, TN, and TP, respectively; the complete descriptive statistics of the RCs and CVs by parameter and configuration are presented in Table S22. Despite these numerical differences, the distributions overlapped substantially. Kruskal‐Wallis tests detected overall differences for BOD (H=6.119,p=0.0469,ε2=0.217) and TN (H=6.225,p=0.0445,ε2=0.192). For BOD, none of the Dunn pairwise comparisons remained significant after Benjamini‐Hochberg adjustment (HFCW vs. HCW, p_adj=0.0627; VFCW vs. HCW, p_adj=0.0957; VFCW vs. HFCW, p_adj=0.7755). For TN, VFCWs had higher RC values than HCWs (p_adj=0.0382), whereas the remaining pairwise comparisons were not significant (p_adj=0.3718). No overall differences were detected for COD (*p* = 0.4352), TSS (*p* = 0.0987), or TP (*p* = 0.6943). The grouped tests therefore identified no consistent configuration‐level effect across parameters.

Analyses of wastewater type did not support systematic inflation of RC under synthetic feeding. Exact Mann–Whitney and stratified permutation tests were non‐significant for COD, TN, and TP after adjustment, and source‐level aggregation produced the same conclusion. Excluding synthetic‐wastewater systems did not alter the configuration‐level conclusions for COD or TP; for TN, the difference remained significant, with higher RCs in VFCWs than in HCWs. For TP, the synthetic systems presented a higher median CV than the real‐wastewater systems (0.630 versus 0.393) and a lower median RC (0.458 versus 0.576), opposite to the pattern expected if controlled synthetic feeding systematically inflated the RC. These results confirm that grouped comparisons remain sensitive to dataset composition. Complete statistics are provided in Table S21.

The lower mean BOD RC observed in HCWs does not contradict their high removal efficiencies or low residual concentrations. Removal efficiency, mean residual concentration, and RC describe different dimensions of treatment performance. Removal efficiency quantifies proportional attenuation, the residual concentration describes mean effluent quality, and the RC defines the allowable operating mean required to accommodate relative variability at the selected target probability. Systems with similar mean residual concentrations may therefore require different operating margins when their dispersion differs. Grouped RC values should consequently be interpreted only as descriptive ranges or preliminary benchmarks for technically comparable systems, not as coefficients transferable directly to design, operation, or regulatory assessment. Practical application requires a system‐ and parameter‐specific RC derived from data representative of the actual loading, climatic, hydraulic, operating, and bed‐maturation conditions.

The absolute RC values are also conditional on the target non‐exceedance probability. For a fixed CV, a higher target probability decreases the RC and $M_{x}$, thereby increasing the margin required between the expected mean performance and $X_{s}$. The 95% scenario was used here as a common probabilistic benchmark, rather than as a universal value that should be adjusted to accommodate the observed performance of a system. Selection of a target probability should reflect the consequences of exceedance, receiving‐water sensitivity, intended effluent use, data uncertainty, monitoring frequency, and the technical capacity to control the treatment process (Oliveira and von Sperling 2008; Hamza et al. 2022; Alderson et al. 2015; Lombard‐Latune et al. 2020; Niku et al. 1979). Because the RC does not include the observed mean, it cannot establish probabilistic compatibility in isolation; that assessment additionally requires $\frac{\bar{x}}{M_{x}}$ or the estimated non‐exceedance probability. The following section evaluates how the RC and the system ranking respond to alternative target non‐exceedance probabilities.

#### 3.3.3 Sensitivity of the RC to the Target Non‐Exceedance Probability

The preceding analysis used a target non‐exceedance probability of 95% as the common reference for all system–parameter combinations. The sensitivity analysis applied the 121 observed effluent CVs, ranging from 0.023 to 2.435, individually to the RC equation at target probabilities from 50% to 99.9%. This analysis evaluates sensitivity to the selected target probability.

**FIGURE 9:**
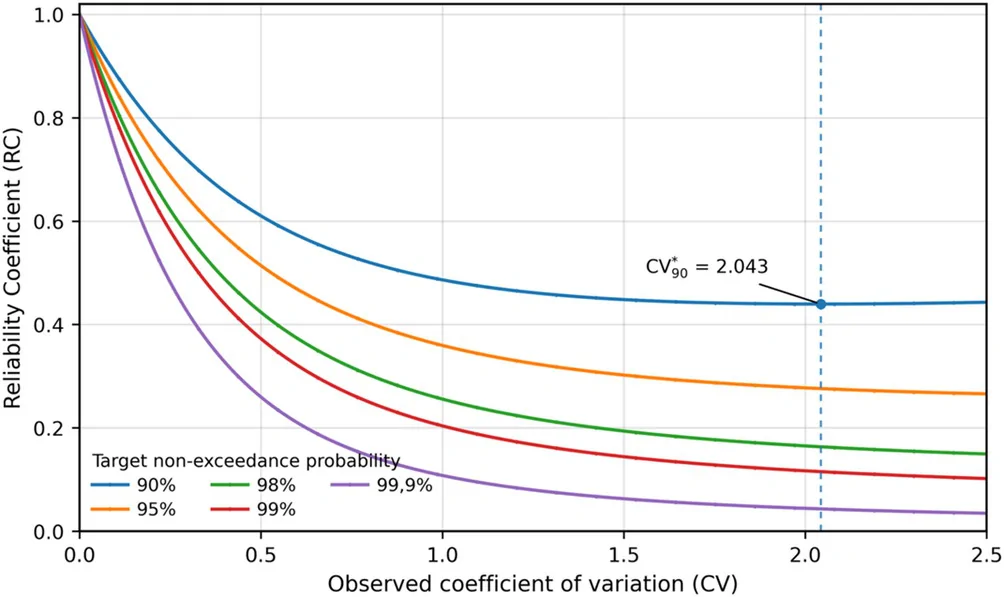
Relationship between the reliability coefficient (RC) and the observed coefficient of variation (CV) at target non‐exceedance probabilities of 90%, 95%, 98%, 99%, and 99.9%.

At target probabilities of 95%, 98%, 99%, and 99.9%, the critical CVs exceeded the maximum observed CV of 2.435. Consequently, the complete dataset remained on the decreasing branch of the function, and higher CVs consistently produced lower RCs (Figure 9).

At 90%, 119 of the 121 system–parameter combinations remained on the decreasing branch. For any fixed CV, increasing the target probability reduced the RC and the allowable mean effluent concentration (M_x_), thereby increasing the required margin below the regulatory limit. At lower target probabilities, the critical CV occurred within the observed range, producing a non‐monotonic response and, in some scenarios, RC values greater than one. These values do not denote greater stability, improved effluent quality, or enhanced pollutant removal.

The complete system ordering was identical at target probabilities of 95% and above. At 90%, the ranking remained unchanged for BOD, COD, TN, and TP and showed only a limited departure for TSS. RC values should therefore be compared only when calculated at the same target non‐exceedance probability. Complete turning‐point results, responses at 50%–80%, rank correlations, discordant system pairs, configuration‐level descriptive means, and scenario‐specific rankings are provided in Method S1, Figures S1 and S2, and Table S23.

The 95% scenario was retained for the subsequent regulatory and design analyses because the complete observed CV range remained on the decreasing branch and the system ordering was stable at that probability and above. Irrespective of the selected probability, however, the RC does not incorporate the observed mean concentration and cannot establish probabilistic compatibility in isolation. The following section therefore combines the RC‐derived allowable mean with the observed effluent mean and the selected regulatory reference limits.

#### 3.3.4 Mean‐Based and Probabilistic Compatibility Under Stringent and Less Stringent Brazilian Regulatory Reference Scenarios

The preceding analyses separated the effect of relative effluent variability from the observed mean concentration. Here, these components were integrated to assess probabilistic compatibility with the two Brazilian regulatory reference scenarios derived from the state restriction bands reported by Morais and Santos (Morais and dos Santos 2019). At the target non‐exceedance probability of 95%, the system‐ and parameter‐specific RC converted the selected concentration limit into the allowable mean effluent concentration $M_{x}=\mathrm{RC}\times X_{s}$. Probabilistic compatibility was then expressed through the observed‐to‐allowable mean ratio, R_m_ = x̄/M_x_. Under the assumed log‐normal model, the criterion is mathematically equivalent to an estimated non‐exceedance probability of at least 95% for the same system–parameter combination, reference limit, and target probability. It provides an operational and design‐oriented representation of the distance between the observed mean and the allowable mean. Unlike the RC alone, R_m_ incorporates the observed mean concentration, effluent variability, selected target probability, and regulatory limit (Oliveira and von Sperling 2008; Padalkar and Kumar 2018; Alves et al. 2021; Hamza et al. 2022; Alderson et al. 2015). Values of R_m_ > 1 indicate that the observed mean exceeded the allowable mean required by the selected scenario.

All calculations were performed separately for each system‐parameter combination. For HCWs, the mean, SD, CV, RC, allowable mean, and observed‐to‐allowable mean ratio were derived exclusively from the final effluent discharged by the last unit of the treatment train. The stringent scenario adopted BOD, COD, TSS, TN, and TP limits of 60, 120, 100, 10, and 1 mg L^−1^, respectively, whereas the less stringent scenario adopted 90, 200, 150, 20, and 4 mg L^−1^. These combinations were used as comparative reference scenarios and do not reproduce the complete requirements of a single state regulation. The complete system‐level calculations are provided in Tables S15 and S16 (VFCWs), S17 and S18 (HFCWs), and S19 and S20 (HCWs), for the stringent and less stringent scenarios, respectively.

**FIGURE 10:**
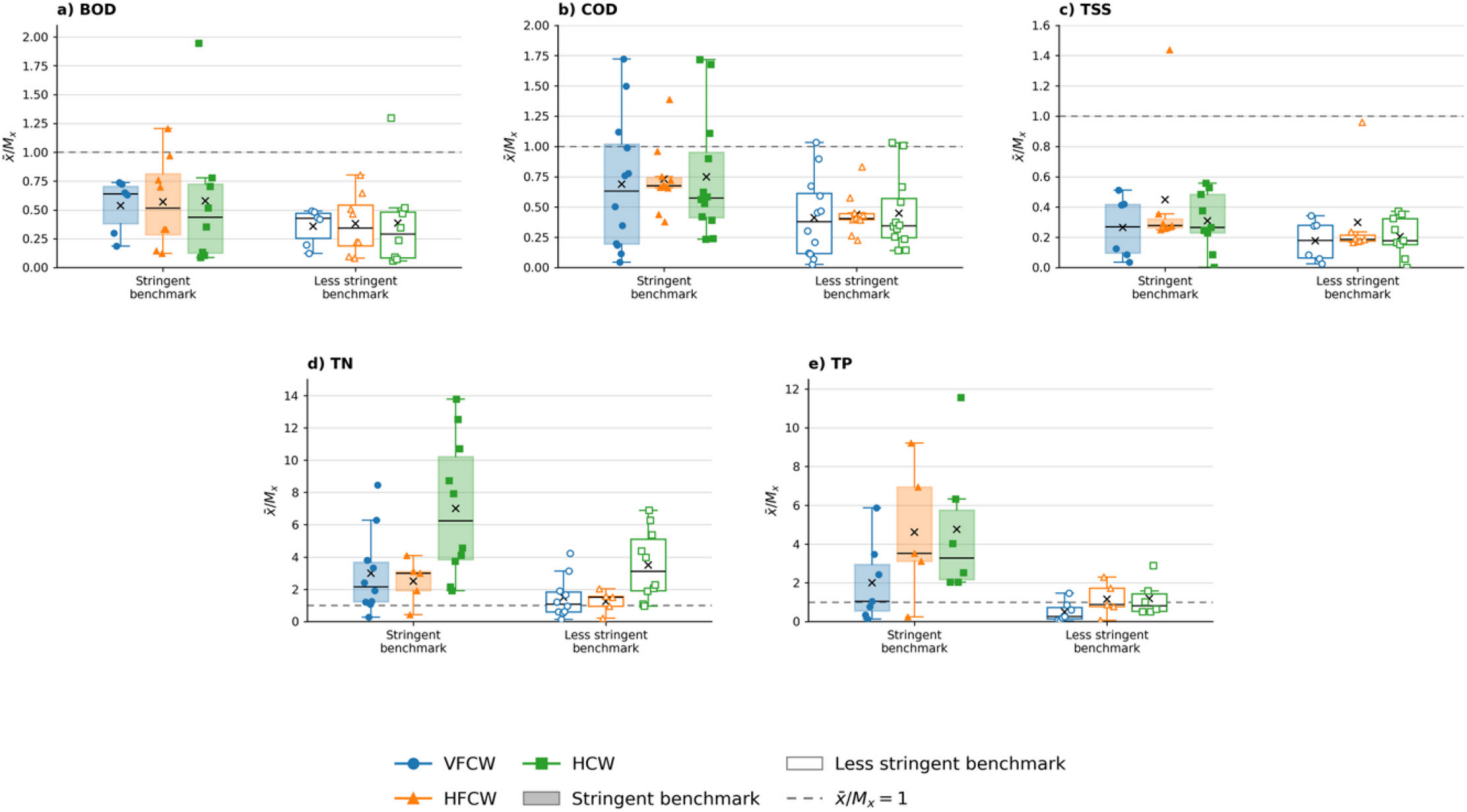
Distribution of the ratio between the observed mean effluent concentration ($\bar{x}$) and the allowable mean effluent concentration ($M_{x}$) at a target non‐exceedance probability of 95% under the stringent and less stringent Brazilian regulatory reference scenarios. Values of $\frac{\bar{x}}{M_{x}}\leq 1$ indicate probabilistic compatibility with the selected scenario, whereas values of $\frac{\bar{x}}{M_{x}}>1$ indicate that the observed mean exceeded the allowable mean.

The distributions of $\bar{x}/M_{x}$ are shown in Figure 10. The horizontal reference line at unity separates system–parameter combinations that retained the allowable operating margin from those for which the observed mean exceeded the allowable mean. The paired scenarios show that increasing the concentration limit reduced $\bar{x}/M_{x}$ but did not necessarily produce probabilistic compatibility when the observed mean remained close to the threshold or the effluent CV was high.

For organic matter and suspended solids, mean‐based compatibility was high, but the probabilistic assessment identified additional system‐parameter combinations without sufficient operating margin. Under the stringent scenario, final means met the concentration criterion in 21 of 22 systems for BOD, 33 of 34 for COD, and all 22 for TSS. After the 95% target and effluent variability were incorporated, the corresponding counts decreased to 20/22, 27/34, and 21/22. The largest difference occurred for COD: six systems with means below 120 mg O_2_ L^−1^ did not satisfy the allowable mean derived from their individual CVs.

Under the less stringent scenario, all final means met the BOD, COD, and TSS concentration criteria, whereas probabilistic compatibility was observed in 21/22, 31/34, and 22/22 systems, respectively (Table 5).

**TABLE 5:** Comparison between mean‐based compatibility, determined from the final mean effluent concentration, and probabilistic compatibility at a target non‐exceedance probability of 95% under the stringent and less stringent Brazilian regulatory reference scenarios.

Parameter	*n*	Stringent reference scenario		Less stringent reference scenario	
		Final mean within reference limit	Probabilistically compatible at 95%	Final mean within reference limit	Probabilistically compatible at 95%
BOD	22	21/22 (95.5%)	20/22 (90.9%)	22/22 (100%)	21/22 (95.5%)
COD	34	33/34 (97.1%)	27/34 (79.4%)	34/34 (100%)	31/34 (91.2%)
TSS	22	22/22 (100%)	21/22 (95.5%)	22/22 (100%)	22/22 (100%)
TN	25	4/25 (16.0%)	2/25 (8.0%)	11/25 (44.0%)	8/25 (32.0%)
TP	18	6/18 (33.3%)	4/18 (22.2%)	15/18 (83.3%)	12/18 (66.7%)

*Note:* Mean‐based compatibility followed the inequalities used to define each scenario: final means were counted as compatible when they were below the stringent thresholds and at or below the upper limits adopted for the less stringent scenario. Probabilistic compatibility was defined as $\frac{\bar{x}}{M_{x}}\leq 1$.

One HCW had a final mean COD concentration of 93.0 mg O_2_ L^−1^, below the 200 mg O_2_ L^−1^ reference threshold, but its system‐specific RC yielded $M_{x}=92.39$ mg O_2_ L^−1^ and $\bar{x}/M_{x}=1.007$. Thus, mean‐based compatibility did not translate into compatibility with the 95% target. The difference was small in absolute terms but analytically relevant because the classification is defined by the unity threshold (Table 5).

The separation between mean‐based and probabilistic compatibility was more pronounced for nutrients. Under the stringent scenario, only four of 25 systems with TN data and six of 18 with TP data had final means below the corresponding reference thresholds. After variability was incorporated, only 2/25 TN systems and 4/18 TP systems satisfied the allowable mean associated with the 95% target. Under the less stringent scenario, mean‐based compatibility increased to 11/25 for TN and 15/18 for TP, whereas probabilistic compatibility increased to 8/25 and 12/18, respectively. One HCW with a mean TP concentration of 2.23 mg L^−1^ had $M_{x}=2.210$ mg L^−1^ and $\bar{x}/M_{x}=1.009$ under the 4 mg L^−1^ reference scenario.

These results occurred despite substantial mean removal efficiencies. Mean TN removal was 60% in VFCWs, 60% in HFCWs, and 66% in HCWs, whereas mean TP removal was 75%, 68%, and 74%, respectively. The corresponding mean residual concentrations were 20.4, 16, and 36 mg L^−1^ for TN and 1.1, 2.8, and 2.6 mg L^−1^ for TP. Thus, removals of approximately 60%–75% did not consistently reduce nutrient concentrations sufficiently, or provide an adequate margin below the reference threshold, to sustain a 95% non‐exceedance target. The result reflects both the relatively high influent nutrient concentrations described in Section 3.2 and the greater process dependence of sustained TN and TP removal.

Removal efficiency, final mean concentration, RC, and $\frac{\bar{x}}{M_{x}}$ describe distinct dimensions of performance. Removal efficiency quantifies proportional attenuation, the final mean describes average effluent quality, and the RC defines the downward adjustment of the regulatory limit required to accommodate the reported relative variability. The ratio $\frac{\bar{x}}{M_{x}}$ integrates these quantities for a selected limit and target probability. Consequently, favorable mean or high removal efficiency does not ensure probabilistic compatibility, and an RC cannot establish compatibility without the observed mean. Values above unity indicate that the residual mean, variability, or both must be reduced, but they do not identify the underlying process limitation.

Probabilistic compatibility at 95% should therefore be interpreted as one line of evidence related to system robustness, not as a complete measure of robustness itself. The analysis does not quantify recovery after disturbances, identify causal resistance mechanisms, or resolve temporal trajectories. Within this narrower interpretation, the stringent scenario was compatible with 90.9% of systems for BOD, 79.4% for COD, and 95.5% for TSS, compared with only 8.0% for TN and 22.2% for TP. The less stringent scenario increased compatibility, particularly for TP, but TN remained the principal limitation.

The subset of eight CWs operated in Brazil showed the same broad pattern but also confirmed that the less stringent scenario did not guarantee compatibility for every organic‐matter result. All available Brazilian final means met the stringent concentration criteria for BOD (5/5), COD (8/8), and TSS (8/8). After variability was incorporated, probabilistic compatibility under the stringent scenario was observed in 4/5 systems for BOD, 5/8 for COD, and 7/8 for TSS. Under the less stringent scenario, compatibility increased to 5/5 for BOD, 7/8 for COD, and 8/8 for TSS; one Brazilian HCW remained slightly above the allowable COD mean, with $\frac{\bar{x}}{M_{x}}=1.007$. None of the four Brazilian systems with TN data met either probabilistic scenario, whereas the two systems with TP data failed the stringent target but met the less stringent target. These results are traceable to the system‐level values in Tables S15–S20.

The Brazilian subset was small and parameter availability was unequal. More broadly, 26 of the 34 systems were operated outside Brazil under different climatic, hydraulic, operational, and regulatory conditions and were not designed for the composite benchmark combinations adopted here. The results therefore indicate probabilistic compatibility with selected Brazilian reference scenarios under the log‐normal assumption, rather than formal legal compliance or guaranteed performance under Brazilian conditions. They also do not support a universal RC for VFCWs, HFCWs, or HCWs or a configuration‐independent claim of reliability.

Taken together, the mean‐based and probabilistic results support a comparatively wide operating margin for organic matter and suspended solids in most evaluated systems, whereas probabilistic compatibility with stringent TN and TP thresholds remained less frequent. For individual systems, a ratio above unity provides an operational or design signal that may require a lower residual concentration, reduced variability, or both. The following section examines how this system‐specific probabilistic margin can be incorporated into CW design calculations and operational monitoring without treating the RC as a direct safety factor or a stand‐alone diagnostic indicator.

#### 3.3.5 Potential Application of the RC to CW Design, Operation, and Maintenance

The RC can support two complementary decisions in constructed wetlands: defining an allowable mean effluent concentration during design and tracking changes in the probabilistic operating margin during operation. The framework proposed here is conceptual, analytical, and exploratory; it has not been experimentally validated as an independent sizing procedure. Its purpose is to connect a selected target non‐exceedance probability and system‐specific effluent variability to established hydraulic and kinetic calculations, while preserving the process constraints that govern CW design and maintenance (Oliveira and von Sperling 2008; Padalkar and Kumar 2018; Alves et al. 2021; Carvalho et al. 2022; Hamza et al. 2022; Alderson et al. 2015; Niku et al. 1979; Marzo et al. 2018).

During design, the RC establishes a margin between the selected discharge or reuse threshold and the mean concentration that the treatment process should be designed to achieve. As defined in Equation (6), $M_{x}=\mathrm{RC}\times X_{s}$, where $X_{s}$ is the regulatory reference limit and $M_{x}$ is the allowable mean effluent concentration at the selected target probability. The RC is therefore not a conventional safety factor multiplied directly by area, HRT, or HLR. Instead, it modifies the effluent concentration target supplied to the design model. At a 95% target non‐exceedance probability and over the CV range observed in this study, a higher CV produces a lower RC and a more restrictive allowable mean.

The RC should be estimated separately for each system–parameter combination from monitoring data representative of the expected wastewater, loading regime, climate, pretreatment, media, maturation stage, and operating strategy. For a new installation, a value transferred from a comparable pilot‐ or full‐scale system should be treated only as an initial design assumption. It should be updated when site‐specific monitoring data become available. Configuration‐level mean RCs reported in Section 3.3.2 are descriptive summaries and should not be inserted directly into design equations without demonstrating technical comparability.

For a HFCW, $M_{x}$ can replace the deterministic effluent target in a first‐order model when the model structure and kinetic coefficient have been calibrated under comparable conditions (von Sperling and Sezerino 2018; Associação Brasileira de Normas Técnicas (ABNT) 2024; Marzo et al. 2018). For a simplified plug‐flow representation with negligible background concentration, the concentration relationship is:

(8) $$\frac{M_{x}}{C_{i}}=\exp\left(-k_{v}t\right)$$

Solving Equation (8) for the required HRT gives:

(9) $$t=\frac{\ln\left(\frac{C_{i}}{M_{x}}\right)}{k_{v}}$$

The corresponding wetland area is:

(10) $$A=\frac{\mathrm{Qt}}{\mathrm{hn}}$$

where $C_{i}$ is the design influent concentration, $k_{v}$ is the volumetric first‐order coefficient, t is the required HRT, Q is the design flow, h is the effective bed depth, and n is the effective porosity. Reducing $M_{x}$ increases the logarithmic concentration term in Equation (9) and, all other variables held constant, increases both HRT and area. The RC does not modify $k_{v}$; it modifies the required mean effluent concentration. The resulting area must still satisfy hydraulic and organic loading constraints, headloss, media gradation, oxygen availability, inlet distribution, and clogging control.

Direct incorporation into VFCW design is more limited because these systems are commonly sized from surface hydraulic and organic loadings, intermittent dosing, resting periods, drainage, and oxygen‐transfer requirements (von Sperling and Sezerino 2018; Associação Brasileira de Normas Técnicas (ABNT) 2024). A single first‐order expression does not reproduce the alternating saturated and unsaturated conditions or the operational cycles that control oxygen renewal. Nevertheless, when an areal first‐order model has been calibrated for the specific vertical‐flow regime, the allowable mean may be introduced as:

(11) $$\frac{M_{x}}{C_{i}}=\exp\left(\frac{-k_{a}}{q}\right)$$

Solving Equation (11) for the allowable surface HLR gives:

(12) $$q=\frac{k_{a}}{\ln\left(\frac{C_{i}}{M_{x}}\right)}$$

and the corresponding surface area is

(13) $$A=\frac{Q}{q}$$

where $k_{a}$ is the areal first‐order coefficient and q is the surface HLR. A lower $M_{x}$ reduces the allowable HLR and increases the area. This relationship does not determine dosing frequency, resting intervals, number of beds, drainage capacity, oxygen demand, or the degree of bed saturation. These variables remain governed by the hydraulic, organic, and operational criteria specific to VFCWs. When sizing is based on surface organic loading, oxygen transfer, or per‐capita area, the RC‐adjusted target should be used as a performance verification rather than as a substitute for those design criteria.

A complete numerical example illustrating the mathematical effect of replacing the regulatory limit with the RC‐adjusted allowable mean is provided in Supporting Information Method S2. For constant kinetic and hydraulic coefficients, the HFCW HRT and area would be 42.4% higher when the RC‐adjusted target is used. In the areal VFCW model, the allowable HLR would be 29.8% lower and the area 42.4% higher. These percentages express only the mathematical effect of replacing 60 mg O_2_ L^−1^ with 36 mg O_2_ L^−1^; they are not design recommendations and do not account for background concentration, temperature correction, loading limits, oxygen demand, or hydraulic constraints. The additional area is not imposed arbitrarily by the RC; it results from the lower mean concentration required to accommodate the assumed variability at the selected probability.

For HCWs, the kinetic and hydraulic design of each stage should remain configuration‐specific. A vertical unit and a horizontal unit should not be collapsed into a single first‐order coefficient or a weighted CV because their hydraulic regimes and dominant transformation pathways differ. Stage‐specific models may be linked sequentially through the expected effluent from the upstream unit, whereas the RC used for final regulatory assessment should be calculated from the mean and dispersion of the effluent discharged by the last unit of the treatment train. This preserves the distinction between process design within the train and reliability assessment of the final discharge.

During operation, the RC can be recalculated over successive monitoring windows of comparable duration, sampling frequency, and sample size. At a fixed target probability, regulatory limit, and within the CV range evaluated at 95%, a decrease in RC indicates an increase in relative effluent variability. The RC should be interpreted jointly with the observed‐to‐allowable mean ratio: $R_{m}=\frac{\bar{x}}{M_{x}}$.

Values of $R_{m}\leq 1$ indicate that the observed mean remains at or below the allowable mean, whereas $R_{m}>1$ indicates incompatibility with the selected target probability. A trend in RC does not establish a process failure or identify its cause. Operational interpretation requires a site‐specific definition of meaningful change based on analytical uncertainty, sampling variability, seasonality, and the expected range during stable operation. The joint interpretation of RC and $R_{m}$ is summarized in Table 6.

**TABLE 6:** Interpretation of RC trends and observed‐to‐allowable mean ratio (R_m_).

RC trend	R_m_	Interpretation
Stable	≤ 1	Effluent variability and the probabilistic margin remain similar, and the observed mean remains within the allowable concentration.
Decreasing	≤ 1	Relative variability has increased and the allowable mean has decreased, but the observed mean still satisfies the selected target probability.
Increasing	≤ 1	Relative variability has decreased and the allowable mean has increased. The operating margin has improved provided that the observed mean has not increased concurrently.
Stable	> 1	At similar variability, the observed mean exceeds the allowable mean. Mean effluent quality is the principal limitation, although the regulatory threshold may not yet be exceeded.
Decreasing	> 1	Variability has increased and the observed mean exceeds the allowable mean. The deviation may reflect an increase in the mean, a decrease in the allowable mean, or both.
Increasing	> 1	Variability has decreased, but the observed mean remains above the allowable mean. Additional reduction of the residual concentration is required.

*Note:* “Stable” indicates no change beyond the expected analytical and sampling variability established for the monitoring program. Interpretations assume a fixed target probability, the same regulatory limit, comparable monitoring windows, and an adequate distributional model. RC trends do not independently identify the cause of the observed change.

A sustained reduction in RC can provide an early signal of increasing variability before the observed mean exceeds the allowable mean. Conversely, a stable or increasing RC combined with $R_{m}>1$ indicates that reducing the mean concentration is more important than reducing variability. In both cases, the indicators only identify loss of probabilistic margin. Hydraulic, operational, chemical, and microbiological measurements are required to diagnose the underlying mechanism.

Common CW limitations may produce different combinations of mean deterioration and increased variability. Parameter‐specific RC‐based signals, diagnostic limitations, and complementary operational or maintenance responses are provided in Table S25.

Where monitoring records are sufficient, RC and $R_{m}$ should be calculated for comparable seasonal or operational windows rather than for an undifferentiated long‐term series. Start‐up observations should not be combined with mature operation when the objective is to define a stable baseline. The monitoring window must contain enough samples to represent the upper tail of the effluent distribution; otherwise, apparent changes in RC may reflect sampling density rather than process behavior.

The proposed design and monitoring framework remains conditional on the assumptions of the selected first‐order model and on the log‐normal representation of effluent concentrations. Kinetic coefficients must be calibrated for comparable temperature, loading, media, hydraulic regime, and operating conditions. Because the original monitoring series were unavailable, goodness‐of‐fit could not be verified for the systems compiled in this study, and transferring an RC between systems assumes similar temporal variability. The sensitivity analysis quantified the effect of target probability but did not resolve distributional uncertainty. Consequently, the RC should be interpreted as a probabilistic adjustment of the required mean effluent concentration and as a complementary monitoring indicator, not as a stand‐alone sizing method or a causal diagnostic tool. Validation with long‐term, site‐specific data from pilot‐ and full‐scale VFCWs, HFCWs, and hybrid treatment trains is required before formal adoption in design standards.

## 4 Conclusions

The analysis of 34 constructed wetland systems showed that flow configuration alone did not explain the observed differences in removal efficiency, residual effluent concentration, or reliability. VFCWs, HFCWs, and HCWs differed in wastewater type and strength, pretreatment, scale, climate, media, vegetation, aeration, hydraulic conditions, and monitoring duration, and their performance distributions overlapped substantially. Dataset heterogeneity therefore represents both a methodological limitation and a relevant finding: treatment performance resulted from system‐specific interactions among influent characteristics, design, and operation. Accordingly, configuration‐level comparisons describe the systems included in this review but should not be interpreted as estimates of an intrinsic effect of flow regime.

Organic matter and suspended solids showed the widest performance margins. Mean removal efficiencies ranged from 79.8% to 94.5% for BOD, from 76.9% to 87.8% for COD, and from 73.6% to 90.8% for TSS. Mean effluent concentrations were below the stringent discharge benchmarks in 21 of 22 systems for BOD, 33 of 34 for COD, and all 22 systems for TSS. When effluent variability and a target non‐exceedance probability of 95% were incorporated, probabilistic compatibility was maintained in 20 of 22, 27 of 34, and 21 of 22 systems, respectively. Compatibility was lower under the reuse‐related criteria: 14 of 22 systems achieved BOD ≤ 20 mg O_2_ L^−1^, seven achieved BOD ≤ 10 mg O_2_ L^−1^, and 21 achieved TSS ≤ 30 mg L^−1^. These concentration‐based results do not establish suitability for reuse without the filtration, disinfection, and microbiological controls required for the intended application.

Nutrient performance showed a clearer separation between percentage removal and final effluent quality. Mean removal efficiencies ranged from 59.6% to 65.9% for TN and from 68.0% to 75.4% for TP, yet only four of 25 systems had mean TN concentrations below 10 mg L^−1^ and six of 18 had mean TP concentrations below 1 mg L^−1^. Under the same limits and the 95% target probability, probabilistic compatibility decreased to two of 25 systems for TN and four of 18 for TP. The upper‐performing systems indicated that high removal was associated with pollutant‐specific process conditions: oxygen renewal, established biofilms, and sequential polishing for organic matter; physical retention and hydraulic integrity for TSS; coordinated nitrification and denitrification for TN; and reactive media with remaining sorption capacity for TP. These mechanisms are consistent with the observed patterns but cannot be interpreted as causal explanations across all systems. The results therefore do not identify a universally superior configuration; performance depends on whether the design and operating conditions support the processes required for the target pollutant.

The central outcome of the reliability analysis was that a single RC cannot represent a CW configuration, an entire treatment train, or the technology as a whole. At a target non‐exceedance probability of 95%, system‐ and parameter‐specific RCs ranged from 0.267 to 0.964, with the lowest and highest values distributed among different configurations. Grouped mean RCs were highest in HFCWs for BOD, TSS, and TP and in VFCWs for COD and TN, but the distributions overlapped widely and no consistent configuration effect was identified across the five parameters. Grouped RCs should therefore be interpreted only as descriptive distributions or preliminary benchmarks for technically comparable systems, not as transferable design or regulatory coefficients. The RC quantifies the adjustment of the allowable mean concentration required to accommodate relative effluent variability at a selected target probability. It is not a direct measure of removal efficiency, mean effluent quality, the actual non‐exceedance probability, or overall technological robustness and must be interpreted jointly with the observed mean concentration, the applicable limit, and either the observed‐to‐allowable mean ratio or the estimated non‐exceedance probability.

Selection of the target non‐exceedance probability directly affects both the magnitude and the interpretation of the RC. For a fixed CV, a higher target probability decreases the RC and the allowable mean concentration, thereby increasing the performance margin required below the regulatory limit. This more conservative criterion may reduce the risk of exceedance and promote more robust design and operational control, but it does not, by itself, increase the intrinsic robustness of the treatment technology. Conversely, lower target probabilities increase the allowable mean and reduce the margin of protection, which may be inappropriate when exceedance could compromise the intended effluent use, receiving‐water quality, or environmental protection. The sensitivity analysis further showed that the conventional inverse relationship between CV and RC remained valid throughout the observed CV range only at target probabilities of 95% or higher. At 90%, this behavior was retained for 119 of the 121 system–parameter combinations, whereas at 80% the complete ranking changed for BOD, COD, TSS, and TP. At probabilities of 70%, 60%, and 50%, ranking reversals became frequent, and numerically higher RCs could occur at higher CVs because of the mathematical behavior of the equation rather than improved treatment stability. RC values calculated at different target probabilities are therefore not directly comparable, and high RCs obtained under low probability requirements should not be interpreted as evidence of greater reliability or robustness.

For design applications, the RC may be used to convert a selected regulatory limit into a system‐ and parameter‐specific allowable mean concentration, which may subsequently be incorporated into calibrated kinetic or hydraulic models. It should not be treated as a conventional safety factor applied directly to wetland area, HRT, or hydraulic loading rate. Under operational conditions, RC trends and the observed‐to‐allowable mean ratio may help identify loss of performance margin when evaluated over comparable monitoring windows, but neither indicator diagnoses the underlying process disturbance. Such diagnosis still requires assessment of loading, hydraulics, oxygen transfer, temperature, media condition, clogging, and other process variables. The estimates remain conditional on the untested log‐normal assumption and inherit uncertainty from monitoring duration, sampling frequency, analytical procedures, small and unbalanced parameter subsets, and the summary statistics reported in the source studies. Because 26 of the 34 systems were operated outside Brazil, the regulatory results indicate compatibility with the Brazilian benchmark scenarios adopted in this study rather than formal legal compliance or guaranteed performance under Brazilian conditions. Within these constraints, the findings support broad probabilistic margins for organic matter and TSS in most evaluated systems, but not a universal claim of CW robustness, whereas sustained TN and TP compatibility remained dependent on influent strength, process integration, operating conditions, and regulatory stringency.

## Author Contributions

**Clélio Rodrigo Paiva Rafael:** investigation, writing – original draft, formal analysis, conceptualization, methodology. **Joan Garcia:** writing – review and editing, visualization. **Eduardo Lucas Subtil:** conceptualization, funding acquisition, writing – review and editing, methodology, visualization, validation, supervision, project administration.

## Funding

This work was supported by Fundação de Amparo à Pesquisa do Estado de São Paulo (10.13039/501100001807; 2023/18242‐2), Conselho Nacional de Desenvolvimento Científico e Tecnológico (10.13039/501100003593; 402618/2022‐0), and Coordenação de Aperfeiçoamento de Pessoal de Nível Superior (10.13039/501100002322; 001).

## Conflicts of Interest

The authors declare no conflicts of interest.

## Supporting information


**Table S1:** Study context and design, treatment, and operational characteristics of the vertical‐flow constructed wetlands (VFCWs; *n* = 12), horizontal‐flow constructed wetlands (HFCWs; *n* = 10), and hybrid constructed wetlands (HCWs; *n* = 12) included in the analysis.
**Table S2:** System description, study objective, role within the treatment train, treatment‐enhancement strategy, intended effluent use, and flow rate for the 26 constructed wetland systems for which these data were available.
**Table S3:** Mean influent concentrations of biochemical oxygen demand (BOD), chemical oxygen demand (COD), total suspended solids (TSS), total nitrogen (TN), and total phosphorus (TP) in the VFCW, HFCW, and HCW systems included in the analysis.
**Table S4:** Descriptive statistics for influent BOD, COD, TSS, TN, and TP concentrations in the VFCW and HFCW systems. Sample size varied among parameters according to data availability.
**Table S5:** Descriptive statistics for influent BOD, COD, TSS, TN, and TP concentrations in the HCW systems. Sample size varied among parameters according to data availability.
**Table S6:** Mean effluent concentrations and corresponding removal efficiencies for BOD, COD, TSS, TN, and TP in the VFCW systems.
**Table S7:** Descriptive statistics for effluent BOD, COD, TSS, TN, and TP concentrations and their corresponding removal efficiencies in the VFCW systems.
**Table S8:** Mean effluent concentrations and corresponding removal efficiencies for BOD, COD, TSS, TN, and TP in the HFCW systems.
**Table S9:** Descriptive statistics for effluent BOD, COD, TSS, TN, and TP concentrations and their corresponding removal efficiencies in the HFCW systems.
**Table S10:** Mean effluent concentrations and corresponding removal efficiencies for BOD, COD, TSS, TN, and TP in the HCW systems.
**Table S11:** Descriptive statistics for effluent BOD, COD, TSS, TN, and TP concentrations and their corresponding removal efficiencies in the HCW systems.
**Table S12:** Standard normal deviate (Zs) and corresponding non‐exceedance probability (1 − α) for BOD, COD, TSS, TN, and TP in the VFCW systems relative to the most stringent state discharge limits adopted in the analysis.
**Table S13:** Standard normal deviate (Zs) and corresponding non‐exceedance probability (1 − α) for BOD, COD, TSS, TN, and TP in the HFCW systems relative to the most stringent state discharge limits adopted in the analysis.
**Table S14:** Standard normal deviate (Zs) and corresponding non‐exceedance probability (1 − α) for BOD, COD, TSS, TN, and TP in the HCW systems relative to the most stringent state discharge limits adopted in the analysis.
**Table S15:** Mean effluent concentration (x̄), reliability coefficient (RC), coefficient of variation (CV), allowable mean effluent concentration (m_x_), and observed‐to‐allowable mean concentration ratio (x̄/m_x_) for BOD, COD, TSS, TN, and TP in the VFCW systems at a target non‐exceedance probability of 95%, considering regulatory limits of 60, 120, 100, 10, and 1 mg L^−1^, respectively.
**Table S16:** Mean effluent concentration (x̄), reliability coefficient (RC), coefficient of variation (CV), allowable mean effluent concentration (m_x_), and observed‐to‐allowable mean concentration ratio (x̄/m_x_) for BOD, COD, TSS, TN, and TP in the VFCW systems at a target non‐exceedance probability of 95%, considering the less stringent benchmark limits adopted in this study: 90, 200, 150, 20, and 4 mg L^−1^, respectively.
**Table S17:** Mean effluent concentration (x̄), reliability coefficient (RC), coefficient of variation (CV), allowable mean effluent concentration (m_x_), and observed‐to‐allowable mean concentration ratio (x̄/m_x_) for BOD, COD, TSS, TN, and TP in the HFCW systems at a target non‐exceedance probability of 95%, considering regulatory limits of 60, 120, 100, 10, and 1 mg L^−1^, respectively.
**Table S18:** Mean effluent concentration (x̄), reliability coefficient (RC), coefficient of variation (CV), allowable mean effluent concentration (m_x_), and observed‐to‐allowable mean concentration ratio (x̄/m_x_) for BOD, COD, TSS, TN, and TP in the HFCW systems at a target non‐exceedance probability of 95%, considering the less stringent benchmark limits adopted in this study: 90, 200, 150, 20, and 4 mg L^−1^, respectively.
**Table S19:** Mean effluent concentration (x̄), reliability coefficient (RC), coefficient of variation (CV), allowable mean effluent concentration (m_x_), and observed‐to‐allowable mean concentration ratio (x̄/m_x_) for BOD, COD, TSS, TN, and TP in the HCW systems at a target non‐exceedance probability of 95%, considering regulatory limits of 60, 120, 100, 10, and 1 mg L^−1^, respectively.
**Table S20:** Mean effluent concentration (x̄), reliability coefficient (RC), coefficient of variation (CV), allowable mean effluent concentration (m_x_), and observed‐to‐allowable mean concentration ratio (x̄/m_x_) for BOD, COD, TSS, TN, and TP in the HCW systems at a target non‐exceedance probability of 95%, considering the less stringent benchmark limits adopted in this study: 90, 200, 150, 20, and 4 mg L^−1^, respectively.
**Table S21:** Participation of synthetic‐wastewater systems and sensitivity analyses of effluent coefficient of variation (CV) and reliability coefficient (RC) results to wastewater type.
**Table S22:** Descriptive statistics of mean reliability coefficients (RCs) and coefficients of variation (CVs) by parameter and constructed wetland configuration at a target non‐exceedance probability of 95%.
**Figure S1:** Relationship between the reliability coefficient (RC) and the observed coefficient of variation (CV) at target non‐exceedance probabilities of 50%, 60%, 70%, and 80%.
**Figure S2:** Mean reliability coefficient (RC) by constructed wetland configuration and target non‐exceedance probability for (a) BOD, (b) COD, (c) TSS, (d) TN, and (e) TP.
**Table S23:** Mean reliability coefficient (RC) by parameter, constructed wetland configuration, and target non‐exceedance probability.
**Table S24:** Traceability of the constructed wetland systems included in the analytical dataset, showing the source, exact wetland configuration, information extracted, and DOI or full‐text access link.
**Table S25:** RC‐based signals, diagnostic limitations, and complementary operational or maintenance responses for common constructed‐wetland limitations.
**Table S26:** Representative upper‐performing constructed wetland systems and the main design or operational features associated with their reported removal efficiencies.

## Data Availability

The data that support the findings of this study are available from the corresponding author upon reasonable request.
