# Supplementary material for: Reliability‐Based Evaluation of the Robustness of Constructed Wetlands for Sustainable Sanitation: Implications for Design and Operation
Source: Water Environ Res. 2026 Sep 16;98(9):e70572. doi: 10.1002/wer.70572 (PMC13579613; doi:10.1002/wer.70572)
Supplement: Supplementary file 1 [file WER-98-e70572-s001.docx]

**Supplementary Materials**

**Reliability-based evaluation of the robustness of constructed wetlands for sustainable sanitation: implications for design and operation**

Clélio Rodrigo Paiva Rafael ^1^; Joan Garcia²,^3^; Eduardo Lucas Subtil^1*^

^1^Laboratory of Urban Wastewater Treatment and Water Reuse (LabTAUS), Center of Engineering, Modeling and Applied Social Sciences, Federal University of ABC, Santo André/SP, Brazil. E-mail: [eduardo.subtil@ufabc.edu.br](mailto:eduardo.subtil@ufabc.edu.br) and [clelio_rodrigo10@hotmail.com](mailto:clelio_rodrigo10@hotmail.com)

^2^ GEMMA - Group of Environmental Engineering and Microbiology, Department of Civil

and Environmental Engineering, Escola d’Enginyeria de Barcelona Est (EEBE), Universitat

Politècnica de Catalunya·BarcelonaTech, Av. Eduard Maristany 16, Building C5.1, E-

08019 Barcelona, Spain. E-mail: [joan.garcia@upc.edu](mailto:joan.garcia@upc.edu)

^3^ GEMMA - Group of Environmental Engineering and Microbiology, Department of Civil

and Environmental Engineering, Department of Civil and Environmental Engineering,

Universitat Politècnica de Catalunya·BarcelonaTech, c/ Jordi Girona 1-3, Building D1, E-

08034 Barcelona, Spain.

*Corresponding author: Eduardo Lucas Subtil.

Laboratory of Urban Wastewater Treatment and Water Reuse, Federal University of ABC, Av. dos Estados, 5001, São Paulo 09210-580, Brazil. E-mail address: eduardo.subtil@ufabc.edu.br

References are cited in the author–year format and are listed alphabetically at the end of this Supporting Information. Part of these references is also cited in the main manuscript.

Note: BOD, biochemical oxygen demand; COD, chemical oxygen demand; TSS, total suspended solids; TN, total nitrogen; TP, total phosphorus; CW, constructed wetland; VFCW, vertical-flow constructed wetland; HFCW, horizontal-flow constructed wetland; HCW, hybrid constructed wetland; RC, reliability coefficient; CV, coefficient of variation; SD, standard deviation; HRT, hydraulic retention time; HLR, hydraulic loading rate; VF, vertical-flow stage; HF, horizontal-flow stage; WWTP, wastewater treatment plant; WSP, waste stabilization pond; FWS, free-water-surface wetland; COD/N, chemical oxygen demand-to-nitrogen ratio; Xₛ, regulatory limit; Mₓ, allowable mean effluent concentration at the target non-exceedance probability; Rₘ = x̄/Mₓ, observed-to-allowable mean concentration ratio; —, not reported or not applicable. BOD and COD concentrations are expressed as mg O₂ L⁻¹; TSS, TN, and TP concentrations are expressed as mg L⁻¹. Calculations were performed using unrounded values, whereas the values shown in the tables were rounded for presentation. An Rₘ value ≤ 1 indicates that the observed mean concentration met the allowable mean target at the specified reliability level.

**System characterization and data availability — Tables S1 and S2.**

Tables S1 and S2 summarize the design, treatment, and operational characteristics of the 34 constructed wetland systems included in the analysis. The systems were grouped according to flow configuration as vertical-flow constructed wetlands (VFCWs; n = 12), horizontal-flow constructed wetlands (HFCWs; n = 10), and hybrid constructed wetlands (HCWs; n = 12). “–“ indicates that the corresponding information was not reported in the original study.

Table S1: Study context and design, treatment, and operational characteristics of the vertical-flow constructed wetlands (VFCWs; n = 12), horizontal-flow constructed wetlands (HFCWs; n = 10), and hybrid constructed wetlands (HCWs; n = 12) included in the analysis.

| **Author** | **CW configuration** | **Country** | **Pretreatment** | **Treatment level** | **Wastewater type** | **Substrate type** | **Vegetation type** | **Area (m²)** | **Operating period (days)** |
| --- | --- | --- | --- | --- | --- | --- | --- | --- | --- |
| Suresh et al., 2023 | VFCW | India | Septic tank | Secondary | Real | Sand and gravel | Canna indica (Indian shot) | 4 | 365 |
| Chand et al., 2021 | VFCW | India | – | Secondary | Real | Sand, gravel, and biochar | Typha sp. (cattail) | 0.09 | 120 |
| Li et al., 2023 | VFCW | China | WWTP supernatant | Secondary | Synthetic | Gravel and washed sand | Iris pseudacorus (yellow flag iris) | 0.02 | < 90 |
| Pillai and Nair, 2021 | VFCW | India | Screening at a WWTP | Secondary | Real | Gravel, coarse river sand, and coco peat (coconut fiber) | Cumbu Napier hybrid grass (Pennisetum purpureum) | 0.29 | < 300 |
| Sun et al., 2021 | VFCW | China | – | Tertiary | Synthetic | Biochar, activated carbon, and coarse sand | Pennisetum sinese Roxb. | 0.49 | 180 |
| Wu et al., 2015 | VFCW | China | – | Secondary | Synthetic | Gravel | Phragmites australis (common reed) | 0.44 | < 120 |
| Fan et al., 2013 | VFCW | China | – | Secondary | Synthetic | Gravel | Phragmites australis (common reed) | 0.44 | 150 |
| Chang et al., 2012 | VFCW | China | – | Secondary | Synthetic | Gravel | Canna indica and Pontederia cordata | 4 | 154 |
| Zurita et al., 2009 | VFCW | Mexico | Prefiltration | Secondary | Real | Tezontle gravel | Strelitzia reginae (bird-of-paradise); Anthurium andreanum (anthurium); Agapanthus africanus (agapanthus) | 3.24 | 365 |
| Rodrigues et al., 2022 | VFCW | Brazil | – | Secondary | Real | Biochar | Eucalyptus benthamii | 3.10 | 210 |
| Santos et al., 2018 | VFCW | Brazil | Septic tank | Secondary | Real | Coarse sand | Typha domingensis | 7.5 | 630 |
| Sezerino et al., 2023 | VFCW | Brazil | Septic tank | Secondary/advanced | Real | Gravel and sand | Typha domingensis | 7.5 | 2190 |
| Chen et al., 2022 | HFCW | China | – | Secondary | Synthetic^1^ | Zeolite and ceramsite | Iris sibirica | 0.48 | 120 |
| Angassa et al., 2019 | HFCW | Ethiopia | Pretreatment | Secondary | Real | Gravel | Phragmites karka | 3.5 | < 300 |
| Wang and Li, 2017 | HFCW | China | Anaerobic tank and infiltration devices | Secondary | Real | – | – | 0.6 | 730 |
| Mburu et al., 2013 | HFCW | Kenya | Primary treatment in a WWTP facultative pond | Secondary | Real | Granite gravel | Cyperus papyrus | 22.5 | 60 |
| Zurita et al., 2009 | HFCW | Mexico | Pretreatment | Secondary | Real | Tezontle gravel | Strelitzia reginae (bird-of-paradise); Anthurium andreanum (anthurium); Agapanthus africanus (agapanthus) | 3.24 | 365 |
| Ho et al., 2020 | HFCW | Vietnam | Septic tank and dilution | Secondary | Real | Gravel and sand | Brachiaria mutica (para grass or Angola grass) | 0.48 | 90 |
| Ergaieg et al., 2021 | HFCW | Tunisia | Secondary treatment | Tertiary | Real^1^ | Fine gravel | Phragmites australis (common reed) | 670 | 1095 |
| Ergaieg et al., 2021 | HFCW | Tunisia | Secondary treatment | Tertiary | Real | Fine gravel | Phragmites australis (common reed) | 430.5 | 1095 |
| Sousa et al., 2024 | HFCW | Brazil | Primary treatment | Secondary | Real | Sand and gravel | Zizanopsis bonariensis | 72 | 365 |
| Silva et al., 2017 | HFCW | Brazil | Preliminary and primary treatment | Secondary | Real | Sand, gravel, polystyrene beads (Styrofoam beads), and crushed stone | Vetiver grass (Chrysopogon zizanioides) | 40.5 | 63 |
| Martel-Rodríguez et al., 2022 | HCW | Spain | Septic tank and Imhoff tank | Secondary | Real | Gravel | Typha latifolia (cattail) | 650 | 2190 |
| Zhai et al., 2016 | HCW | China | Septic tank and two sedimentation lagoons | Tertiary | Real | Gravel | Cyperus alternifolius (emergent ornamental plant) | 3448 | 1095 |
| Marzec et al., 2024 | HCW | Poland | Three-chamber septic tank | Secondary and tertiary | Real | Sand | Miscanthus giganteus (giant miscanthus) and Salix viminalis (willow) | 140 | 1825 |
| Ye and Li, 2009 | HCW | China | Septic tank | Secondary | Real | Gravel and sand | – | 134.5 | 365 |
| Micek et al., 2020 | HCW | Poland | Three-chamber settling tank | Tertiary | Real | Sand | Phragmites australis (common reed) and Salix viminalis (willow) | 48 | 1095 |
| Micek et al., 2020 | HCW | Poland | Three-chamber settling tank | Tertiary | Real | Sand | Phragmites australis (common reed) and Salix viminalis (willow) | 96 | 1095 |
| Marzec et al., 2019 | HCW | Poland | Primary settling tank | Secondary | Real | Sand | Miscanthus giganteus (giant miscanthus) and Helianthus tuberosus (Jerusalem artichoke) | 176 | 1825 |
| Gajewska et al., 2018 | HCW | Poland | – | Secondary | Real | Sand and gravel | Salix viminalis (willow) | 48 | 1460 |
| Gajewska et al., 2018 | HCW | Poland | – | Secondary | Real | Sand and gravel | Phragmites australis (common reed) and Salix viminalis (willow) | 48 | 1460 |
| Silva and Bueno, 2015 | HCW | Brazil | Preliminary treatment | Secondary | Real | Crushed stone | Emergent macrophytes | 7.2 | 98 |
| Freitas, 2017 | HCW | Brazil | Settler–digester | Secondary | Real | Gravel and sand | Typha domingensis | 23.49 | 546 |
| Rousso, 2017 | HCW | Brazil | Septic tank | Secondary | Real | Sand and gravel | Typha spp. (cattail) | 25 | 730 |

Table S2: System description, study objective, role within the treatment train, treatment-enhancement strategy, intended effluent use, and flow rate for the 26 constructed wetland systems for which these data were available.

| **AUTHOR** | **DESCRIPTION** | **OBJECTIVE** | **CW CHARACTERISTICS** | **TREATMENT STRATEGIES** | **EFFLUENT END USE** | **FLOW RATE (m³ d⁻¹)** |
| --- | --- | --- | --- | --- | --- | --- |
| Suresh et al., 2023 | The treatment system comprises a three-chamber septic tank (the third chamber partially converted into an anaerobic filter), an upflow vertical-flow constructed wetland (VFCW), a disinfection tank, and a soak pit. | To evaluate the effectiveness of the VFCW positioned between a septic tank and a soak pit, with the aim of reducing groundwater contamination. | The VFCW is used as a secondary treatment unit, downstream of the septic tank anaerobic filter and upstream of the disinfection tank and soak pit. | Aeration pipes installed at the base for passive ventilation. | Reuse and infiltration | 1 |
| Chand et al., 2021 | The treatment units comprised downflow vertical subsurface-flow constructed wetlands (VSSFCWs) operated in three configurations: SB (with biochar and without plants), SBP (with biochar and Typha plants), and SP (with Typha plants and without biochar). The units were operated in tidal-flow mode to promote alternating aerobic and anaerobic conditions. | To investigate nutrient and coliform removal in a tidal-flow constructed wetland system, assessing the effects of biochar (BC) addition and the presence of Typha sp., with effluent recirculation. | Used as a stand-alone treatment system (not integrated with other treatment units) at mesocosm scale. | Biochar was used to increase the sorption area, oxygen retention, and microbial activity; Typha sp. was used to support oxygenation and effluent recirculation. | Reuse in remote and rural areas | – |
| Li et al., 2023 | A laboratory-scale downflow vertical constructed wetland composed of three substrate layers. Magnets were installed to apply a static magnetic field to the system. | To evaluate system performance under a moderate static magnetic field (150 mT) and investigate how this modification influences microbial community structure and key enzyme activity. | The wetland was used as a single stand-alone unit at laboratory scale. | Application of a moderate static magnetic field and intermittent aeration. | – | 0.0023 |
| Pillai and Nair, 2021 | The study used downflow vertical subsurface-flow constructed wetlands (VFCWs) planted with native grass species from India for municipal wastewater treatment and biomass production. The VFCW units were planted with three grass species. | To evaluate municipal wastewater treatment efficiency and biomass production. | Operates as a stand-alone unit for treatment of the residual load. | – | – | 0.03 |
| Sun et al., 2021 | The system comprised three modified downflow vertical-flow constructed wetlands (VFCW-A, VFCW-B, and VFCW-C), each differing in substrate and biological treatment. | To investigate the effects of biochar and activated carbon in the substrate and the use of denitrifying bacteria to improve treatment efficiency. | The wetland was used as a single stand-alone unit at laboratory scale. | Biochar and activated carbon were used as modifying media, together with the addition of denitrifying bacteria. | Discharge to receiving water bodies | 0.12 |
| Wu et al., 2015 | The studied system comprised two intermittently aerated downflow vertical-flow constructed wetlands. The final system was implemented as a laboratory-scale microcosm. | To evaluate the removal of organic pollutants and nitrogen in intermittently aerated vertical-flow constructed wetlands (vfCWs) for decentralized domestic wastewater treatment. | The wetland was used as a single stand-alone unit at laboratory scale. | Intermittent aeration and emergent macrophytes. | – | 0.0204 |
| Fan et al., 2013 | Vertical-flow constructed wetlands (VFCWs) operated as microcosms, using a gravel substrate gradient and the emergent macrophyte Phragmites australis. | To analyze the effects of different influent COD/N ratios and intermittent aeration on nitrogen and organic matter removal from simulated wastewater, with the aim of optimizing nitrification and denitrification processes in the constructed wetlands. | The wetland was used as a single stand-alone unit at laboratory scale. | Intermittent aeration and emergent macrophytes. | – | 0.0066 |
| Chang et al., 2012 | An integrated vertical-flow constructed wetland (IVCW) composed of downflow and upflow chambers. Two plant assemblages were used in two pilot units: Typha orientalis and Arundo donax (Plot 1), and Canna indica and Pontederia cordata (Plot 2). | To evaluate the capacity and performance of constructed wetlands for treating domestic wastewater under high hydraulic loading rates. | Pilot-scale system used as a single stand-alone treatment unit (not integrated with other systems). | Downflow and upflow chambers. | – | 0.5 |
| Zurita et al., 2009 | Pilot-scale constructed wetlands for domestic wastewater treatment, comprising two downflow vertical-flow cells operated with intermittent flooding and drainage cycles. | To evaluate pollutant removal efficiency and the development of ornamental plants under different vertical-flow patterns for domestic wastewater. | Part of a pilot-scale system operated in conjunction with a storage tank. | Intermittent flooding and drainage cycles. | – | 0.128 |
| Rodrigues et al., 2022 | A field-scale experimental modified French vertical-flow constructed wetland (WV-SF), with a surface area of 3.10 m², filled with biochar and planted with Eucalyptus benthamii, treating raw domestic wastewater. | To identify the potential of biochar as a filter medium in a vertical-flow constructed wetland treating raw domestic wastewater. | Experimental field-scale module installed at a wastewater treatment plant and directly fed with raw domestic wastewater. | Biochar as filter medium; intermittent feeding through 12 pulses d⁻¹; alternating cycles of 3.5 days of feeding and 3.5 days of rest. | – | 1.14 |
| Santos et al., 2018 | A 7.5-m² partially saturated vertical-flow constructed wetland, 0.75 m deep, with the lower 0.40 m maintained saturated, filled with coarse sand and planted with Typha domingensis, treating septic-tank effluent. | To evaluate the treatment efficiency of a partially saturated vertical-flow constructed wetland during its first 21 months of operation. | Used as a secondary treatment unit downstream of a septic tank receiving sanitary wastewater from a municipal sewer network. | A 0.40-m saturated bottom zone; intermittent feeding through four pulses d⁻¹; alternating cycles of 3.5 days of feeding and 3.5 days of rest. | – | 0.60 |
| Sezerino et al., 2023 | A long-term evaluation of a 7.5-m² partially saturated vertical-flow constructed wetland with a 0.75-m-deep filter bed, comprising upper and lower gravel layers and a main sand layer, planted with Typha domingensis. Wastewater quality, oxygen consumption, hydraulic behavior, and bacterial dynamics were assessed over six years. | To provide a long-term evaluation of a partially saturated vertical-flow constructed wetland operated as a secondary/advanced treatment unit for urban wastewater under subtropical climate conditions. | Pilot treatment plant comprising a 3-m³ septic tank as primary treatment followed by the wetland as a secondary/advanced treatment unit, designed for the urban wastewater generated by five population equivalents. | The lower 0.40 m of the filter bed, corresponding to 53% of its total depth, was maintained saturated; intermittent feeding through three or four pulses per feeding day; alternating cycles of 3.5 days of feeding and 3.5 days of rest; specific hydraulic loading of approximately 4 L m⁻² min⁻¹; macrophyte pruning every three months. | – | 0.56 |
| Chen et al., 2022 | Horizontal subsurface-flow constructed wetlands (HSSFCWs) divided into groups according to plant species (Iris sibirica and Canna indica). Each unit comprised an inlet chamber, a distribution chamber, a filtration chamber, and an outlet chamber. | To evaluate the combined effects of aeration and plant species on the purification efficiency of simulated domestic effluent. | Pilot-scale system used as a single stand-alone treatment unit (not integrated with other systems). | Intermittent aeration applied at different positions in the system and a combination of plant species. | – | – |
| Angassa et al., 2019 | A pilot-scale horizontal subsurface-flow constructed wetland (HSSFCW), with two modules arranged in parallel and a sewage collection tank. | To evaluate the potential of Phragmites karka for phytoremediation and biomass production while treating municipal wastewater for the removal of COD, total nitrogen (TN), and total phosphorus (TP). | Pilot-scale system used as a single stand-alone treatment unit (not integrated with other systems). | Passive aerators (piping) were used to increase oxygenation through the roots of Phragmites karka. | Discharge to receiving water bodies | – |
| Wang; Li, 2017 | Two constructed wetlands were used: one preceded by an aerated infiltration device (CW1) and another without aeration (CW2), allowing comparison of the influence of pre-aeration on nitrogen removal performance. | To investigate the effects of seasonal pre-aeration on microbial performance and nitrogen removal in constructed wetlands. | Pilot-scale system operated in a controlled laboratory environment. | Pre-aeration to enhance nitrogen removal under aerobic and anaerobic conditions. | – | – |
| Mburu et al., 2013 | A pilot-scale horizontal subsurface-flow constructed wetland (HSSF-CW) integrated into a waste stabilization pond (WSP) system. The WSP system included primary and secondary facultative ponds followed by a maturation pond. | To compare pollutant removal efficiency, land requirements, and investment, operation, and maintenance costs between the waste stabilization pond system (WSP) and the HSSF-CW for domestic sewage treatment. | Pilot-scale system located downstream of primary treatment at the wastewater treatment plant and upstream of tertiary treatment. | – | Discharge to receiving water bodies | 3 |
| Zurita et al., 2009 | A pilot-scale horizontal wetland configured to treat domestic sewage under continuous horizontal subsurface flow. Ornamental plants were cultivated in the HFCW to evaluate both treatment efficiency and the potential for commercial flower production. | To evaluate pollutant removal efficiency and the development of ornamental plants in constructed wetlands treating domestic sewage. | A stand-alone unit of the pilot-scale treatment system. | – | – | 0.128 |
| Ho et al., 2020 | Horizontal subsurface-flow constructed wetlands (HSSF-CWs) planted with Brachiaria mutica. The system comprised two configurations: one planted and one unplanted. | To evaluate domestic wastewater treatment efficiency using an HSSF-CW planted with Brachiaria mutica. | A single stand-alone pilot-scale treatment unit, without integration with additional systems. | Gravel and sand layers promoted filtration and sediment retention, while Brachiaria mutica contributed to nutrient uptake. | Discharge to receiving water bodies | 0.0288 |
| Ergaieg et al., 2021 | Horizontal subsurface-flow constructed wetlands designed for tertiary treatment of municipal wastewater. The system was implemented in conjunction with a maturation pond to complement sewage treatment. | To monitor full-scale HSF-CW performance for tertiary treatment and investigate the use of electrical resistivity to detect malfunctions such as clogging. | Full-scale system used in conjunction with a maturation pond. The wetland is positioned upstream of the pond in the complete treatment train. | – | Reuse | 242 |
| Ergaieg et al., 2021 | HSF-CW2 is a horizontal subsurface-flow constructed wetland designed for tertiary treatment of municipal wastewater. It operates in conjunction with HSF-CW1 and a maturation pond, forming a complete system for sewage polishing and reuse. | To monitor full-scale HSF-CW2 performance for tertiary treatment and treated-water reuse. The electrical resistivity method was also used to detect anomalies such as clogging. | Full-scale system used in conjunction with a maturation pond. The wetland is positioned upstream of the pond in the complete treatment train. | – | Reuse | 121 |
| Sousa et al., 2024 | A decentralized wastewater treatment system installed at a rural inn in Tubarão, Santa Catarina, comprising an 8.2-m³ septic tank followed by a 72-m² horizontal-flow constructed wetland with a depth of 0.70 m, filled with coarse sand, clay, and rice husk and planted with Zizanopsis bonariensis. | To evaluate the quality and reuse potential of effluents treated by constructed wetland systems used for decentralized wastewater treatment in urban and rural settings in Southern Brazil. | The HFCW was used as a secondary treatment unit downstream of a septic tank and received regular domestic wastewater generated at a rural inn designed for 150 population equivalents. | Horizontal flow through a filter bed composed of coarse sand, clay, and rice husk and planted with Zizanopsis bonariensis. | Soil infiltration; potential use for limited fertigation. | – |
| Silva et al., 2017 | A pilot-scale horizontal subsurface-flow constructed wetland installed at the São Paulo State University campus in Bauru, Brazil, treating low-strength domestic wastewater generated mainly from toilets and lavatories. The 40.5-m² rectangular bed was filled with sand, gravel, Styrofoam beads, and crushed rock and planted with 90 seedlings of Vetiver grass (Chrysopogon zizanioides). | To evaluate and compare the performance of a pilot-scale horizontal subsurface-flow constructed wetland and an anaerobic/aerobic baffled reactor operated in parallel with the same low-strength domestic wastewater. | Secondary treatment unit preceded by screening, settling, equalization, and storage tanks. The HFCW was operated independently and in parallel with the AABR, was designed to serve 20 inhabitants, and was monitored for 63 days. | Horizontal subsurface flow through a layered filter bed; 1% longitudinal slope; 88-h hydraulic retention time; perforated PVC inlet and outlet pipes used to distribute the wastewater throughout the support medium. | – | 2.76 |
| Martel-Rodríguez et al., 2022 | The treatment system consisted of a hybrid constructed wetland with two downflow vertical-flow units (VFCWs) and one horizontal-flow unit (HFCW). Wastewater was directed to the VFCWs and subsequently to the HFCW before being stored in a pond. | To evaluate the long-term efficiency of a hybrid constructed wetland system for urban wastewater treatment. | Full-scale system initially designed to serve a population equivalent of 100, although it currently treats higher flow rates and loads due to additional community demands. | The VFCWs alternate loading and resting periods to improve pollutant removal. Typha latifolia vegetation is used to improve effluent quality, and the system includes structures that facilitate oxygen transfer. | Irrigation and soil infiltration | 30.25 |
| Zhai et al., 2016 | The studied system was a full-scale hybrid wetland comprising two stages: a downflow vertical-baffle-flow wetland (VBFW) as the first stage and a horizontal subsurface-flow wetland (HSFW) as the second stage. | To investigate nitrogen removal pathways under summer conditions in a hybrid constructed wetland and assess microbial contributions to nitrogen removal processes. | The hybrid constructed wetland operates at full scale. It was implemented at the Qinghe Agro-Park wastewater treatment plant, serving a community of approximately 6,000 people and providing a treatment capacity of 500-600 m³ d⁻¹. | Subsurface flow with natural aeration and rhizospheric oxygen transfer by plants. | Reuse | 500-600 |
| Marzec et al., 2024 | The studied treatment system was a hybrid wastewater treatment plant combining vertical-flow (VF) and horizontal-flow (HF) beds, both configured as soil-plant beds with an aeration system. The facility operates at full scale and treats domestic sewage from a school, with a design capacity of 4.5 m³ d⁻¹. The system includes a three-chamber septic tank, a pumping station equipped with an aeration system, and planted beds containing giant miscanthus and willow. | To analyze the efficiency and reliability of total suspended solids (TSS), biochemical oxygen demand (BOD5), and chemical oxygen demand (COD) removal, as well as the efficiency of the aeration system and energy intensity. | Full-scale system treating domestic sewage from a school, with a design capacity of 4.5 m³ d⁻¹. | Active aeration was applied in the system, specifically at the pumping station, where the effluent was oxygenated before being transferred to the VF bed. | Discharged to soil | 4.5 |
| Ye and Li, 2009 | The system, termed the Towery Hybrid Constructed Wetland (THCW), was designed to treat domestic sewage in rural communities. It comprised three stages: stages 1 and 3 were rectangular horizontal subsurface-flow constructed wetlands (HSSFCWs), whereas stage 2 was a free-water-surface wetland arranged in three cascading layers to enhance oxygenation. | To evaluate nitrogen removal and THCW performance under high hydraulic loading conditions, exploring passive oxygenation to increase the nitrification rate and enhance coupling between nitrification and denitrification processes. | Pilot-scale system designed to serve the equivalent of 40 inhabitants. | Cascades were used for passive oxygenation, specific plants were used for nutrient removal, and passive aeration was promoted by cascading flow in the second stage. | Discharge to receiving water bodies | – |
| Micek et al., 2020 | The system comprised an initial three-chamber settling tank followed by two hybrid vertical-flow (VF) and horizontal-flow (HF) constructed wetland beds. The VF bed was planted with common reed (Phragmites australis), and the HF bed was planted with willow (Salix viminalis). The beds were filled with coarse sand and protected by an HDPE layer to prevent soil contamination. The system treated 0.4 m³ d⁻¹ over a total area of 48 m². | To evaluate the reliability and efficiency of pollutant removal in two hybrid constructed wetland systems in Roztocze National Park, Poland. | Full-scale system implemented to treat domestic wastewater under real operating conditions in Roztocze National Park, Poland. | The system used a hybrid vertical- and horizontal-bed configuration (VF-HF), promoting alternating aerobic and anaerobic conditions. | Routed to an infiltration pond | 0.4 |
| Micek et al., 2020 | The system comprised an initial three-chamber settling tank followed by two hybrid vertical-flow (VF) and horizontal-flow (HF) constructed wetland beds. The VF bed was planted with common reed (Phragmites australis), and the HF bed was planted with willow (Salix viminalis). The system was designed to treat 1.0 m³ d⁻¹ of domestic effluent. | To evaluate the reliability and efficiency of pollutant removal in two hybrid constructed wetland systems in Roztocze National Park, Poland. | Full-scale system implemented to treat domestic wastewater under real operating conditions in Roztocze National Park, Poland. | The system used a hybrid vertical- and horizontal-bed configuration (VF-HF), promoting alternating aerobic and anaerobic conditions. | Routed to an infiltration pond | 1 |
| Marzec et al., 2019 | A hybrid constructed wetland (VF-HF) with vertical- and horizontal-flow beds operated in series. It was designed to treat domestic sewage and included a pretreatment unit followed by two biological treatment stages. | To evaluate system efficiency and reliability in the removal of organic pollutants and nutrients. | Operating full-scale on-site treatment plant serving a multi-family building; planned capacity of 2.5 m³ d⁻¹ and mean wastewater flow of 1.2 m³ d⁻¹ during the study. | The system used a hybrid vertical- and horizontal-bed configuration (VF-HF), promoting alternating aerobic and anaerobic conditions. | Discharged into the Urzędówka River | 1.2 |
| Gajewska et al., 2018 | The treatment system was a hybrid wetland comprising a horizontal-flow bed followed by a vertical-flow bed (HF+VF), with both beds operating under subsurface-flow conditions. | To evaluate the removal of organic pollutants and nutrients by comparing the treatment efficiency of the HF+VF configuration. | Full-scale system used to treat domestic sewage from a residence. | The system used a hybrid horizontal- and vertical-bed configuration (HF-VF), promoting alternating aerobic and anaerobic conditions. | – | 0.3 |
| Gajewska et al., 2018 | The system was a hybrid wetland comprising one vertical-flow unit followed by one horizontal-flow unit (VF+HF), operated in series for domestic sewage treatment. | To evaluate the removal of organic pollutants and nutrients by comparing the treatment efficiency of the VF+HF configuration. | Full-scale system used to treat domestic sewage from a residence. | The system used a hybrid vertical- and horizontal-bed configuration (VF-HF), promoting alternating aerobic and anaerobic conditions. | – | 0.3 |
| Silva; Bueno, 2015 | A pilot-scale hybrid constructed wetland comprising a vertical-flow unit followed by a horizontal-flow unit, built as a 6.0 × 1.2 × 0.6 m basin divided into two 3.0-m-long sections, with a total surface area of 7.2 m². The system was filled with graded crushed stone and planted with five emergent macrophyte species at approximately four seedlings m⁻². | To evaluate the applicability of a pilot-scale hybrid constructed wetland for sanitary wastewater treatment. | The system was installed at the University of São Paulo and received sanitary wastewater generated at the university residential complex and central restaurant after preliminary treatment by mechanical screening, grit removal, and flow measurement through a Parshall flume. | The VFCW was intermittently fed through eight applications d⁻¹, with 25 L per application, a duration of 5 min, and intervals of 3 h. The VFCW effluent was pumped continuously to the HFCW, which remained saturated. Before the experimental phase, the system underwent a 14-week acclimation period. | – | 0.20 |
| Freitas, 2017 | A full-scale experimental hybrid constructed wetland installed at the Federal University of Santa Catarina, comprising a descending vertical-flow constructed wetland followed by a horizontal-flow constructed wetland. The units were filled with coarse sand and gravel and planted with Typha domingensis. The performance dataset covered 19 months, from operational week 39 to week 117. | To evaluate the hydrodynamics and treatment performance of the horizontal-flow constructed wetland component of a hybrid system used for sanitary wastewater treatment. | Secondary/advanced treatment system preceded by a decant-digester and designed to treat sanitary wastewater equivalent to five inhabitants. The DVCW measured 3.30 × 2.30 m, with a useful depth of 0.70 m, whereas the HFCW measured 5.30 × 3.00 m, with a useful depth of 0.60 m and a saturated depth of 0.50 m. | Intermittent feeding of the DVCW through three or four 192-L pulses per feeding day, with alternating periods of 3.5 days of feeding and 3.5 days of rest; continuous feeding of the HFCW with the DVCW effluent; maintenance of saturated conditions in the HFCW. | – | 0.576–0.768 |
| Rousso, 2017 | A full-scale hybrid constructed wetland installed at the Federal University of Santa Catarina, comprising a 7.60-m² descending vertical-flow constructed wetland followed by a 16.00-m² horizontal-flow constructed wetland. Both units were filled predominantly with coarse sand and gravel and planted with Typha domingensis. | To evaluate pollutant-removal performance and hydraulic behavior, including outlet-flow patterns and evapotranspiration, of a hybrid constructed wetland during its initial operating period. | The treatment train comprised a septic tank as primary treatment, followed by the VFCW and HFCW operated in series. The system treated sanitary wastewater in a volume equivalent to that generated by a Brazilian single-family household. | Intermittent VFCW feeding through three or four pulses per feeding day, with 3-h intervals between pulses; alternating periods of 3.5 days of feeding and 3.5 days of rest; free drainage in the VFCW followed by continuous subsurface horizontal flow through the saturated HFCW. | – | 0.447–0.596 |

**Influent and effluent concentrations and removal performance — Tables S3 to S11.**

Tables S3–S11 present the system-level influent and effluent concentrations and the corresponding removal efficiencies for biochemical oxygen demand (BOD), chemical oxygen demand (COD), total suspended solids (TSS), total nitrogen (TN), and total phosphorus (TP). Descriptive statistics were calculated separately for each configuration and parameter. Because not all studies reported all variables, the number of available systems varied among parameters and is reported as n in each descriptive table.

Table S3: Mean influent concentrations of biochemical oxygen demand (BOD), chemical oxygen demand (COD), total suspended solids (TSS), total nitrogen (TN), and total phosphorus (TP) in the VFCW, HFCW, and HCW systems included in the analysis.

| Author | CW configuration | TSS  (mg L⁻¹) | BOD  (mg O₂ L⁻¹) | COD  (mg O₂ L⁻¹) | TN  (mg L⁻¹) | TP  (mg L⁻¹) |
| --- | --- | --- | --- | --- | --- | --- |
| Suresh et al., 2023 | VFCW | 69.5 | 49.6 | 248.1 | – | – |
| Chand et al., 2021 |  | – | 164.1 | 360.1 | 59.6 | – |
| Li et al., 2023 |  | – | – | 201.0 | 39.9 | 5.2 |
| Pillai and Nair, 2021 |  | 279.0 | 180.0 | 412.0 | 52.6 | – |
| Sun et al., 2021 |  | – | – | 51.1 | 13.6 | 0.6 |
| Wu et al., 2015 |  | – | – | 435.7 | 44.0 | 3.9 |
| Fan et al., 2013 |  | – | – | 429.3 | 44.7 | 3.9 |
| Chang et al., 2012 |  | – | – | 288.7 | 30.7 | 3.1 |
| Zurita et al., 2009 |  | 57.5 | 115.5 | 247.5 | 28.7 | 8.3 |
| Rodrigues et al., 2022 |  | 346.5 | 171.1 | 350.0 | – | 3.8 |
| Santos et al., 2018 |  | 45.7 | – | 496.0 | 80.4 | – |
| Sezerino et al., 2023 |  | 82.0 | 270.0 | 440.0 | 78.0 | – |
| Chen et al., 2022 | HFCW | – | – | 272.0 | 49.8 | 2.9 |
| Angassa et al., 2019 |  | – | – | 470.0 | 84.0 | 41.0 |
| Wang; Li, 2017 |  | – | 35.4 | 102.1 | 29.2 | – |
| Mburu et al., 2013 |  | 118.0 | 232.0 | 424.0 | – | 4.0 |
| Zurita et al., 2009 |  | 57.5 | 115.5 | 247.5 | 28.7 | 8.3 |
| Ho et al., 2020 |  | 36.6 | 94.8 | 163.7 | 56.1 | 14.3 |
| Ergaieg et al., 2021 |  | 54.8 | 43.2 | 123.2 | – | – |
| Ergaieg et al., 2021 |  | 54.8 | 43.2 | 123.2 | – | – |
| Sousa et al., 2024 |  | 451 | 326 | 676 | – | – |
| Silva et al., 2017 |  | 48.00 | 85.00 | 214.00 | – | – |
| Martel-Rodríguez et al., 2022 | HCW | 158 | 432.00 | 662 | 91 | – |
| Zhai et al., 2016 |  | – | – | 72.1 | 35.2 | 2.4 |
| Marzec et al., 2024 |  | 65.74 | 101.25 | 256.73 | – | – |
| Ye and Li, 2009 |  | 124.20 | – | 320.20 | 69.38 | 6.22 |
| Micek et al., 2020 |  | 129.00 | 275.00 | 785.00 | 144.00 | 27.20 |
| Micek et al., 2020 |  | 70.60 | 123.00 | 390.00 | 98.60 | 19.40 |
| Marzec et al., 2019 |  | 297.00 | 537.00 | 1309.00 | 157.00 | 21.00 |
| Gajewska et al., 2018 |  | – | 168.70 | 408.10 | 134.30 | – |
| Gajewska et al., 2018 |  | – | 168.70 | 408.10 | 134.30 | – |
| Silva; Bueno, 2015 |  | 2980.00 | – | 664.00 | – | 5.50 |
| Freitas, 2017 |  | 59.7 | 261.00 | 440.30 | 83.20 | – |
| Rousso, 2017 |  | 44.78 | – | 586.00 | 76.41 | – |

Table S4: Descriptive statistics for influent BOD, COD, TSS, TN, and TP concentrations in the VFCW and HFCW systems. Sample size varied among parameters according to data availability.

| Parameter | VFCW | | | | | HFCW | | | | | |
| --- | --- | --- | --- | --- | --- | --- | --- | --- | --- | --- | --- |
|  | BOD (mg O₂/L) | COD (mg O₂/L) | TSS (mg/L) | TN (mg/L) | TP (mg/L) | BOD (mg O₂/L) | COD (mg O₂/L) | TSS (mg/L) | TN (mg/L) | TP (mg/L) |  |
| n | 6 | 12 | 6 | 10 | 7 | 8 | 10 | 7 | 5 | 5 |  |
| Mean | 158.38 | 329.96 | 146.70 | 47.22 | 4.11 | 121.89 | 281.56 | 117.26 | 49.56 | 14.10 |  |
| SD | 73.20 | 127.34 | 130.94 | 21.21 | 2.32 | 104.05 | 186.63 | 149.47 | 22.80 | 15.69 |  |
| Median | 167.60 | 355.05 | 75.75 | 44.35 | 3.90 | 89.91 | 230.75 | 54.80 | 49.80 | 8.30 |  |
| Maximum | 270.00 | 496.00 | 346.50 | 80.40 | 8.30 | 326.00 | 676.00 | 451.00 | 84.00 | 41.00 |  |
| Minimum | 49.60 | 51.10 | 45.70 | 13.60 | 0.60 | 35.37 | 102.05 | 36.63 | 28.70 | 2.90 |  |

Table S5: Descriptive statistics for influent BOD, COD, TSS, TN, and TP concentrations in the HCW systems. Sample size varied among parameters according to data availability.

| Parameter | BOD (mg O₂/L) | COD (mg O₂/L) | TSS (mg/L) | TN (mg/L) | TP (mg/L) |
| --- | --- | --- | --- | --- | --- |
| n | 8 | 12 | 9 | 10 | 6 |
| Mean | 258 | 525 | 437 | 102 | 14 |
| SD | 155 | 316 | 957 | 39 | 10 |
| Median | 215 | 424 | 124 | 95 | 13 |
| Maximum | 537 | 1309 | 2980 | 157 | 27 |
| Minimum | 101 | 72 | 45 | 35 | 2 |

Table S6: Mean effluent concentrations and corresponding removal efficiencies for BOD, COD, TSS, TN, and TP in the VFCW systems.

| **AUTHOR** | **BOD** | | **COD** | | **TSS** | | **TN** | | **TP** | |
| --- | --- | --- | --- | --- | --- | --- | --- | --- | --- | --- |
|  | **mg O₂/L** | **%** | **mg O₂/L** | **%** | **mg/L** | **%** | **mg/L** | **%** | **mg/L** | **%** |
| Suresh et al., 2023 | 12.12 | 76 | 55.77 | 78 | 8.37 | 88 | - | - | - | - |
| Chand et al., 2021 | 6.5 | 96 | 18.83 | 95 | - | - | 20.72 | 65 | - | - |
| Li et al., 2023 | - | - | 14.91 | 93 | - | - | 10.96 | 73 | 0.22 | 96 |
| Pillai and Nair, 2021 | 18.2 | 90 | 90.23 | 78 | 28.37 | 90 | 29.06 | 45 | - | - |
| Sun et al., 2021 | - | - | 3.91 | 92 | - | - | 2.07 | 85 | 0.09 | 84 |
| Wu et al., 2015 | - | - | 6.57 | 98 | - | - | 4.69 | 89 | 0.34 | 91 |
| Fan et al., 2013 | - | - | 16.84 | 96 | - | - | 4.72 | 89 | 0.33 | 92 |
| Chang et al., 2012 | - | - | 103 | 64 | - | - | 26.4 | 14 | 1.51 | 50 |
| Zurita et al., 2009 | 18.5 | 84 | 41.3 | 83 | 16.9 | 71 | 14.1 | 51 | 3.8 | 54 |
| Rodrigues et al., 2022 | 20.6 | 88 | 73.8 | 79 | 12.9 | 96 | - | - | 1.5 | 61 |
| Santos et al., 2018 | - | - | 45.7 | 91 | 2.3 | 95 | 44.3 | 45 | - | - |
| Sezerino et al., 2023 | 25 | 91 | 34 | 92 | 1 | 99 | 47 | 40 | - | - |

Table S7: Descriptive statistics for effluent BOD, COD, TSS, TN, and TP concentrations and their corresponding removal efficiencies in the VFCW systems.

| **Parameter** | **BOD** | | **COD** | | **TSS** | | **TN** | | **TP** | |
| --- | --- | --- | --- | --- | --- | --- | --- | --- | --- | --- |
|  | mg O₂/L | % | mg O₂/L | % | mg/L | % | mg/L | % | mg/L | % |
| n | 6 | 6 | 12 | 12 | 6 | 6 | 10 | 10 | 7 | 7 |
| Mean | 17 | 87 | 42 | 87 | 12 | 90 | 20 | 60 | 1.1 | 75 |
| SD | 7 | 7 | 33 | 10 | 10 | 10 | 16 | 25 | 1.3 | 20 |
| Median | 18 | 89 | 38 | 92 | 11 | 92 | 17 | 58 | 0.3 | 84 |
| Maximum | 25 | 96 | 103 | 98 | 28 | 99 | 47 | 89 | 3.8 | 96 |
| Minimum | 7 | 76 | 4 | 64 | 1 | 71 | 2 | 14 | 0.09 | 50 |

Table S8: Mean effluent concentrations and corresponding removal efficiencies for BOD, COD, TSS, TN, and TP in the HFCW systems.

| **AUTHOR** | **BOD** | | **COD** | | **TSS** | | **TN** | | **TP** | |
| --- | --- | --- | --- | --- | --- | --- | --- | --- | --- | --- |
|  | **mg O₂/L** | **%** | **mg O₂/L** | **%** | **mg/L** | **%** | **mg/L** | **%** | **mg/L** | **%** |
| Chen et al., 2022 | - | - | 59.8 | 78 | - | - | 22 | 56 | 0.12 | 96 |
| Angassa et al., 2019 | - | - | 30 | 94 | - | - | 2.3 | 97 | 4.3 | 90 |
| Wang; Li, 2017 | 3.59 | 90 | 42.65 | 58 | - | - | 21.34 | 27 | - | - |
| Mburu et al., 2013 | 29 | 88 | 58 | 86 | 19 | 84 | - | - | 3 | 25 |
| Zurita et al., 2009 | 21.2 | 82 | 51.3 | 79 | 8.5 | 85 | 13.8 | 52 | 4.9 | 41 |
| Ho et al., 2020 | 7.1 | 93 | 20.83 | 87 | 18.23 | 50 | 18.3 | 67 | 1.78 | 88 |
| Ergaieg et al., 2021 | 12.26 | 72 | 47.79 | 61 | 15.99 | 71 | - | - | - | - |
| Ergaieg et al., 2021 | 14.77 | 66 | 50.74 | 59 | 19.53 | 64 | - | - | - | - |
| Sousa et al., 2024 | 27 | 92 | 76 | 89 | 82 | 82 | - | - | - | - |
| Silva et al., 2017 | 38 | 55 | 47 | 78 | 10 | 79 | - | - | - | - |

Table S9: Descriptive statistics for effluent BOD, COD, TSS, TN, and TP concentrations and their corresponding removal efficiencies in the HFCW systems.

| **PARAMETER** | **BOD** | | **COD** | | **TSS** | | **TN** | | **TP** | |
| --- | --- | --- | --- | --- | --- | --- | --- | --- | --- | --- |
|  | **mg O₂/L** | **%** | **mg O₂/L** | **%** | **mg/L** | **%** | **mg/L** | **%** | **mg/L** | **%** |
| n | 8 | 8 | 10 | 10 | 7 | 7 | 5 | 5 | 5 | 5 |
| MEAN | 19 | 79 | 48 | 77 | 25 | 74 | 16 | 60 | 2.8 | 68 |
| SD | 12 | 14 | 15 | 13 | 26 | 13 | 8 | 26 | 1.9 | 32 |
| MEDIAN | 18 | 85 | 49 | 79 | 18 | 79 | 18 | 56 | 3.0 | 88 |
| MAXIMUM | 38 | 93 | 76 | 94 | 82 | 85 | 22 | 97 | 4.9 | 96 |
| MINIMUM | 4 | 55 | 21 | 58 | 9 | 50 | 2 | 27 | 0.12 | 25 |

Table S10: Mean effluent concentrations and corresponding removal efficiencies for BOD, COD, TSS, TN, and TP in the HCW systems.

| **AUTHOR** | **CW** | **BOD** | | **COD** | | **TSS** | | **TN** | | **TP** | |
| --- | --- | --- | --- | --- | --- | --- | --- | --- | --- | --- | --- |
|  |  | **mg O₂/L** | **%** | **mg O₂/L** | **%** | **mg/L** | **%** | **mg/L** | **%** | **mg/L** | **%** |
| Martel-Rodríguez et al., 2022 | VF + HF | 60.00 | 86 | 124.00 | 81 | 14.00 | 91 | 56.00 | 38 | - | - |
| Zhai et al., 2016 | VF + HF | - | - | 35.5 | 51 | - | - | 12.20 | 65 | 0.90 | 63 |
| Marzec et al., 2024 | VF + HF | 1.96 | 98 | 26.46 | 90 | 12.08 | 82 | - | - | - | - |
| Ye and Li, 2009 | FWS + HF | - | - | 49.3 | 85 | 14.2 | 89 | 11.9 | 83 | 2.23 | 64 |
| Micek et al., 2020 | VF + HF | 3.5 | 99 | 34.8 | 96 | 26.7 | 79 | 39.9 | 72 | 2.6 | 90 |
| Micek et al., 2020 | VF + HF | 3.00 | 98 | 15.2 | 96 | 9.9 | 86 | 13.8 | 86 | 1.1 | 94 |
| Marzec et al., 2019 | VF + HF | 6.60 | 99 | 31.8 | 98 | 18 | 94 | 56.4 | 64 | 6.7 | 68 |
| Gajewska et al., 2018 | HF + VF | 12.20 | 93 | 46.4 | 89 | - | - | 70.8 | 47 | - | - |
| Gajewska et al., 2018 | VF + HF | 11.70 | 93 | 40.4 | 90 | - | - | 54.3 | 60 | - | - |
| Silva and Bueno, 2015 | VF + HF | - | - | 93 | 86 | 12 | 100 | - | - | 1.8 | 67 |
| Freitas, 2017 | VF + HF | 26.00 | 90 | 24.3 | 94 | 2.4 | 96 | 20.2 | 76 | - | - |
| Rousso, 2017 | VF + HF | - | - | 12 | 98 | 0.04 | 100 | 24.81 | 68 | - | - |

Table S11: Descriptive statistics for effluent BOD, COD, TSS, TN, and TP concentrations and their corresponding removal efficiencies in the HCW systems.

| **PARAMETER** | **BOD** | | **COD** | | **TSS** | | **TN** | | **TP** | |
| --- | --- | --- | --- | --- | --- | --- | --- | --- | --- | --- |
|  | **mg O₂/L** | **%** | **mg O₂/L** | **%** | **mg/L** | **%** | **mg/L** | **%** | **mg/L** | **%** |
| n | 8 | 8 | 12 | 12 | 9 | 9 | 10 | 10 | 6 | 6 |
| MEAN | 16 | 94 | 44 | 88 | 12 | 91 | 36 | 66 | 2.6 | 74 |
| SD | 20 | 5 | 33 | 13 | 8 | 7 | 22 | 15 | 2.1 | 14 |
| MEDIAN | 9 | 95 | 35 | 90 | 12 | 91 | 32 | 66 | 2.0 | 68 |
| MAXIMUM | 60 | 99 | 124 | 98 | 27 | 100 | 71 | 86 | 6.7 | 94 |
| MINIMUM | 2 | 86 | 12 | 51 | 0.04 | 79 | 12 | 38 | 0.9 | 63 |

**Reliability assessment and regulatory benchmark scenarios** **— Tables S12 to S20.**

Reliability was evaluated at a target non-exceedance probability of 95%. Two comparative discharge scenarios were considered. The stringent benchmark adopted limits of 60 mg O₂ L⁻¹ for BOD, 120 mg O₂ L⁻¹ for COD, 100 mg L⁻¹ for TSS, 10 mg L⁻¹ for TN, and 1 mg L⁻¹ for TP. The less stringent benchmark adopted limits of 90 mg O₂ L⁻¹ for BOD, 200 mg O₂ L⁻¹ for COD, 150 mg L⁻¹ for TSS, 20 mg L⁻¹ for TN, and 4 mg L⁻¹ for TP. Values of x̄/mₓ ≤ 1 indicate that the observed mean concentration met the allowable mean target at the specified reliability level, whereas values > 1 indicate that the target was not achieved.

Table S12: Standard normal deviate (Zs) and corresponding non-exceedance probability (1 − α) for BOD, COD, TSS, TN, and TP in the VFCW systems relative to the most stringent state discharge limits adopted in the analysis.

| **AUTHOR** | **BOD** | | **COD** | | **TSS** | | **TN** | | **TP** | |
| --- | --- | --- | --- | --- | --- | --- | --- | --- | --- | --- |
|  | **Zs** | **1 − α** | **Zs** | **1 − α** | **Zs** | **1 − α** | **Zs** | **1 − α** | **Zs** | **1 − α** |
| Suresh et al., 2023 | 6.40 | 100% | 15.98 | 100% | 9.33 | 100% | — | — | — | — |
| Chand et al., 2021 | 6.17 | 100% | 12.42 | 100% | — | — | -7.71 | 0% | — | — |
| Li et al., 2023 | — | — | 7.91 | 100% | — | — | -1.38 | 8% | 5.03 | 100% |
| Pillai and Nair, 2021 | 2.10 | 98% | 1.21 | 89% | 3.28 | 100% | -6.10 | 0% | — | — |
| Sun et al., 2021 | — | — | 18.84 | 100% | — | — | 10.02 | 100% | 11.08 | 100% |
| Wu et al., 2015 | — | — | 5.61 | 100% | — | — | 1.54 | 94% | 2.06 | 98% |
| Fan et al., 2013 | — | — | 3.16 | 100% | — | — | 1.35 | 91% | 1.60 | 95% |
| Chang et al., 2012 | — | — | 0.56 | 71% | — | — | -6.53 | 0% | -0.35 | 36% |
| Zurita et al., 2009 | 2.15 | 98% | 2.12 | 98% | 2.93 | 100% | -1.64 | 5% | -4.45 | 0% |
| Rodrigues et al., 2022 | 2.61 | 100% | 1.05 | 85% | 2.47 | 99% | — | — | -1.09 | 14% |
| Santos et al., 2018 | — | — | 1.66 | 95% | 3.41 | 100% | -6.44 | 0% | — | — |
| Sezerino et al., 2023 | 3.32 | 100% | 1.96 | 97% | 4.26 | 100% | -3.59 | 0% | — | — |

Note: The stringent limits were 60 mg O₂ L⁻¹ for BOD, 120 mg O₂ L⁻¹ for COD, 100 mg L⁻¹ for TSS, 10 mg L⁻¹ for TN, and 1 mg L⁻¹ for TP. Probabilities were rounded to the nearest whole percentage.

Table S13: Standard normal deviate (Zs) and corresponding non-exceedance probability (1 − α) for BOD, COD, TSS, TN, and TP in the HFCW systems relative to the most stringent state discharge limits adopted in the analysis.

| **AUTHOR** | **BOD** | | **COD** | | **TSS** | | **TN** | | **TP** | |
| --- | --- | --- | --- | --- | --- | --- | --- | --- | --- | --- |
|  | **Zs** | **1 − α** | **Zs** | **1 − α** | **Zs** | **1 − α** | **Zs** | **1 − α** | **Zs** | **1 − α** |
| Chen et al., 2022 | — | — | 1.73 | 96% | — | — | -1.61 | 5% | 4.19 | 100% |
| Angassa et al., 2019 | — | — | 3.79 | 100% | — | — | 3.47 | 100% | -2.33 | 1% |
| Wang and Li, 2017 | 4.54 | 100% | 2.59 | 100% | — | — | -2.93 | 0% | — | — |
| Mburu et al., 2013 | 2.55 | 99% | 2.62 | 100% | 4.02 | 100% | — | — | -10.96 | 0% |
| Zurita et al., 2009 | 2.38 | 99% | 2.86 | 100% | 3.18 | 100% | -1.39 | 8% | -6.88 | 0% |
| Ho et al., 2020 | 94.73 | 100% | 3.35 | 100% | 5.65 | 100% | -1.64 | 5% | -1.31 | 10% |
| Ergaieg et al., 2021 (system 1) | 5.00 | 100% | 2.78 | 100% | 5.78 | 100% | — | — | — | — |
| Ergaieg et al., 2021 (system 2) | 7.40 | 100% | 3.06 | 100% | 8.26 | 100% | — | — | — | — |
| Sousa et al., 2024 | 1.41 | 92% | 1.08 | 86% | 0.71 | 76% | — | — | — | — |
| Silva et al., 2017 | 1.75 | 96% | 2.41 | 99% | 3.18 | 100% | — | — | — | — |

Note: The stringent limits were 60 mg O₂ L⁻¹ for BOD, 120 mg O₂ L⁻¹ for COD, 100 mg L⁻¹ for TSS, 10 mg L⁻¹ for TN, and 1 mg L⁻¹ for TP. Probabilities were rounded to the nearest whole percentage.

Table S14: Standard normal deviate (Zs) and corresponding non-exceedance probability (1 − α) for BOD, COD, TSS, TN, and TP in the HCW systems relative to the most stringent state discharge limits adopted in the analysis.

| **AUTHOR** | **BOD** | | **COD** | | **TSS** | | **TN** | | **TP** | |
| --- | --- | --- | --- | --- | --- | --- | --- | --- | --- | --- |
|  | **Zs** | **1 − α** | **Zs** | **1 − α** | **Zs** | **1 − α** | **Zs** | **1 − α** | **Zs** | **1 − α** |
| Martel-Rodríguez et al., 2022 | 0.24 | 59% | 0.08 | 53% | 2.26 | 99% | -5.65 | 0% | — | — |
| Zhai et al., 2016 | — | — | 2.91 | 100% | — | — | -0.31 | 38% | 0.48 | 68% |
| Marzec et al., 2024 | 4.73 | 100% | 3.55 | 100% | 2.63 | 100% | — | — | — | — |
| Ye and Li, 2009 | — | — | 3.35 | 100% | 5.32 | 100% | -0.38 | 35% | -1.74 | 4% |
| Micek et al., 2020 (system 1) | 4.89 | 100% | 3.15 | 100% | 2.74 | 100% | -2.58 | 0% | -1.06 | 14% |
| Micek et al., 2020 (system 2) | 5.68 | 100% | 4.99 | 100% | 3.99 | 100% | -0.00 | 50% | -0.00 | 50% |
| Marzec et al., 2019 | 2.65 | 100% | 2.56 | 99% | 2.35 | 99% | -2.18 | 1% | -4.89 | 0% |
| Gajewska et al., 2018 | 2.55 | 99% | 1.81 | 96% | — | — | -4.77 | 0% | — | — |
| Gajewska et al., 2018 | 1.92 | 97% | 1.55 | 94% | — | — | -3.25 | 0% | — | — |
| Silva and Bueno, 2015 | — | — | 0.73 | 77% | 3.90 | 100% | — | — | -2.57 | 1% |
| Freitas, 2017 | 2.26 | 99% | 3.65 | 100% | 3.62 | 100% | -1.12 | 13% | — | — |
| Rousso, 2017 | — | — | 3.78 | 100% | 6.50 | 100% | -1.93 | 3% | — | — |

Note: The stringent limits were 60 mg O₂ L⁻¹ for BOD, 120 mg O₂ L⁻¹ for COD, 100 mg L⁻¹ for TSS, 10 mg L⁻¹ for TN, and 1 mg L⁻¹ for TP. Probabilities were rounded to the nearest whole percentage. For HCWs, the mean and standard deviation correspond to the effluent of the final treatment stage.

Table S15: Mean effluent concentration (x̄), reliability coefficient (RC), coefficient of variation (CV), allowable mean effluent concentration (mₓ), and observed-to-allowable mean concentration ratio (x̄/mₓ) for BOD, COD, TSS, TN, and TP in the VFCW systems at a target non-exceedance probability of 95%, considering regulatory limits of 60, 120, 100, 10, and 1 mg L⁻¹, respectively.

| **AUTHOR** | **BOD (mg O₂ L⁻¹)** | | | | | **COD (mg O₂ L⁻¹)** | | | | | | **TSS (mg L⁻¹)** | | | | | | **TN (mg L⁻¹)** | | | | | | **TP (mg L⁻¹)** | | | | |
| --- | --- | --- | --- | --- | --- | --- | --- | --- | --- | --- | --- | --- | --- | --- | --- | --- | --- | --- | --- | --- | --- | --- | --- | --- | --- | --- | --- | --- |
|  | **Regulatory limit (Xₛ = 60)** | | | | | **Regulatory limit (Xₛ = 120)** | | | | | | **Regulatory limit (Xₛ = 100)** | | | | | | **Regulatory limit (Xₛ = 10)** | | | | | | **Regulatory limit (Xₛ = 1)** | | | | |
|  | **x̄** | **RC** | **CV** | **mₓ** | **x̄/mₓ** | **x̄** | **RC** | **CV** | **mₓ** | **x̄/mₓ** | **x̄** | | **RC** | **CV** | **mₓ** | **x̄/mₓ** | **x̄** | | **RC** | **CV** | **mₓ** | **x̄/mₓ** | **x̄** | | **RC** | **CV** | **mₓ** | **x̄/mₓ** |
| Suresh et al., 2023 | 12.12 | 0.679 | 0.259 | 40.75 | 0.30 | 55.77 | 0.925 | 0.048 | 111.01 | 0.50 | 8.37 | | 0.665 | 0.275 | 66.54 | 0.13 | — | | — | — | — | — | — | | — | — | — | — |
| Chand et al., 2021 | 6.50 | 0.582 | 0.385 | 34.89 | 0.19 | 18.83 | 0.790 | 0.151 | 94.82 | 0.20 | — | | — | — | — | — | 20.72 | | 0.861 | 0.094 | 8.61 | 2.41 | — | | — | — | — | — |
| Li et al., 2023 | — | — | — | — | — | 14.91 | 0.667 | 0.273 | 80.03 | 0.19 | — | | — | — | — | — | 10.96 | | 0.901 | 0.065 | 9.01 | 1.22 | 0.22 | | 0.630 | 0.318 | 0.63 | 0.35 |
| Pillai and Nair, 2021 | 18.20 | 0.412 | 0.766 | 24.72 | 0.74 | 90.23 | 0.671 | 0.268 | 80.54 | 1.12 | 28.37 | | 0.554 | 0.428 | 55.39 | 0.51 | 29.06 | | 0.764 | 0.174 | 7.64 | 3.80 | — | | — | — | — | — |
| Sun et al., 2021 | — | — | — | — | — | 3.91 | 0.753 | 0.184 | 90.36 | 0.04 | — | | — | — | — | — | 2.07 | | 0.780 | 0.159 | 7.80 | 0.27 | 0.09 | | 0.714 | 0.222 | 0.71 | 0.13 |
| Wu et al., 2015 | — | — | — | — | — | 6.57 | 0.474 | 0.588 | 56.83 | 0.12 | — | | — | — | — | — | 4.69 | | 0.439 | 0.678 | 4.39 | 1.07 | 0.34 | | 0.440 | 0.676 | 0.44 | 0.77 |
| Fan et al., 2013 | — | — | — | — | — | 16.84 | 0.404 | 0.795 | 48.47 | 0.35 | — | | — | — | — | — | 4.72 | | 0.374 | 0.922 | 3.74 | 1.26 | 0.33 | | 0.316 | 1.333 | 0.32 | 1.04 |
| Chang et al., 2012 | — | — | — | — | — | 103.00 | 0.499 | 0.531 | 59.85 | 1.72 | — | | — | — | — | — | 26.40 | | 0.794 | 0.148 | 7.94 | 3.33 | 1.51 | | 0.436 | 0.689 | 0.44 | 3.47 |
| Zurita et al., 2009 | 18.50 | 0.426 | 0.719 | 25.56 | 0.72 | 41.30 | 0.454 | 0.637 | 54.48 | 0.76 | 16.90 | | 0.409 | 0.778 | 40.85 | 0.41 | 14.10 | | 0.737 | 0.200 | 7.37 | 1.91 | 3.80 | | 0.647 | 0.297 | 0.65 | 5.88 |
| Rodrigues et al., 2022 | 20.60 | 0.529 | 0.471 | 31.76 | 0.65 | 73.80 | 0.411 | 0.771 | 49.26 | 1.50 | 12.90 | | 0.307 | 1.434 | 30.72 | 0.42 | — | | — | — | — | — | 1.50 | | 0.618 | 0.333 | 0.62 | 2.43 |
| Santos et al., 2018 | — | — | — | — | — | 45.70 | 0.385 | 0.871 | 46.24 | 0.99 | 2.30 | | 0.267 | 2.435 | 26.69 | 0.09 | 44.30 | | 0.706 | 0.230 | 7.06 | 6.27 | — | | — | — | — | — |
| Sezerino et al., 2023 | 25.00 | 0.661 | 0.280 | 39.65 | 0.63 | 34.00 | 0.365 | 0.971 | 43.78 | 0.78 | 1.00 | | 0.277 | 2.000 | 27.74 | 0.04 | 47.00 | | 0.556 | 0.426 | 5.56 | 8.46 | — | | — | — | — | — |

Note: x̄ = observed mean effluent concentration; RC = Reliability Coefficient; CV = coefficient of variation; mₓ = allowable mean effluent concentration at 95% non-exceedance probability; x̄/mₓ = ratio between the observed and allowable means.

Table S16: Mean effluent concentration (x̄), reliability coefficient (RC), coefficient of variation (CV), allowable mean effluent concentration (mₓ), and observed-to-allowable mean concentration ratio (x̄/mₓ) for BOD, COD, TSS, TN, and TP in the VFCW systems at a target non-exceedance probability of 95%, considering the less stringent benchmark limits adopted in this study: 90, 200, 150, 20, and 4 mg L⁻¹, respectively.

| **AUTHOR** | **BOD (mg O₂ L⁻¹)** | | | | | **COD (mg O₂ L⁻¹)** | | | | | | **TSS (mg L⁻¹)** | | | | | | **TN (mg L⁻¹)** | | | | | | **TP (mg L⁻¹)** | | | | | |
| --- | --- | --- | --- | --- | --- | --- | --- | --- | --- | --- | --- | --- | --- | --- | --- | --- | --- | --- | --- | --- | --- | --- | --- | --- | --- | --- | --- | --- | --- |
|  | **Regulatory limit (Xₛ = 90)** | | | | | **Regulatory limit (Xₛ = 200)** | | | | | | **Regulatory limit (Xₛ = 150)** | | | | | | **Regulatory limit (Xₛ = 20)** | | | | | | **Regulatory limit (Xₛ = 4)** | | | | | |
|  | **x̄** | **RC** | **CV** | **mₓ** | **x̄/mₓ** | **x̄** | **RC** | **CV** | **mₓ** | **x̄/mₓ** | **x̄** | | **RC** | **CV** | **mₓ** | **x̄/mₓ** | **x̄** | | **RC** | **CV** | **mₓ** | **x̄/mₓ** | **x̄** | | **RC** | **CV** | **mₓ** | **x̄/mₓ** |  |
| Suresh et al., 2023 | 12.12 | 0.679 | 0.259 | 61.13 | 0.20 | 55.77 | 0.925 | 0.048 | 185.02 | 0.30 | 8.37 | | 0.665 | 0.275 | 99.80 | 0.08 | — | | — | — | — | — | — | | — | — | — | — |  |
| Chand et al., 2021 | 6.50 | 0.582 | 0.385 | 52.34 | 0.12 | 18.83 | 0.790 | 0.151 | 158.04 | 0.12 | — | | — | — | — | — | 20.72 | | 0.861 | 0.094 | 17.21 | 1.20 | — | | — | — | — | — |  |
| Li et al., 2023 | — | — | — | — | — | 14.91 | 0.667 | 0.273 | 133.39 | 0.11 | — | | — | — | — | — | 10.96 | | 0.901 | 0.065 | 18.02 | 0.61 | 0.22 | | 0.630 | 0.318 | 2.52 | 0.09 |  |
| Pillai and Nair, 2021 | 18.20 | 0.412 | 0.766 | 37.08 | 0.49 | 90.23 | 0.671 | 0.268 | 134.24 | 0.67 | 28.37 | | 0.554 | 0.428 | 83.08 | 0.34 | 29.06 | | 0.764 | 0.174 | 15.28 | 1.90 | — | | — | — | — | — |  |
| Sun et al., 2021 | — | — | — | — | — | 3.91 | 0.753 | 0.184 | 150.60 | 0.03 | — | | — | — | — | — | 2.07 | | 0.780 | 0.159 | 15.61 | 0.13 | 0.09 | | 0.714 | 0.222 | 2.86 | 0.03 |  |
| Wu et al., 2015 | — | — | — | — | — | 6.57 | 0.474 | 0.588 | 94.71 | 0.07 | — | | — | — | — | — | 4.69 | | 0.439 | 0.678 | 8.79 | 0.53 | 0.34 | | 0.440 | 0.676 | 1.76 | 0.19 |  |
| Fan et al., 2013 | — | — | — | — | — | 16.84 | 0.404 | 0.795 | 80.79 | 0.21 | — | | — | — | — | — | 4.72 | | 0.374 | 0.922 | 7.49 | 0.63 | 0.33 | | 0.316 | 1.333 | 1.26 | 0.26 |  |
| Chang et al., 2012 | — | — | — | — | — | 103.00 | 0.499 | 0.531 | 99.74 | 1.03 | — | | — | — | — | — | 26.40 | | 0.794 | 0.148 | 15.88 | 1.66 | 1.51 | | 0.436 | 0.689 | 1.74 | 0.87 |  |
| Zurita et al., 2009 | 18.50 | 0.426 | 0.719 | 38.33 | 0.48 | 41.30 | 0.454 | 0.637 | 90.80 | 0.45 | 16.90 | | 0.409 | 0.778 | 61.28 | 0.28 | 14.10 | | 0.737 | 0.200 | 14.73 | 0.96 | 3.80 | | 0.647 | 0.297 | 2.59 | 1.47 |  |
| Rodrigues et al., 2022 | 20.60 | 0.529 | 0.471 | 47.65 | 0.43 | 73.80 | 0.411 | 0.771 | 82.11 | 0.90 | 12.90 | | 0.307 | 1.434 | 46.08 | 0.28 | — | | — | — | — | — | 1.50 | | 0.618 | 0.333 | 2.47 | 0.61 |  |
| Santos et al., 2018 | — | — | — | — | — | 45.70 | 0.385 | 0.871 | 77.07 | 0.59 | 2.30 | | 0.267 | 2.435 | 40.04 | 0.06 | 44.30 | | 0.706 | 0.230 | 14.12 | 3.14 | — | | — | — | — | — |  |
| Sezerino et al., 2023 | 25.00 | 0.661 | 0.280 | 59.48 | 0.42 | 34.00 | 0.365 | 0.971 | 72.97 | 0.47 | 1.00 | | 0.277 | 2.000 | 41.61 | 0.02 | 47.00 | | 0.556 | 0.426 | 11.11 | 4.23 | — | | — | — | — | — |  |

Table S17: Mean effluent concentration (x̄), reliability coefficient (RC), coefficient of variation (CV), allowable mean effluent concentration (mₓ), and observed-to-allowable mean concentration ratio (x̄/mₓ) for BOD, COD, TSS, TN, and TP in the HFCW systems at a target non-exceedance probability of 95%, considering regulatory limits of 60, 120, 100, 10, and 1 mg L⁻¹, respectively.

| **AUTHOR** | **BOD (mg O₂ L⁻¹)** | | | | | **COD (mg O₂ L⁻¹)** | | | | | | **TSS (mg L⁻¹)** | | | | | | **TN (mg L⁻¹)** | | | | | | **TP (mg L⁻¹)** | | | | |
| --- | --- | --- | --- | --- | --- | --- | --- | --- | --- | --- | --- | --- | --- | --- | --- | --- | --- | --- | --- | --- | --- | --- | --- | --- | --- | --- | --- | --- |
|  | **Regulatory limit (Xₛ = 60)** | | | | | **Regulatory limit (Xₛ = 120)** | | | | | | **Regulatory limit (Xₛ = 100)** | | | | | | **Regulatory limit (Xₛ = 10)** | | | | | | **Regulatory limit (Xₛ = 1)** | | | | |
|  | **x̄** | **RC** | **CV** | **mₓ** | **x̄/mₓ** | **x̄** | **RC** | **CV** | **mₓ** | **x̄/mₓ** | **x̄** | | **RC** | **CV** | **mₓ** | **x̄/mₓ** | **x̄** | | **RC** | **CV** | **mₓ** | **x̄/mₓ** | **x̄** | | **RC** | **CV** | **mₓ** | **x̄/mₓ** |
| Chen et al., 2022 | — | — | — | — | — | 59.80 | 0.518 | 0.492 | 62.20 | 0.96 | — | | — | — | — | — | 22.00 | | 0.539 | 0.454 | 5.39 | 4.08 | 0.12 | | 0.475 | 0.583 | 0.48 | 0.25 |
| Angassa et al., 2019 | — | — | — | — | — | 30.00 | 0.571 | 0.400 | 68.58 | 0.44 | — | | — | — | — | — | 2.30 | | 0.525 | 0.478 | 5.25 | 0.44 | 4.30 | | 0.467 | 0.605 | 0.47 | 9.22 |
| Wang and Li, 2017 | 3.59 | 0.416 | 0.752 | 24.96 | 0.14 | 42.65 | 0.537 | 0.457 | 64.47 | 0.66 | — | | — | — | — | — | 21.34 | | 0.686 | 0.252 | 6.86 | 3.11 | — | | — | — | — | — |
| Mburu et al., 2013 | 29.00 | 0.636 | 0.310 | 38.15 | 0.76 | 58.00 | 0.644 | 0.300 | 77.30 | 0.75 | 19.00 | | 0.537 | 0.458 | 53.66 | 0.35 | — | | — | — | — | — | 3.00 | | 0.853 | 0.100 | 0.85 | 3.52 |
| Zurita et al., 2009 | 21.20 | 0.506 | 0.516 | 30.37 | 0.70 | 51.30 | 0.626 | 0.323 | 75.14 | 0.68 | 8.50 | | 0.340 | 1.123 | 34.04 | 0.25 | 13.80 | | 0.718 | 0.218 | 7.18 | 1.92 | 4.90 | | 0.706 | 0.230 | 0.71 | 6.94 |
| Ho et al., 2020 | 7.10 | 0.964 | 0.023 | 57.83 | 0.12 | 20.83 | 0.460 | 0.621 | 55.22 | 0.38 | 18.23 | | 0.631 | 0.317 | 63.05 | 0.29 | 18.30 | | 0.610 | 0.344 | 6.10 | 3.00 | 1.78 | | 0.572 | 0.399 | 0.57 | 3.11 |
| Ergaieg et al., 2021 (system 1) | 12.26 | 0.615 | 0.338 | 36.88 | 0.33 | 47.79 | 0.595 | 0.365 | 71.42 | 0.67 | 15.99 | | 0.617 | 0.335 | 61.66 | 0.26 | — | | — | — | — | — | — | | — | — | — | — |
| Ergaieg et al., 2021 (system 2) | 14.77 | 0.743 | 0.194 | 44.57 | 0.33 | 50.74 | 0.642 | 0.302 | 77.09 | 0.66 | 19.53 | | 0.734 | 0.202 | 73.39 | 0.27 | — | | — | — | — | — | — | | — | — | — | — |
| Sousa et al., 2024 | 27.00 | 0.374 | 0.926 | 22.41 | 1.20 | 76.00 | 0.456 | 0.632 | 54.72 | 1.39 | 82.00 | | 0.570 | 0.402 | 56.99 | 1.44 | — | | — | — | — | — | — | | — | — | — | — |
| Silva et al., 2017 | 38.00 | 0.653 | 0.289 | 39.17 | 0.97 | 47.00 | 0.543 | 0.447 | 65.15 | 0.72 | 10.00 | | 0.360 | 1.000 | 35.95 | 0.28 | — | | — | — | — | — | — | | — | — | — | — |

Table S18: Mean effluent concentration (x̄), reliability coefficient (RC), coefficient of variation (CV), allowable mean effluent concentration (mₓ), and observed-to-allowable mean concentration ratio (x̄/mₓ) for BOD, COD, TSS, TN, and TP in the HFCW systems at a target non-exceedance probability of 95%, considering the less stringent benchmark limits adopted in this study: 90, 200, 150, 20, and 4 mg L⁻¹, respectively.

| **AUTHOR** | **BOD (mg O₂ L⁻¹)** | | | | | **COD (mg O₂ L⁻¹)** | | | | | **TSS (mg L⁻¹)** | | | | | | **TN (mg L⁻¹)** | | | | | | **TP (mg L⁻¹)** | | | | | |
| --- | --- | --- | --- | --- | --- | --- | --- | --- | --- | --- | --- | --- | --- | --- | --- | --- | --- | --- | --- | --- | --- | --- | --- | --- | --- | --- | --- | --- |
|  | **Regulatory limit (Xₛ = 90)** | | | | | **Regulatory limit (Xₛ = 200)** | | | | | **Regulatory limit (Xₛ = 150)** | | | | | | **Regulatory limit (Xₛ = 20)** | | | | | | **Regulatory limit (Xₛ = 4)** | | | | | |
|  | **x̄** | **RC** | **CV** | **mₓ** | **x̄/mₓ** | **x̄** | **RC** | **CV** | **mₓ** | **x̄/mₓ** | **x̄** | **RC** | **CV** | **mₓ** | **x̄/mₓ** | **x̄** | | **RC** | **CV** | **mₓ** | **x̄/mₓ** | **x̄** | | **RC** | **CV** | **mₓ** | **x̄/mₓ** |  |
| Chen et al., 2022 | — | — | — | — | — | 59.80 | 0.518 | 0.492 | 103.67 | 0.58 | — | — | — | — | — | 22.00 | | 0.539 | 0.454 | 10.78 | 2.04 | 0.12 | | 0.475 | 0.583 | 1.90 | 0.06 |  |
| Angassa et al., 2019 | — | — | — | — | — | 30.00 | 0.571 | 0.400 | 114.30 | 0.26 | — | — | — | — | — | 2.30 | | 0.525 | 0.478 | 10.51 | 0.22 | 4.30 | | 0.467 | 0.605 | 1.87 | 2.30 |  |
| Wang and Li, 2017 | 3.59 | 0.416 | 0.752 | 37.43 | 0.10 | 42.65 | 0.537 | 0.457 | 107.45 | 0.40 | — | — | — | — | — | 21.34 | | 0.686 | 0.252 | 13.71 | 1.56 | — | | — | — | — | — |  |
| Mburu et al., 2013 | 29.00 | 0.636 | 0.310 | 57.22 | 0.51 | 58.00 | 0.644 | 0.300 | 128.83 | 0.45 | 19.00 | 0.537 | 0.458 | 80.49 | 0.24 | — | | — | — | — | — | 3.00 | | 0.853 | 0.100 | 3.41 | 0.88 |  |
| Zurita et al., 2009 | 21.20 | 0.506 | 0.516 | 45.55 | 0.47 | 51.30 | 0.626 | 0.323 | 125.24 | 0.41 | 8.50 | 0.340 | 1.123 | 51.05 | 0.17 | 13.80 | | 0.718 | 0.218 | 14.37 | 0.96 | 4.90 | | 0.706 | 0.230 | 2.82 | 1.73 |  |
| Ho et al., 2020 | 7.10 | 0.964 | 0.023 | 86.75 | 0.08 | 20.83 | 0.460 | 0.621 | 92.03 | 0.23 | 18.23 | 0.631 | 0.317 | 94.58 | 0.19 | 18.30 | | 0.610 | 0.344 | 12.20 | 1.50 | 1.78 | | 0.572 | 0.399 | 2.29 | 0.78 |  |
| Ergaieg et al., 2021 (system 1) | 12.26 | 0.615 | 0.338 | 55.33 | 0.22 | 47.79 | 0.595 | 0.365 | 119.04 | 0.40 | 15.99 | 0.617 | 0.335 | 92.49 | 0.17 | — | | — | — | — | — | — | | — | — | — | — |  |
| Ergaieg et al., 2021 (system 2) | 14.77 | 0.743 | 0.194 | 66.86 | 0.22 | 50.74 | 0.642 | 0.302 | 128.48 | 0.39 | 19.53 | 0.734 | 0.202 | 110.09 | 0.18 | — | | — | — | — | — | — | | — | — | — | — |  |
| Sousa et al., 2024 | 27.00 | 0.374 | 0.926 | 33.62 | 0.80 | 76.00 | 0.456 | 0.632 | 91.20 | 0.83 | 82.00 | 0.570 | 0.402 | 85.49 | 0.96 | — | | — | — | — | — | — | | — | — | — | — |  |
| Silva et al., 2017 | 38.00 | 0.653 | 0.289 | 58.76 | 0.65 | 47.00 | 0.543 | 0.447 | 108.58 | 0.43 | 10.00 | 0.360 | 1.000 | 53.93 | 0.19 | — | | — | — | — | — | — | | — | — | — | — |  |

Table S19: Mean effluent concentration (x̄), reliability coefficient (RC), coefficient of variation (CV), allowable mean effluent concentration (mₓ), and observed-to-allowable mean concentration ratio (x̄/mₓ) for BOD, COD, TSS, TN, and TP in the HCW systems at a target non-exceedance probability of 95%, considering regulatory limits of 60, 120, 100, 10, and 1 mg L⁻¹, respectively.

| **AUTHOR** | **BOD (mg O₂ L⁻¹)** | | | | | **COD (mg O₂ L⁻¹)** | | | | | | **TSS (mg L⁻¹)** | | | | | | **TN (mg L⁻¹)** | | | | | | **TP (mg L⁻¹)** | | | | | |
| --- | --- | --- | --- | --- | --- | --- | --- | --- | --- | --- | --- | --- | --- | --- | --- | --- | --- | --- | --- | --- | --- | --- | --- | --- | --- | --- | --- | --- | --- |
|  | **Regulatory limit (Xₛ = 60)** | | | | | **Regulatory limit (Xₛ = 120)** | | | | | | **Regulatory limit (Xₛ = 100)** | | | | | | **Regulatory limit (Xₛ = 10)** | | | | | | **Regulatory limit (Xₛ = 1)** | | | | | |
|  | **x̄** | **RC** | **CV** | **mₓ** | **x̄/mₓ** | **x̄** | **RC** | **CV** | **mₓ** | **x̄/mₓ** | **x̄** | | **RC** | **CV** | **mₓ** | **x̄/mₓ** | **x̄** | | **RC** | **CV** | **mₓ** | **x̄/mₓ** | **x̄** | | **RC** | **CV** | **mₓ** | **x̄/mₓ** |  |
| Martel-Rodríguez et al., 2022 | 60.00 | 0.514 | 0.500 | 30.84 | 1.95 | 124.00 | 0.602 | 0.355 | 72.26 | 1.72 | 14.00 | | 0.289 | 1.714 | 28.92 | 0.48 | 56.00 | | 0.641 | 0.304 | 6.41 | 8.73 | — | | — | — | — | — |  |
| Zhai et al., 2016 | — | — | — | — | — | 35.50 | 0.525 | 0.479 | 63.01 | 0.56 | — | | — | — | — | — | 12.20 | | 0.565 | 0.410 | 5.65 | 2.16 | 0.90 | | 0.443 | 0.667 | 0.44 | 2.03 |  |
| Marzec et al., 2024 | 1.96 | 0.373 | 0.929 | 22.38 | 0.09 | 26.46 | 0.524 | 0.480 | 62.94 | 0.42 | 12.08 | | 0.321 | 1.286 | 32.08 | 0.38 | — | | — | — | — | — | — | | — | — | — | — |  |
| Ye and Li, 2009 | — | — | — | — | — | 49.30 | 0.659 | 0.282 | 79.10 | 0.62 | 14.20 | | 0.575 | 0.394 | 57.51 | 0.25 | 11.90 | | 0.622 | 0.328 | 6.22 | 1.91 | 2.23 | | 0.553 | 0.430 | 0.55 | 4.04 |  |
| Micek et al., 2020 (system 1) | 3.50 | 0.437 | 0.686 | 26.20 | 0.13 | 34.80 | 0.547 | 0.440 | 65.65 | 0.53 | 26.70 | | 0.480 | 0.573 | 47.97 | 0.56 | 39.90 | | 0.503 | 0.521 | 5.03 | 7.93 | 2.60 | | 0.411 | 0.769 | 0.41 | 6.33 |  |
| Micek et al., 2020 (system 2) | 3.00 | 0.468 | 0.600 | 28.10 | 0.11 | 15.20 | 0.539 | 0.454 | 64.66 | 0.24 | 9.90 | | 0.433 | 0.697 | 43.30 | 0.23 | 13.80 | | 0.369 | 0.949 | 3.69 | 3.74 | 1.10 | | 0.538 | 0.455 | 0.54 | 2.04 |  |
| Marzec et al., 2019 | 6.60 | 0.312 | 1.379 | 18.71 | 0.35 | 31.80 | 0.453 | 0.638 | 54.42 | 0.58 | 18.00 | | 0.340 | 1.122 | 34.04 | 0.53 | 56.40 | | 0.409 | 0.775 | 4.09 | 13.77 | 6.70 | | 0.579 | 0.388 | 0.58 | 11.57 |  |
| Gajewska et al., 2018 | 12.20 | 0.394 | 0.836 | 23.61 | 0.52 | 46.40 | 0.429 | 0.709 | 51.49 | 0.90 | — | | — | — | — | — | 70.80 | | 0.565 | 0.410 | 5.65 | 12.52 | — | | — | — | — | — |  |
| Gajewska et al., 2018 | 11.70 | 0.277 | 2.009 | 16.63 | 0.70 | 40.40 | 0.303 | 1.485 | 36.40 | 1.11 | — | | — | — | — | — | 54.30 | | 0.507 | 0.514 | 5.07 | 10.71 | — | | — | — | — | — |  |
| Silva and Bueno, 2015 | — | — | — | — | — | 93.00 | 0.462 | 0.616 | 55.44 | 1.68 | 12.00 | | 0.452 | 0.642 | 45.22 | 0.27 | — | | — | — | — | — | 1.80 | | 0.714 | 0.222 | 0.71 | 2.52 |  |
| Freitas, 2017 | 26.00 | 0.557 | 0.423 | 33.42 | 0.78 | 24.30 | 0.517 | 0.494 | 62.06 | 0.39 | 2.40 | | 0.280 | 1.917 | 28.04 | 0.09 | 20.20 | | 0.492 | 0.545 | 4.92 | 4.10 | — | | — | — | — | — |  |
| Rousso, 2017 | — | — | — | — | — | 12.00 | 0.417 | 0.750 | 49.98 | 0.24 | 0.04 | | 0.271 | 2.250 | 27.06 | 0.00 | 24.81 | | 0.545 | 0.444 | 5.45 | 4.56 | — | | — | — | — | — |  |

Table S20: Mean effluent concentration (x̄), reliability coefficient (RC), coefficient of variation (CV), allowable mean effluent concentration (mₓ), and observed-to-allowable mean concentration ratio (x̄/mₓ) for BOD, COD, TSS, TN, and TP in the HCW systems at a target non-exceedance probability of 95%, considering the less stringent benchmark limits adopted in this study: 90, 200, 150, 20, and 4 mg L⁻¹, respectively.

| **AUTHOR** | **BOD (mg O₂ L⁻¹)** | | | | | **COD (mg O₂ L⁻¹)** | | | | | | **TSS (mg L⁻¹)** | | | | | | **TN (mg L⁻¹)** | | | | | | **TP (mg L⁻¹)** | | | | | |
| --- | --- | --- | --- | --- | --- | --- | --- | --- | --- | --- | --- | --- | --- | --- | --- | --- | --- | --- | --- | --- | --- | --- | --- | --- | --- | --- | --- | --- | --- |
|  | **Regulatory limit (Xₛ = 90)** | | | | | **Regulatory limit (Xₛ = 200)** | | | | | | **Regulatory limit (Xₛ = 150)** | | | | | | **Regulatory limit (Xₛ = 20)** | | | | | | **Regulatory limit (Xₛ = 4)** | | | | | |
|  | **x̄** | **RC** | **CV** | **mₓ** | **x̄/mₓ** | **x̄** | **RC** | **CV** | **mₓ** | **x̄/mₓ** | **x̄** | | **RC** | **CV** | **mₓ** | **x̄/mₓ** | **x̄** | | **RC** | **CV** | **mₓ** | **x̄/mₓ** | **x̄** | | **RC** | **CV** | **mₓ** | **x̄/mₓ** |  |
| Martel-Rodríguez et al., 2022 | 60.00 | 0.514 | 0.500 | 46.26 | 1.30 | 124.00 | 0.602 | 0.355 | 120.44 | 1.03 | 14.00 | | 0.289 | 1.714 | 43.38 | 0.32 | 56.00 | | 0.641 | 0.304 | 12.83 | 4.37 | — | | — | — | — | — |  |
| Zhai et al., 2016 | — | — | — | — | — | 35.50 | 0.525 | 0.479 | 105.01 | 0.34 | — | | — | — | — | — | 12.20 | | 0.565 | 0.410 | 11.30 | 1.08 | 0.90 | | 0.443 | 0.667 | 1.77 | 0.51 |  |
| Marzec et al., 2024 | 1.96 | 0.373 | 0.929 | 33.57 | 0.06 | 26.46 | 0.524 | 0.480 | 104.90 | 0.25 | 12.08 | | 0.321 | 1.286 | 48.12 | 0.25 | — | | — | — | — | — | — | | — | — | — | — |  |
| Ye and Li, 2009 | — | — | — | — | — | 49.30 | 0.659 | 0.282 | 131.84 | 0.37 | 14.20 | | 0.575 | 0.394 | 86.27 | 0.16 | 11.90 | | 0.622 | 0.328 | 12.44 | 0.96 | 2.23 | | 0.553 | 0.430 | 2.21 | 1.01 |  |
| Micek et al., 2020 (system 1) | 3.50 | 0.437 | 0.686 | 39.30 | 0.09 | 34.80 | 0.547 | 0.440 | 109.42 | 0.32 | 26.70 | | 0.480 | 0.573 | 71.95 | 0.37 | 39.90 | | 0.503 | 0.521 | 10.07 | 3.96 | 2.60 | | 0.411 | 0.769 | 1.64 | 1.58 |  |
| Micek et al., 2020 (system 2) | 3.00 | 0.468 | 0.600 | 42.16 | 0.07 | 15.20 | 0.539 | 0.454 | 107.76 | 0.14 | 9.90 | | 0.433 | 0.697 | 64.94 | 0.15 | 13.80 | | 0.369 | 0.949 | 7.38 | 1.87 | 1.10 | | 0.538 | 0.455 | 2.15 | 0.51 |  |
| Marzec et al., 2019 | 6.60 | 0.312 | 1.379 | 28.07 | 0.24 | 31.80 | 0.453 | 0.638 | 90.69 | 0.35 | 18.00 | | 0.340 | 1.122 | 51.06 | 0.35 | 56.40 | | 0.409 | 0.775 | 8.19 | 6.89 | 6.70 | | 0.579 | 0.388 | 2.32 | 2.89 |  |
| Gajewska et al., 2018 | 12.20 | 0.394 | 0.836 | 35.42 | 0.34 | 46.40 | 0.429 | 0.709 | 85.81 | 0.54 | — | | — | — | — | — | 70.80 | | 0.565 | 0.410 | 11.31 | 6.26 | — | | — | — | — | — |  |
| Gajewska et al., 2018 | 11.70 | 0.277 | 2.009 | 24.94 | 0.47 | 40.40 | 0.303 | 1.485 | 60.66 | 0.67 | — | | — | — | — | — | 54.30 | | 0.507 | 0.514 | 10.14 | 5.35 | — | | — | — | — | — |  |
| Silva and Bueno, 2015 | — | — | — | — | — | 93.00 | 0.462 | 0.616 | 92.39 | 1.01 | 12.00 | | 0.452 | 0.642 | 67.84 | 0.18 | — | | — | — | — | — | 1.80 | | 0.714 | 0.222 | 2.86 | 0.63 |  |
| Freitas, 2017 | 26.00 | 0.557 | 0.423 | 50.13 | 0.52 | 24.30 | 0.517 | 0.494 | 103.44 | 0.23 | 2.40 | | 0.280 | 1.917 | 42.05 | 0.06 | 20.20 | | 0.492 | 0.545 | 9.85 | 2.05 | — | | — | — | — | — |  |
| Rousso, 2017 | — | — | — | — | — | 12.00 | 0.417 | 0.750 | 83.31 | 0.14 | 0.04 | | 0.271 | 2.250 | 40.58 | 0.00 | 24.81 | | 0.545 | 0.444 | 10.89 | 2.28 | — | | — | — | — | — |  |

Table S21: Participation of synthetic-wastewater systems and sensitivity analyses of effluent coefficient of variation (CV) and Reliability Coefficient (RC) results to wastewater type.

**A. Synthetic-wastewater systems included in the dataset**

| **Study** | **Configuration** | **Scale** | **Parameters with CV/RC** | **Main experimental controls** |
| --- | --- | --- | --- | --- |
| Li et al., 2023 | VFCW | Pilot | COD, TN, TP | Intermittent aeration; static magnetic field |
| Sun et al., 2021 | VFCW | Mesocosm | COD, TN, TP | Modified matrix; microbial amendment; carbon supplementation |
| Wu et al. (2015) | VFCW | Microcosm | COD, TN, TP | Batch feeding; intermittent aeration |
| Fan et al. (2013) | VFCW | Microcosm | COD, TN, TP | Manipulated COD/N ratios; intermittent aeration |
| Chang et al. (2012) | VFCW | Pilot | COD, TN, TP | Prepared influent; integrated down-flow/up-flow units |
| Chen et al. (2022) | HFCW | Pilot | COD, TN, TP | Prepared influent; aerated filtration chamber |

**B. Direct comparison of synthetic and real wastewater**

| **Parameter** | **n syn.** | **n real** | **Median CV syn.** | **Median CV real** | **Median RC syn.** | **Median RC real** | **U** | **Exact p** | **BH p-adj.** | **Cliff’s δ** |
| --- | --- | --- | --- | --- | --- | --- | --- | --- | --- | --- |
| COD | 6 | 28 | 0.511353 | 0.467807 | 0.508522 | 0.531156 | 80.0 | 0.877611 | 0.877611 | -0.0476 |
| TN | 6 | 19 | 0.306528 | 0.409605 | 0.659659 | 0.565357 | 50.0 | 0.687047 | 0.877611 | -0.1228 |
| TP | 6 | 12 | 0.629902 | 0.393468 | 0.457566 | 0.575741 | 48.5 | 0.290778 | 0.872334 | 0.3472 |

**C. Exact permutation sensitivity analysis stratified by flow configuration**

| **Parameter** | **n VFCW/HFCW** | **Synthetic n VFCW/HFCW** | **Synthetic coefficient in log(CV)** | **Exact permutations** | **Exact p** | **BH p-adj.** |
| --- | --- | --- | --- | --- | --- | --- |
| COD | 12/10 | 5/1 | 0.149222 | 7920 | 0.753157 | 0.753157 |
| TN | 10/5 | 5/1 | 0.259123 | 1260 | 0.550794 | 0.753157 |
| TP | 7/5 | 5/1 | 0.613266 | 105 | 0.190476 | 0.571429 |

**D. Sensitivity analysis after aggregation to the source-study level**

| **Parameter** | **n synthetic sources** | **n real sources** | **Median CV syn.** | **Median CV real** | **U** | **Exact p** | **BH p-adj.** | **Cliff’s δ** |
| --- | --- | --- | --- | --- | --- | --- | --- | --- |
| COD | 6 | 25 | 0.511353 | 0.479750 | 71.0 | 0.864670 | 0.864670 | -0.0533 |
| TN | 6 | 17 | 0.306528 | 0.409605 | 46.0 | 0.759171 | 0.864670 | -0.0980 |
| TP | 6 | 10 | 0.629902 | 0.393468 | 41.5 | 0.263487 | 0.790460 | 0.3833 |

**E. Configuration-level RC comparisons before and after exclusion of synthetic-wastewater systems**

| **Parameter** | **Dataset** | **n VFCW/HFCW/HCW** | **Mean RC VFCW/HFCW/HCW** | **Median RC VFCW/HFCW/HCW** | **H** | **p** | **ε²** |
| --- | --- | --- | --- | --- | --- | --- | --- |
| BOD | All systems | 6/8/8 | 0.548159/0.613210/0.416456 | 0.555484/0.625272/0.415110 | 6.118577 | 0.046921 | 0.216767 |
| BOD | Real wastewater only | 6/8/8 | 0.548159/0.613210/0.416456 | 0.555484/0.625272/0.415110 | 6.118577 | 0.046921 | 0.216767 |
| COD | All systems | 12/10/12 | 0.566444/0.559408/0.498195 | 0.486130/0.557194/0.520838 | 1.663866 | 0.435207 | 0.000000 |
| COD | Real wastewater only | 7/9/12 | 0.571601/0.563973/0.498195 | 0.454008/0.571477/0.520838 | 2.129310 | 0.344847 | 0.005172 |
| TSS | All systems | 6/7/9 | 0.413214/0.541059/0.382371 | 0.357865/0.569910/0.340412 | 4.631846 | 0.098675 | 0.138518 |
| TSS | Real wastewater only | 6/7/9 | 0.413214/0.541059/0.382371 | 0.357865/0.569910/0.340412 | 4.631846 | 0.098675 | 0.138518 |
| TN | All systems | 10/5/10 | 0.691186/0.615647/0.521997 | 0.750461/0.609858/0.525876 | 6.225231 | 0.044484 | 0.192056 |
| TN | Real wastewater only | 5/4/10 | 0.724629/0.634808/0.521997 | 0.736676/0.647703/0.525876 | 8.641579 | 0.013289 | 0.415099 |
| TP | All systems | 7/5/6 | 0.542819/0.614606/0.539733 | 0.617995/0.572202/0.545499 | 0.729784 | 0.694272 | 0.000000 |
| TP | Real wastewater only | 2/4/6 | 0.632344/0.649433/0.539733 | 0.632344/0.639156/0.545499 | 2.134615 | 0.343933 | 0.014957 |

**F. Dunn pairwise comparisons for significant Kruskal–Wallis tests**

| **Parameter** | **Dataset** | **Comparison** | **z** | **Unadjusted p** | **BH p-adj.** |
| --- | --- | --- | --- | --- | --- |
| BOD | All systems (= real-only) | VFCW vs HFCW | -0.285149 | 0.775530 | 0.775530 |
| BOD | All systems (= real-only) | VFCW vs HCW | 1.853469 | 0.063815 | 0.095723 |
| BOD | All systems (= real-only) | HFCW vs HCW | 2.309972 | 0.020890 | 0.062669 |
| TN | All systems | VFCW vs HFCW | 0.893050 | 0.371830 | 0.371830 |
| TN | All systems | VFCW vs HCW | 2.491339 | 0.012726 | 0.038179 |
| TN | All systems | HFCW vs HCW | 1.141120 | 0.253820 | 0.371830 |
| TN | Real wastewater only | VFCW vs HFCW | 0.966909 | 0.333590 | 0.333590 |
| TN | Real wastewater only | VFCW vs HCW | 2.855097 | 0.004302 | 0.012907 |
| TN | Real wastewater only | HFCW vs HCW | 1.546935 | 0.121879 | 0.182818 |

Notes: RC values correspond to a target non-exceedance probability of 95%. “Synthetic” denotes prepared wastewater intended to simulate domestic sewage. No synthetic-wastewater system contributed BOD or TSS data.

Panel B: exact two-sided Mann–Whitney tests were applied to effluent CV. At 95%, RC is a strictly decreasing transformation of CV over the observed range; therefore, rank-based inference is identical for RC, with the direction reversed. Benjamini–Hochberg adjustment was applied across COD, TN, and TP. Positive Cliff’s δ indicates higher CV in synthetic-wastewater systems.

Panel C: the dependent variable was log(CV). The synthetic-wastewater coefficient was estimated with flow configuration included as a fixed effect. Wastewater-type labels were permuted within VFCW and HFCW strata while preserving the observed number of synthetic systems. HCWs did not contribute because all HCWs treated real wastewater.

Panel D: when a source reported more than one system, system-level CVs were aggregated using the median before the exact Mann–Whitney test.

Panel E: Kruskal–Wallis p-values use the standard chi-square approximation with tie-corrected ranks. Epsilon-squared was truncated at zero when the estimator was negative. Panel F: Dunn tests used tie-corrected rank variance and Benjamini–Hochberg adjustment within each global comparison.

Table S22: Descriptive statistics of mean Reliability Coefficients (RCs) and coefficients of variation (CVs) by parameter and constructed wetland configuration at a target non-exceedance probability of 95%.

| **Parameter** | **VFCW** | | **HFCW** | | **HCW** | |
| --- | --- | --- | --- | --- | --- | --- |
|  | **Mean RC** | **Mean CV** | **Mean RC** | **Mean CV** | **Mean RC** | **Mean CV** |
| COD | 0.5664 | 0.5073 | 0.5594 | 0.4337 | 0.4982 | 0.5985 |
| BOD | 0.5482 | 0.4799 | 0.6132 | 0.4184 | 0.4165 | 0.9201 |
| TSS | 0.4132 | 1.2250 | 0.5411 | 0.5482 | 0.3824 | 1.1772 |
| TN | 0.6912 | 0.3095 | 0.6156 | 0.3492 | 0.5220 | 0.5198 |
| TP | 0.5428 | 0.5527 | 0.6146 | 0.3834 | 0.5397 | 0.4885 |

**Sensitivity to the target non-exceedance probability and ranking stability — Supplementary Method S1**

Sensitivity of the RC to the target non-exceedance probability was evaluated using the 121 observed CV values available across all system–parameter combinations. Each CV retained its association with the corresponding system, flow configuration, and water-quality parameter. The RC was recalculated at target non-exceedance probabilities of 50%, 60%, 70%, 80%, 90%, 95%, 98%, 99%, and 99.9%, using the corresponding standard normal quantile $Z_{1-\alpha}$.

For a fixed target non-exceedance probability, the RC is a deterministic function of the CV. To identify the probability-specific turning point of this function, the following transformation was defined:

$$y={[\ln(1+{CV}^{2})]}^{\frac{1}{2}}$$

(S1)

Because $\left( 1+CV^{2} \right)^{1/2}=\exp\left( y^{2}/2 \right)$, Equation (4) of the main manuscript can be rewritten as:

$$RC=\exp[\frac{y^{2}}{2}-Z_{1-\alpha}y]$$

(S2)

Differentiating the transformed expression with respect to $y$gives:

$$\frac{dRC}{dy}=RC\left( y-Z_{1-\alpha} \right)$$

(S3)

Because $RC>0$, the derivative changes sign when $y=Z_{1-\alpha}$. Substituting this condition into Equation (S1) gives the probability-specific critical coefficient of variation

$${CV}_{crit}={[\exp({(Z_{1-\alpha})}^{2})-1]}^{\frac{1}{2}}$$

(S4)

For CV values below $CV_{\mathrm{crit}}$, increasing CV decreases the RC. Above this point, the analytical expression becomes non-monotonic and an increase in CV may increase the calculated RC. This reversal is a mathematical property of the formulation and does not represent improved treatment stability, effluent quality, or pollutant removal. RC values greater than one generated at low target probabilities were retained to describe the complete mathematical behavior of the equation but were not interpreted as evidence of improved performance.

For each water-quality parameter, systems were ordered from the highest to the lowest RC using unrounded values. The ordering obtained at the 95% target non-exceedance probability was used as the reference and compared with the orderings obtained at each alternative probability. Agreement was evaluated using Spearman’s rank correlation coefficient ($\rho$), Kendall’s tau-b ($\tau_{b}$), and the number of discordant system pairs. Retention of the highest-ranked system was also recorded separately from preservation of the complete system ordering. Configuration-level means were calculated only for descriptive and graphical comparisons and were not interpreted as transferable configuration-specific coefficients.

The configuration-level RC values obtained at each target probability are presented in Supplementary Table S23.

**Sensitivity results for the target non-exceedance probability and ranking stability**

At target probabilities of 95%, 98%, 99%, and 99.9%, the critical CVs were 3.738, 8.183, 14.923, and 118.394, respectively. All exceeded the maximum observed CV of 2.435. At 90%, the critical CV was 2.043, and only two TSS observations exceeded this value. The response for these probabilities is presented in Figure 9 of the main manuscript.

At the lower target probabilities, the critical CV occurred within the observed range. The critical values were 0.257, 0.563, and 1.016 at 60%, 70%, and 80%, respectively. Of the 121 observations, 102 exceeded the critical CV at 60%, 48 at 70%, and 13 at 80%. The corresponding curves therefore decreased to a minimum and subsequently increased after the turning point. At 50%, $Z_{1-\alpha}=0$, and the equation reduces to $RC=\sqrt{1+CV^{2}}$; all positive CV values are consequently located on the increasing branch.

Figure S1: Relationship between the Reliability Coefficient (RC) and the observed coefficient of variation (CV) at target non-exceedance probabilities of 50%, 60%, 70%, and 80%.

This mathematical behavior produced RC values greater than one for all 121 observations at 50%, for 49 observations at 60%, and for eight observations at 70%. No RC exceeded one at target probabilities of 80% or higher. An RC greater than one under a low target probability does not denote greater stability, improved effluent quality, or enhanced removal. It indicates only that the allowable arithmetic mean calculated by the model may equal or exceed the regulatory concentration limit $X_{s}$when a comparatively high exceedance frequency is accepted. Accordingly, RC values generated on the increasing branch cannot be interpreted using the usual assumption that a larger coefficient necessarily represents lower variability.

The effect of the target probability was also examined using the configuration-level descriptive means for each parameter. The descriptive ordering was not constant across scenarios. For BOD, HCWs had the highest mean RCs from 50% to 70%, whereas HFCWs ranked highest from 80% onward. For COD, HCWs ranked highest at 50% and 60%, and VFCWs ranked highest from 70% onward. For TSS, VFCWs ranked highest from 50% to 70%, whereas HFCWs ranked highest from 80% onward. For TN, HCWs ranked highest at 50% and 60%, and VFCWs ranked highest from 70% onward. For TP, VFCWs ranked highest from 50% to 70%, whereas HFCWs ranked highest from 80% onward.These shifts reflect the distribution of the system- and parameter-specific CVs and the unequal composition of each group. They are descriptive features of the reviewed dataset and do not demonstrate an intrinsic configuration effect.

Figure S2: Mean Reliability Coefficient (RC) by constructed wetland configuration and target non-exceedance probability for (a) BOD, (b) COD, (c) TSS, (d) TN, and (e) TP.

Table S23: Mean Reliability Coefficient (RC) by parameter, constructed wetland configuration, and target non-exceedance probability.

| **Target non-exceedance probability** | **BOD** | | | **COD** | | | **TSS** | | | **TN** | | | **TP** | | |
| --- | --- | --- | --- | --- | --- | --- | --- | --- | --- | --- | --- | --- | --- | --- | --- |
|  | **VFCW (n=6)** | **HFCW (n=8)** | **HCW (n=8)** | **VFCW (n=12)** | **HFCW (n=10)** | **HCW (n=12)** | **VFCW (n=6)** | **HFCW (n=7)** | **HCW (n=9)** | **VFCW (n=10)** | **HFCW (n=5)** | **HCW (n=10)** | **VFCW (n=7)** | **HFCW (n=5)** | **HCW (n=6)** |
| 50% | 1.1232 | 1.1127 | 1.3997 | 1.1532 | 1.0950 | 1.1876 | 1.6681 | 1.1742 | 1.5972 | 1.0746 | 1.0638 | 1.1387 | 1.1799 | 1.0868 | 1.1246 |
| 60% | 1.0009 | 1.0052 | 1.1450 | 1.0220 | 0.9857 | 1.0313 | 1.3038 | 1.0306 | 1.2574 | 0.9952 | 0.9763 | 1.0059 | 1.0342 | 0.9899 | 1.0003 |
| 70% | 0.8865 | 0.9054 | 0.9292 | 0.9021 | 0.8814 | 0.8897 | 1.0140 | 0.9006 | 0.9808 | 0.9204 | 0.8912 | 0.8823 | 0.9030 | 0.8977 | 0.8839 |
| 80% | 0.7711 | 0.8053 | 0.7333 | 0.7842 | 0.7740 | 0.7514 | 0.7683 | 0.7738 | 0.7409 | 0.8439 | 0.8017 | 0.7584 | 0.7756 | 0.8031 | 0.7664 |
| 90% | 0.6383 | 0.6908 | 0.5349 | 0.6529 | 0.6474 | 0.5983 | 0.5392 | 0.6333 | 0.5113 | 0.7540 | 0.6932 | 0.6171 | 0.6350 | 0.6919 | 0.6314 |
| 95% | 0.5482 | 0.6132 | 0.4165 | 0.5665 | 0.5594 | 0.4982 | 0.4133 | 0.5411 | 0.3824 | 0.6912 | 0.6157 | 0.5220 | 0.5429 | 0.6146 | 0.5398 |
| 98% | 0.4637 | 0.5406 | 0.3175 | 0.4877 | 0.4753 | 0.4074 | 0.3147 | 0.4568 | 0.2803 | 0.6303 | 0.5395 | 0.4338 | 0.4588 | 0.5407 | 0.4541 |
| 99% | 0.4157 | 0.4992 | 0.2663 | 0.4438 | 0.4267 | 0.3572 | 0.2663 | 0.4098 | 0.2299 | 0.5945 | 0.4944 | 0.3840 | 0.4119 | 0.4979 | 0.4055 |
| 99.9% | 0.3089 | 0.4063 | 0.1661 | 0.3487 | 0.3166 | 0.2494 | 0.1757 | 0.3070 | 0.1367 | 0.5101 | 0.3885 | 0.2748 | 0.3092 | 0.4002 | 0.2979 |

Note: RC was recalculated for each system–parameter combination using the standard normal quantile corresponding to the target non-exceedance probability and was then averaged within each configuration. HCW values use the CV of the final treatment-stage effluent.

At the primary 95% target, the highest RCs were obtained for the HFCW evaluated by Ho et al. (2020) for BOD (RC = 0.964; CV = 0.023), the VFCW assessed by Suresh et al. (2023) for COD (RC = 0.925; CV = 0.048), the second HFCW evaluated by Ergaieg et al. (2021) for TSS (RC = 0.734; CV = 0.202), the VFCW evaluated by Li et al. (2023) for TN (RC = 0.901; CV = 0.065), and the HFCW evaluated by Mburu et al. (2013) for TP (RC = 0.853; CV = 0.100). These systems had the lowest CVs among the observations available for their respective parameters. Because all observed CVs were below the critical value at 95% and above, the complete system ordering was identical across the 95%, 98%, 99%, and 99.9% scenarios.

Using the 95% ordering as the reference, the ranking at 90% remained unchanged for BOD, COD, TN, and TP. The TSS ordering was nearly unchanged ($\rho=0.990$; $\tau_{b}=0.957$; five discordant pairs). At 80%, the highest-ranked system was retained for all five parameters, but the complete ordering changed for BOD ($\rho=0.895$; $\tau_{b}=0.853$; 17 discordant pairs), COD ($\rho=0.994$; $\tau_{b}=0.979$; six discordant pairs), TSS ($\rho=0.396$; $\tau_{b}=0.325$; 78 discordant pairs), and TP ($\rho=0.998$; $\tau_{b}=0.987$; one discordant pair). The TN ordering remained fully preserved ($\rho=1.000$; $\tau_{b}=1.000$). These results distinguish retention of the highest-ranked system from preservation of the complete ordering: the leading system was stable from 80% to 99.9%, whereas complete ranking stability for every parameter was observed only at 95% and above.

The ordering changed more markedly under the lower target probabilities. For BOD and COD, the highest RCs from 50% to 70% occurred in the HCW evaluated by Gajewska et al. (2018), with CVs of 2.009 and 1.485, respectively. For TSS and TP, the highest values over the same probability range occurred in the VFCWs evaluated by Santos et al. (2018) (CV = 2.435) and Fan et al. (2013) (CV = 1.333), respectively. For TN, the second HCW evaluated by Micek et al. (2020) (CV = 0.949) ranked first at 50% and 60%, whereas the VFCW evaluated by Li et al. (2023) ranked first from 70% onward. At 80%, the leading systems for all parameters were those with the lowest CVs. These reversals arose solely from the non-monotonic mathematical response of the RC equation below the critical probability-specific CV and do not indicate superior treatment performance or stability in the more variable systems.

No physical property of the CWs changed across these scenarios: the effluent means, SDs, and CVs remained fixed, and only the selected standard normal quantile changed. Lower target probabilities weakened the exponential penalty applied to variability and, above the critical CV, allowed the multiplicative $\sqrt{1+CV^{2}}$term to dominate. As the probability increased, the exponential penalty became dominant throughout the observed range, restoring the direct inverse association between CV and RC. Therefore, RC values should be compared only when calculated at the same target non-exceedance probability.

The 50%–70% scenarios were included to characterize model behavior and ranking sensitivity and should not be interpreted as recommended design or regulatory targets. Selection of a target probability should instead reflect the consequences of exceedance, receiving-water sensitivity, intended effluent use, uncertainty in the monitoring data, sampling frequency, and the technical capacity to control the treatment process (Alderson et al., 2015; Hamza et al., 2022; Lombard-Latune et al., 2020; Niku et al., 1979; Oliveira and von Sperling, 2008).

**Illustrative application of the RC-adjusted target to HFCW and VFCW design equations — Supplementary Method S2**

The mathematical effect can be illustrated using a BOD reference limit of 60 mg O₂ L⁻¹, an RC of 0.60, and a target non-exceedance probability of 95%. The allowable mean is:

$$M_{x}=0.60\times60=36\text{ }\text{mg O}_{2}\text{ }\text{L}^{-1}$$

For an influent design concentration of 200 mg O₂ L⁻¹, the logarithmic terms obtained by using the regulatory limit directly and by using the RC-adjusted mean are:

$$\ln\left( \frac{200}{60} \right)=1.204; \ln\left( \frac{200}{36} \right)=1.715$$

The ratio between the two design terms is:

$$\frac{1.715}{1.204}=1.424$$

For constant kinetic and hydraulic coefficients, the HFCW HRT and area would therefore be 42.4% higher when the RC-adjusted target is used. In the areal VFCW model, the allowable HLR would be 29.8% lower and the area 42.4% higher. These percentages express only the mathematical effect of replacing 60 mg O₂ L⁻¹ with 36 mg O₂ L⁻¹; they are not design recommendations and do not account for background concentration, temperature correction, loading limits, oxygen demand, or hydraulic constraints. The additional area is not imposed arbitrarily by the RC; it results from the lower mean concentration required to accommodate the assumed variability at the selected probability.

Table S24: Traceability of the constructed wetland systems included in the analytical dataset, showing the source, exact wetland configuration, information extracted, and DOI or full-text access link.

| **Dataset ID** | **Study** | **Exact CW type/configuration** | **Information extracted and used** | **DOI or access** |
| --- | --- | --- | --- | --- |
| VFCW-01 | Suresh et al. (2023) | Vertical-flow CW inserted between a septic tank/partially converted anaerobic filter and a soak pit. | System descriptors; influent and final-effluent means, dispersion (SD/CV), and removal efficiency for BOD, COD, TSS. | [doi:10.46488/NEPT.2023.v22i01.047](https://doi.org/10.46488/NEPT.2023.v22i01.047) |
| VFCW-02 | Chand et al. (2021) | Tidal-flow vertical CW with effluent recirculation; selected substrate + biochar + Typha unit (SBP). | System descriptors; influent and final-effluent means, dispersion (SD/CV), and removal efficiency for BOD, COD, TN. | [doi:10.1016/j.jwpe.2021.102292](https://doi.org/10.1016/j.jwpe.2021.102292) |
| VFCW-03 | Li et al. (2023) | Vertical-flow CW operated under a static magnetic field (MF-CW). | System descriptors; influent and final-effluent means, dispersion (SD/CV), and removal efficiency for COD, TN, TP. | [doi:10.1016/j.jenvman.2022.117192](https://doi.org/10.1016/j.jenvman.2022.117192) |
| VFCW-04 | Pillai and Nair (2021) | Planted subsurface vertical-flow CW mesocosm; Cumbu Napier hybrid grass unit. | System descriptors; influent and final-effluent means, dispersion (SD/CV), and removal efficiency for BOD, COD, TSS, TN. | [doi:10.46488/NEPT.2021.v20i02.010](https://doi.org/10.46488/NEPT.2021.v20i02.010) |
| VFCW-05 | Sun et al. (2021) | Modified vertical-flow CW combining biochar/activated-carbon media and denitrifying bacteria (VFCW-C). | System descriptors; influent and final-effluent means, dispersion (SD/CV), and removal efficiency for COD, TN, TP. | [doi:10.1016/j.jenvman.2020.111920](https://doi.org/10.1016/j.jenvman.2020.111920) |
| VFCW-06 | Wu et al. (2015) | Intermittently aerated vertical-flow CW treating synthetic domestic wastewater. | System descriptors; influent and final-effluent means, dispersion (SD/CV), and removal efficiency for COD, TN, TP. | [doi:10.1016/j.biortech.2014.11.041](https://doi.org/10.1016/j.biortech.2014.11.041) |
| VFCW-07 | Fan et al. (2013) | Intermittently aerated vertical-flow CW operated at controlled influent COD/N ratios. | System descriptors; influent and final-effluent means, dispersion (SD/CV), and removal efficiency for COD, TN, TP. | [doi:10.1016/j.biortech.2013.06.038](https://doi.org/10.1016/j.biortech.2013.06.038) |
| VFCW-08 | Chang et al. (2012) | Integrated vertical-flow CW with sequential downflow and upflow chambers; Canna indica + Pontederia cordata plot. | System descriptors; influent and final-effluent means, dispersion (SD/CV), and removal efficiency for COD, TN, TP. | [doi:10.1016/j.ecoleng.2012.03.019](https://doi.org/10.1016/j.ecoleng.2012.03.019) |
| VFCW-09 | Zurita et al. (2009) | Intermittently fed vertical subsurface-flow CW planted with ornamental species. | System descriptors; influent and final-effluent means, dispersion (SD/CV), and removal efficiency for BOD, COD, TSS, TN, TP. | [doi:10.1016/j.ecoleng.2008.12.026](https://doi.org/10.1016/j.ecoleng.2008.12.026) |
| VFCW-10 | Rodrigues et al. (2022) | Modified single-stage French-type vertical-flow CW using biochar and receiving raw domestic sewage. | System descriptors; influent and final-effluent means, dispersion (SD/CV), and removal efficiency for BOD, COD, TSS, TP. | [doi:10.36659/dae.2022.029](https://doi.org/10.36659/dae.2022.029) |
| VFCW-11 | Santos et al. (2018) | Partially saturated/flooded-bottom vertical-flow CW; lower 0.40 m maintained saturated. | System descriptors; influent and final-effluent means, dispersion (SD/CV), and removal efficiency for COD, TSS, TN. | [doi:10.17058/tecnolog.v22i1.10500](https://doi.org/10.17058/tecnolog.v22i1.10500) |
| VFCW-12 | Sezerino et al. (2023) | Partially saturated vertical-flow CW; 53% of filter depth saturated and evaluated over six years. | System descriptors; influent and final-effluent means, dispersion (SD/CV), and removal efficiency for BOD, COD, TSS, TN. | [doi:10.5327/276455760302003](https://doi.org/10.5327/276455760302003) |
| HFCW-01 | Chen et al. (2022) | Horizontal subsurface-flow CW experimental array; selected Iris sibirica unit aerated in the first quarter of the bed. | System descriptors; influent and final-effluent means, dispersion (SD/CV), and removal efficiency for COD, TN, TP. | [doi:10.3390/ijerph19031583](https://doi.org/10.3390/ijerph19031583) |
| HFCW-02 | Angassa et al. (2019) | Planted horizontal subsurface-flow CW with Phragmites karka; lower-HLR treatment condition. | System descriptors; influent and final-effluent means, dispersion (SD/CV), and removal efficiency for COD, TN, TP. | [doi:10.1007/s40710-019-00358-x](https://doi.org/10.1007/s40710-019-00358-x) |
| HFCW-03 | Wang and Li (2017) | Horizontal subsurface-flow CWs preceded by infiltration devices, with and without influent pre-aeration. | System descriptors; influent and final-effluent means, dispersion (SD/CV), and removal efficiency for BOD, COD, TN. | [doi:10.1007/s11356-016-7958-5](https://doi.org/10.1007/s11356-016-7958-5) |
| HFCW-04 | Mburu et al. (2013) | Pilot horizontal subsurface-flow CW integrated downstream of a primary facultative pond. | System descriptors; influent and final-effluent means, dispersion (SD/CV), and removal efficiency for BOD, COD, TSS, TP. | [doi:10.1016/j.jenvman.2013.05.031](https://doi.org/10.1016/j.jenvman.2013.05.031) |
| HFCW-05 | Zurita et al. (2009) | Continuously fed horizontal subsurface-flow CW planted with ornamental species. | System descriptors; influent and final-effluent means, dispersion (SD/CV), and removal efficiency for BOD, COD, TSS, TN, TP. | [doi:10.1016/j.ecoleng.2008.12.026](https://doi.org/10.1016/j.ecoleng.2008.12.026) |
| HFCW-06 | Ho et al. (2020) | Planted horizontal subsurface-flow CW with Brachiaria mutica. | System descriptors; influent and final-effluent means, dispersion (SD/CV), and removal efficiency for BOD, COD, TSS, TN, TP. | [doi:10.1007/s12649-020-01084-4](https://doi.org/10.1007/s12649-020-01084-4) |
| HFCW-07 | Ergaieg et al. (2021) | Full-scale horizontal subsurface-flow CW1 used for tertiary municipal-wastewater polishing. | System descriptors; influent and final-effluent means, dispersion (SD/CV), and removal efficiency for BOD, COD, TSS. | [doi:10.1007/s12517-021-08419-y](https://doi.org/10.1007/s12517-021-08419-y) |
| HFCW-08 | Ergaieg et al. (2021) | Full-scale horizontal subsurface-flow CW2 used for tertiary municipal-wastewater polishing. | System descriptors; influent and final-effluent means, dispersion (SD/CV), and removal efficiency for BOD, COD, TSS. | [doi:10.1007/s12517-021-08419-y](https://doi.org/10.1007/s12517-021-08419-y) |
| HFCW-09 | Sousa et al. (2024) | Full-scale horizontal-flow CW downstream of a septic tank at a rural inn. | System descriptors; influent and final-effluent means, dispersion (SD/CV), and removal efficiency for BOD, COD, TSS. | [doi:10.2166/washdev.2024.306](https://doi.org/10.2166/washdev.2024.306) |
| HFCW-10 | Silva et al. (2017) | Pilot horizontal subsurface-flow CW planted with Vetiver grass and operated after preliminary/primary treatment. | System descriptors; influent and final-effluent means, dispersion (SD/CV), and removal efficiency for BOD, COD, TSS. | [doi:10.5004/dwt.2017.21526](https://doi.org/10.5004/dwt.2017.21526) |
| HCW-01 | Martel-Rodríguez et al. (2022) | Hybrid system with two downflow vertical-flow CWs followed by one horizontal-flow CW (VF-HF). | System descriptors; influent and final-effluent means, dispersion (SD/CV), and removal efficiency for BOD, COD, TSS, TN. | [doi:10.3390/ijerph192214871](https://doi.org/10.3390/ijerph192214871) |
| HCW-02 | Zhai et al. (2016) | Hybrid vertical-baffled-flow wetland followed by a horizontal subsurface-flow wetland (VBFW-HSFW). | System descriptors; influent and final-effluent means, dispersion (SD/CV), and removal efficiency for COD, TN, TP. | [doi:10.1016/j.ecoleng.2016.10.045](https://doi.org/10.1016/j.ecoleng.2016.10.045) |
| HCW-03 | Marzec et al. (2024) | Aerated hybrid vertical-flow followed by horizontal-flow CW (VF-HF); wastewater aerated before the VF bed. | System descriptors; influent and final-effluent means, dispersion (SD/CV), and removal efficiency for BOD, COD, TSS. | [doi:10.3390/w16070947](https://doi.org/10.3390/w16070947) |
| HCW-04 | Ye and Li (2009) | Towery hybrid comprising HSSF-FWS cascade-HSSF stages; this is not a vertical-horizontal treatment train. | System descriptors; influent and final-effluent means, dispersion (SD/CV), and removal efficiency for COD, TSS, TN, TP. | [doi:10.1016/j.ecoleng.2009.03.009](https://doi.org/10.1016/j.ecoleng.2009.03.009) |
| HCW-05 | Micek et al. (2020) | Full-scale VF-HF hybrid, Roztocze National Park system 1 (Zwierzyniec). | System descriptors; influent and final-effluent means, dispersion (SD/CV), and removal efficiency for BOD, COD, TSS, TN, TP. | [doi:10.3390/w12123435](https://doi.org/10.3390/w12123435) |
| HCW-06 | Micek et al. (2020) | Full-scale VF-HF hybrid, Roztocze National Park system 2 (Florianka). | System descriptors; influent and final-effluent means, dispersion (SD/CV), and removal efficiency for BOD, COD, TSS, TN, TP. | [doi:10.3390/w12123435](https://doi.org/10.3390/w12123435) |
| HCW-07 | Marzec et al. (2019) | Full-scale VF-HF hybrid planted with giant miscanthus and Jerusalem artichoke. | System descriptors; influent and final-effluent means, dispersion (SD/CV), and removal efficiency for BOD, COD, TSS, TN, TP. | [doi:10.1016/j.ecoleng.2018.11.008](https://doi.org/10.1016/j.ecoleng.2018.11.008) |
| HCW-08 | Gajewska et al. (2018) | Full-scale horizontal-flow followed by vertical-flow hybrid (HF-VF; Dąbrowica I). | System descriptors; influent and final-effluent means, dispersion (SD/CV), and removal efficiency for BOD, COD, TN. | [doi:10.1016/j.ecoleng.2018.06.006](https://doi.org/10.1016/j.ecoleng.2018.06.006) |
| HCW-09 | Gajewska et al. (2018) | Full-scale vertical-flow followed by horizontal-flow hybrid (VF-HF; Dąbrowica II). | System descriptors; influent and final-effluent means, dispersion (SD/CV), and removal efficiency for BOD, COD, TN. | [doi:10.1016/j.ecoleng.2018.06.006](https://doi.org/10.1016/j.ecoleng.2018.06.006) |
| HCW-10 | Silva and Bueno (2015) | Pilot hybrid vertical-flow followed by horizontal-flow CW (VF-HF). | System descriptors; influent and final-effluent means, dispersion (SD/CV), and removal efficiency for COD, TSS, TP. | [doi:10.14295/holos.v15i2.9580](https://doi.org/10.14295/holos.v15i2.9580) |
| HCW-11 | Freitas (2017) | Full-scale descending vertical-flow CW followed by a saturated horizontal-flow CW (VF-HF); hybrid final effluent used. | System descriptors; influent and final-effluent means, dispersion (SD/CV), and removal efficiency for BOD, COD, TSS, TN. | [UFSC repository](https://repositorio.ufsc.br/handle/123456789/189315) |
| HCW-12 | Rousso (2017) | Full-scale descending vertical-flow CW followed by a saturated horizontal-flow CW (VF-HF); first two operating years. | System descriptors; influent and final-effluent means, dispersion (SD/CV), and removal efficiency for COD, TSS, TN. | [UFSC repository](https://repositorio.ufsc.br/handle/123456789/178979) |

**Operational interpretation and complementary checks**

Common CW limitations may produce different combinations of mean deterioration and increased variability. The RC can identify when variability changes, and $R_{m}$can identify when the observed mean no longer satisfies the allowable target, but neither metric directly diagnoses seasonality, start-up instability, clogging, substrate exhaustion, or nutrient-process limitations. The complementary checks and possible responses are summarized in Table S25.

Table S25: RC-based signals, diagnostic limitations, and complementary operational or maintenance responses for common constructed-wetland limitations.

| **Limitation or process** | **RC-based signal and diagnostic limitation** | **Complementary checks and potential response** |
| --- | --- | --- |
| Climate and seasonality | Seasonal decreases in RC or increases in Rₘ may reflect temperature-dependent kinetics, evapotranspiration, rainfall, or seasonal loading. The indicators do not separate these effects. | Calculate seasonal RC values; monitor temperature, flow, loading, water level, and DO. Adjust loading, aeration, recirculation, or saturated depth where operationally feasible. |
| Start-up and maturation | Low or unstable RC during start-up may reflect incomplete biofilm establishment, vegetation development, or hydraulic stabilization. Early data may not represent mature operation. | Separate commissioning from stable-operation data; monitor biofilm establishment, vegetation, DO, and effluent trends. Apply staged loading and extend acclimation before defining the operational baseline. |
| Solids accumulation and clogging | A decreasing TSS RC or increasing Rₘ may accompany solids breakthrough, preferential flow, drainage restriction, or clogging, but RC alone cannot confirm hydraulic failure. | Measure headloss and hydraulic conductivity; inspect inlet distribution, drainage, and solids accumulation. Restore pretreatment, clean distribution devices, remove deposits, or rehabilitate affected media. |
| BOD and COD instability | A decreasing RC or increasing Rₘ may be associated with variable organic loading, impaired pretreatment, uneven distribution, reduced effective HRT, or insufficient oxygen transfer. | Check influent load, pretreatment, dosing, water level, distribution, DO, and HRT. Adjust loading or dosing, restore pretreatment, improve distribution, or evaluate recirculation and aeration. |
| TN removal | Changes in RC or Rₘ may reflect unstable coupling of nitrification and denitrification. The indicators do not identify whether oxygen, carbon, redox conditions, HRT, temperature, or recirculation is limiting. | Measure NH₄⁺-N, NO₂⁻-N, NO₃⁻-N, DO, redox potential, temperature, COD/N, and HRT. Adjust dosing/resting cycles, saturated depth, recirculation, or carbon availability; add a complementary stage when required. |
| TP removal | A persistent increase in Rₘ, with or without declining RC, may indicate loss of P-retention margin. Media saturation cannot be inferred from RC alone. | Assess cumulative P loading, media age and mineralogy, pH, Ca/Fe/Al availability, hydraulic contact, and biomass removal. Replace or supplement reactive media or add a polishing step when retention capacity is exhausted. |

Note: Abbreviations: RC, reliability coefficient; Rₘ, observed-to-allowable mean concentration ratio; BOD, biochemical oxygen demand; COD, chemical oxygen demand; TSS, total suspended solids; TN, total nitrogen; TP, total phosphorus; HRT, hydraulic retention time; DO, dissolved oxygen; COD/N, chemical oxygen demand-to-nitrogen ratio; NH₄⁺–N, ammonium nitrogen; NO₂⁻–N, nitrite nitrogen; NO₃⁻–N, nitrate nitrogen.

**Upper-performing CW systems and associated design and operational features**

Supplementary Table S26 presents the representative upper-performing systems identified within the reviewed dataset and the design and operational features associated with their reported removal efficiencies. These cases represent the upper range of the analyzed systems rather than universal maxima, an exhaustive ranking, or configuration-specific benchmarks. The detailed interpretations below support the process-based synthesis presented in Section 3.2.1 of the main manuscript.

Table S26: Representative upper-performing constructed wetland systems and the main design or operational features associated with their reported removal efficiencies.

| **CW type** | **Parameter(s)** | **Study** | **Removal (%)** | **Reported design and operational features** |
| --- | --- | --- | --- | --- |
| VFCW | BOD | Chand et al. (2021) | 96.1 | Tidal-flow mesocosm with effluent recirculation; sand, gravel, and biochar; Typha sp.; 216-h experimental run. |
| VFCW | COD | Wu et al. (2015) | 98.0 | Intermittent aeration; Phragmites australis; 72-h HRT; synthetic wastewater; aerated and non-aerated microcosms. |
| VFCW | TN | Wu et al. (2015) | 89.0 | Intermittent aeration; Phragmites australis; 72-h HRT; synthetic wastewater; aerated and non-aerated microcosms operated at different influent strengths. |
| VFCW | TN | Fan et al. (2013) | 89.0 | Intermittent aeration; controlled influent COD/N ratio; synthetic wastewater; alternating aerated and non-aerated periods. |
| VFCW | TSS | Sezerino et al. (2023) | 99.0 | Septic-tank pretreatment; intermittently fed sand–gravel bed; partially saturated zone; alternating 3.5-day feeding and resting periods; six-year operation. |
| VFCW | TP | Li et al. (2023) | 96.0 | Gravel–sand media; intermittent aeration; static magnetic-field treatment; mineral-media retention remained a plausible dominant pathway. |
| HFCW | BOD | Ho et al. (2020) | 93.3 | Sand–gravel bed planted with Brachiaria mutica; controlled subsurface water level; performance evaluated under increasing organic loads. |
| HFCW | COD; TN | Angassa et al. (2019) | 94.1; 97.3 | Gravel bed planted with Phragmites karka; HLR of 0.025 m d⁻¹; HRT of 6 d; planted and unplanted controls. |
| HFCW | TSS | Zurita et al. (2009) | 84.7 | Continuous feeding; HRT of 4 d; porous tezontle medium; mixed ornamental vegetation. |
| HFCW | TP | Chen et al. (2022) | 96.0 | Ceramsite and zeolite media; plant and aeration treatments; TP removal was not significantly affected by aeration position or plant species. |
| HCW | BOD; COD | Marzec et al. (2019) | 98.8; 97.6 | Primary settling; intermittently fed sand-based VF bed followed by HF polishing; operating full-scale on-site VF–HF treatment plant serving a multi-family building and monitored for five years. |
| HCW | COD; TSS | Rousso (2017) | 98.0; 99.9 | Septic-tank pretreatment; intermittently fed VFCW followed by saturated HFCW; sand and gravel; full-scale household system. |
| HCW | TSS | Silva and Bueno (2015) | 99.6 | Preliminary treatment; stratified crushed-stone VF bed followed by a saturated HF bed; intermittent VF feeding and sequential filtration. |
| HCW | TN; TP | Micek et al. (2020) | 86.0; 94.0 | Full-scale sand-based VF–HF system; ammonium oxidation in the VF bed; longer contact and nitrate reduction in the HF bed. |

Note: Abbreviations: VF, vertical-flow stage; HF, horizontal-flow stage; COD/N, chemical oxygen demand-to-nitrogen ratio.

The upper-performing VFCWs combined oxygen renewal with hydraulic cycling, reactive media, or controlled carbon supply. Chand et al. (2021) reported 96.1% BOD removal in a tidal-flow mesocosm containing biochar and Typha sp. and operated with effluent recirculation. The observed order among the tested units (biochar + plants > plants alone > biochar alone) supports a combined contribution of the plant–media matrix under the evaluated conditions, although the 216-h experimental duration does not establish long-term stability. Wu et al. (2015) obtained 98% COD and 89% TN removal in intermittently aerated VFCWs treating synthetic wastewater. Aerated units outperformed non-aerated controls across influent strengths, consistent with enhanced organic oxidation and nitrification while non-aerated intervals supported denitrification. Fan et al. (2013) also reached 89% TN removal at the precision reported in the compiled dataset, showing the importance of coordinating intermittent aeration with an adequate influent COD/N ratio. The 99% TSS removal reported by Sezerino et al. (2023) occurred in a long-term, intermittently fed sand–gravel bed with a partially saturated zone, consistent with filtration and particle retention during successive feeding and resting periods. The 96% TP removal reported by Li et al. (2023) occurred in a mineral-media VFCW operated with intermittent aeration and a static magnetic field; however, the available comparison does not establish the magnetic treatment as the dominant phosphorus-removal pathway, and retention by the media remains the more defensible interpretation.

The upper-performing HFCWs reflected combinations of hydraulic residence, vegetation, and filter media. Ho et al. (2020) reported 93.3% BOD removal in a sand–gravel HFCW planted with Brachiaria mutica. Removal remained high at the largest organic loading evaluated, indicating that the unit sustained organic-matter treatment as HRT decreased; however, the independent contribution of vegetation could not be isolated for all parameters. In the planted gravel bed evaluated by Angassa et al. (2019), COD and TN removals of 94.1 and 97.3%, respectively, occurred at the lower HLR of 0.025 m d⁻¹ and an HRT of 6 d. Removal was higher in the planted than in the unplanted unit, supporting a contribution from Phragmites karka and its root-associated biofilm together with the longer contact time. Zurita et al. (2009) obtained 84.7% TSS removal in a continuously fed HFCW operated at an HRT of 4 d. The result was consistent with sedimentation and filtration in the porous tezontle bed and with the root–media matrix of the mixed ornamental vegetation, although these effects cannot be separated from the hydraulic conditions. In Chen et al. (2022), the highest HFCW TP removal occurred in ceramsite- and zeolite-based beds. Aeration position, plant species, and their interaction did not significantly affect TP removal, indicating that media-associated retention, rather than oxygen supply or vegetation, governed the observed result.

Among HCWs, Marzec et al. (2019) reported mean removals of 98.8% for BOD and 97.6% for COD in an operating full-scale VF–HF treatment plant serving a multi-family building and monitored over five years. Mean influent BOD and COD concentrations were 537 and 1309 mg O₂ L⁻¹, respectively. Most removal occurred in the sand-based VF bed, which was fed twice daily at an HLR of 12.5 mm d⁻¹ and an estimated HRT of 4.8 d. Dissolved oxygen increased from 0.50 mg L⁻¹ at the VF inlet to 2.92 mg L⁻¹ at its outlet, supporting the interpretation that intermittent feeding and partial drainage favored atmospheric oxygen transfer. The downstream HF bed, operated at an estimated HRT of 10.6–21.2 d, further reduced BOD and COD to 6.6 and 31.8 mg O₂ L⁻¹. Rousso (2017) also reported 98.0% COD and approximately 99.9% TSS removal in a full-scale household VF–HF system, with final concentrations of 12 mg O₂ L⁻¹ and 0.04 mg L⁻¹, respectively. The approximately 99.6% TSS removal calculated for the pilot VF–HF system of Silva and Bueno (2015) likewise reflects sequential filtration and polishing, but the result should be interpreted cautiously because the influent TSS concentration was exceptionally high and variable.

The nutrient results show that high removal required process complementarity rather than configuration alone. In system 2 at Florianka, the better-performing of the two full-scale VF–HF systems evaluated by Micek et al. (2020), TN and TP removals reached 86 and 94%, respectively. In the VF beds, ammonium oxidation was accompanied by increases in nitrate, nitrite, and dissolved oxygen, indicating effective nitrification. The downstream HF beds provided additional ammonium and nitrate removal; in system 2, the HF stage maintained substantially lower residual ammonium, nitrate, and nitrite concentrations than in system 1. The contrast between the two systems showed that the VF–HF sequence alone did not determine nitrogen performance. The source study related the weaker nitrogen performance of system 1 to insufficient biodegradable carbon for denitrification, whereas system 2 provided more favorable conditions for both nitrification and denitrification. For TP, the HF bed accounted for most of the stage-specific removal, and the source study associated its stronger performance mainly with the longer hydraulic retention time. The individual contributions of the sand medium and vegetation were not isolated experimentally. Because phosphorus retention may decline as the sorption capacity of the filter material is progressively exhausted, high mean removal should not be interpreted as permanent long-term retention.

**References**

M.P. Alderson, A.B. dos Santos, C.R. Mota Filho, Reliability analysis of low-cost, full-scale domestic wastewater treatment plants for reuse in aquaculture and agriculture, Ecol. Eng. 82 (2015) 6–14. <https://doi.org/10.1016/j.ecoleng.2015.04.081>.

K. Angassa, S. Leta, W. Mulat, H. Kloos, E. Meers, Evaluation of pilot-scale constructed wetlands with Phragmites karka for phytoremediation of municipal wastewater and biomass production in Ethiopia, Environ. Process. 6 (2019) 65–84. https://doi.org/10.1007/s40710-019-00358-x.

N. Chand, S. Suthar, K. Kumar, Wastewater nutrients and coliforms removals in tidal flow constructed wetland: effect of the plant (Typha) stand and biochar addition, J. Water Process Eng. 43 (2021) 102292. <https://doi.org/10.1016/j.jwpe.2021.102292>.

J.-J. Chang, S.-Q. Wu, Y.-R. Dai, W. Liang, Z.-B. Wu, Treatment performance of integrated vertical-flow constructed wetland plots for domestic wastewater, Ecol. Eng. 44 (2012) 152–159. https://doi.org/10.1016/j.ecoleng.2012.03.019.

X. Chen, F. Zhong, Y. Chen, J. Wu, S. Cheng, The interaction effects of aeration and plant on the purification performance of horizontal subsurface flow constructed wetland, Int. J. Environ. Res. Public Health 19(3) (2022) 1583. https://doi.org/10.3390/ijerph19031583.

K. Ergaieg, M.H. Msaddek, A. Kallel, I. Trabelsi, Monitoring of horizontal subsurface flow constructed wetlands for tertiary treatment of municipal wastewater, Arab. J. Geosci. 14 (2021) 2045. <https://doi.org/10.1007/s12517-021-08419-y>.

J. Fan, W. Wang, B. Zhang, Y. Guo, H.H. Ngo, W. Guo, J. Zhang, H. Wu, Nitrogen removal in intermittently aerated vertical flow constructed wetlands: impact of influent COD/N ratios, Bioresour. Technol. 143 (2013) 461–466. <https://doi.org/10.1016/j.biortech.2013.06.038>.

M.N. de Freitas, Avaliação da hidrodinâmica e do desempenho de um wetland construído horizontal componente de um sistema híbrido empregado no tratamento de esgoto sanitário, Master’s dissertation, Universidade Federal de Santa Catarina, Florianópolis, 2017. Available at: https://repositorio.ufsc.br/handle/123456789/189315.

M. Gajewska, K. Skrzypiec, K. Jóźwiakowski, P. Bugajski, Kinetics of pollutants removal in hybrid treatment wetlands—Case study comparison, Ecol. Eng. 120 (2018) 222–229. <https://doi.org/10.1016/j.ecoleng.2018.06.006>.

R. Hamza, M.F. Hamoda, M. Elassar, Energy and reliability analysis of wastewater treatment plants in small communities in Ontario, Water Sci. Technol. 85 (2022) 1824–1839. <https://doi.org/10.2166/wst.2022.093>.

V.T.T. Ho, M.P. Dang, L.T. Lien, T.T. Huynh, T.V. Hung, L.G. Bach, Study on domestic wastewater treatment of the horizontal subsurface flow wetlands (HSSF-CWs) using Brachiaria mutica, Waste Biomass Valor. 11 (2020) 5627–5634. <https://doi.org/10.1007/s12649-020-01084-4>.

C. Li, L. Feng, J. Lian, X. Yu, C. Fan, Z. Hu, H. Wu, Enhancement of organics and nutrient removal and microbial mechanism in vertical flow constructed wetland under a static magnetic field, J. Environ. Manage. 330 (2023) 117192. <https://doi.org/10.1016/j.jenvman.2022.117192>.

R. Lombard-Latune, F. Leriquier, C. Oucacha, L. Pelus, G. Lacombe, B. Le Guennec, P. Molle, Performance and reliability comparison of French vertical flow treatment wetlands with other decentralized wastewater treatment technologies in tropical climates, Water Sci. Technol. 82 (2020) 1701–1709. <https://doi.org/10.2166/wst.2020.444>.

G.M. Martel-Rodríguez, V. Millán-Gabet, C.A. Mendieta-Pino, E. García-Romero, J.R. Sánchez-Ramírez, Long-term performance of a hybrid-flow constructed wetlands system for urban wastewater treatment in Caldera de Tirajana (Santa Lucía, Gran Canaria, Spain), Int. J. Environ. Res. Public Health 19(22) (2022) 14871. https://doi.org/10.3390/ijerph192214871.

M. Marzec, M. Gizińska-Górna, K. Jóźwiakowski, A. Pytka-Woszczyło, A. Kowalczyk-Juśko, M. Gajewska, The efficiency and reliability of pollutant removal in a hybrid constructed wetland with giant miscanthus and Jerusalem artichoke in Poland, Ecol. Eng. 127 (2019) 23–35. https://doi.org/10.1016/j.ecoleng.2018.11.008.

M. Marzec, A. Listosz, A. Malik, M. Kulik, K. Jóźwiakowski, Organic pollutants removal in a hybrid constructed wetland wastewater treatment plant with an aeration system, Water 16(7) (2024) 947. https://doi.org/10.3390/w16070947.

N. Mburu, S.M. Tebitendwa, J.J.A. van Bruggen, D.P.L. Rousseau, P.N.L. Lens, Performance comparison and economics analysis of waste stabilization ponds and horizontal subsurface flow constructed wetlands treating domestic wastewater: a case study of the Juja sewage treatment works, J. Environ. Manage. 128 (2013) 220–225. <https://doi.org/10.1016/j.jenvman.2013.05.031>.

A. Micek, K. Jóźwiakowski, M. Marzec, A. Listosz, Technological reliability and efficiency of wastewater treatment in two hybrid constructed wetlands in the Roztocze National Park (Poland), Water 12 (2020) 3435. <https://doi.org/10.3390/w12123435>.

S. Niku, E.D. Schroeder, F.J. Samaniego, Performance of activated sludge processes and reliability-based design, J. Water Pollut. Control Fed. 51(12) (1979) 2841–2857.

S.C. Oliveira, M. von Sperling, Reliability analysis of wastewater treatment plants, Water Res. 42(4–5) (2008) 1182–1194. <https://doi.org/10.1016/j.watres.2007.09.001>.

J.S. Pillai, A.N.B. Nair, Performance of vertical flow constructed wetlands planted with indigenous species for decentralized wastewater treatment and biomass production in Kerala, India, Nat. Environ. Pollut. Technol. 20(2) (2021) 541–550. https://doi.org/10.46488/NEPT.2021.v20i02.010.

E.B. Rodrigues, P.H. Sezerino, D.D. Silveira, E. Skoronski, F.R. Lapolli, Utilização do biocarvão como meio filtrante de wetland construído vertical empregado no tratamento de esgoto bruto, Revista DAE 70(235) (2022) 180–192. https://doi.org/10.36659/dae.2022.029.

B.Z. Rousso, Avaliação de um sistema híbrido de wetlands construídos empregado no tratamento de esgoto sanitário, Master’s dissertation, Universidade Federal de Santa Catarina, Florianópolis, 2017. Available at: https://repositorio.ufsc.br/handle/123456789/178979.

M.O. dos Santos, C. Pelissari, B.Z. Rousso, M.N. de Freitas, P.H. Sezerino, Avaliação dos dois primeiros anos de operação de um wetland construído vertical de fundo saturado aplicado no tratamento de esgoto sanitário, Tecno-Lógica 22 (2018) 25–29. <https://doi.org/10.17058/tecnolog.v22i1.10500>.

P.H. Sezerino, B.B.B. Silva, A.R. da Silva, L. Bassani, C. Pelissari, Partially saturated vertical flow constructed wetland for urban wastewater treatment, Cad. Téc. Eng. Sanit. Ambient. 3(2) (2023) 23–30. <https://doi.org/10.5327/276455760302003>.

J.A. da Silva, A. Sarti, G.H.R. da Silva, Performances of two pilot decentralized wastewater treatment plants used to treat low-strength wastewater, Desalin. Water Treat. 91 (2017) 93–100. https://doi.org/10.5004/dwt.2017.21526.

M.V. Silva, R.F. Bueno, Avaliação de um wetland construído híbrido no tratamento de esgoto sanitário, Holos Environ. 15(2) (2015) 211–222. https://doi.org/10.14295/holos.v15i2.9580.

A.R. Sousa, C. Pelissari, A.T. Laureano, P.H. Sezerino, Effluent quality and reuse potential of wastewater treated in constructed wetlands in Southern Brazil, J. Water Sanit. Hyg. Dev. 14(5) (2024) 377–388. https://doi.org/10.2166/washdev.2024.306.

Y. Sun, P. Zhou, N. Zhang, Z. Zhang, Q. Guo, C. Chen, L. Cui, Effects of matrix modification and bacteria amendment on the treatment efficiency of municipal tailwater pollutants by modified vertical flow constructed wetland, J. Environ. Manage. 281 (2021) 111920. https://doi.org/10.1016/j.jenvman.2020.111920.

S. Suresh, P. Sharma, R.R. Yaragal, S. Mutnuri, Study on effectiveness of intervention of a vertical flow constructed wetland in between septic tank and soak pit for the treatment of septic tank effluent, Nat. Environ. Pollut. Technol. 22 (2023) 489–496. <https://doi.org/10.46488/NEPT.2023.v22i01.047>.

L. Wang, T. Li, Seasonal effects of pre-aeration on microbial processes for nitrogen removal in constructed wetlands, Environ. Sci. Pollut. Res. 24 (2017) 3810–3819. https://doi.org/10.1007/s11356-016-7958-5.

H. Wu, J. Fan, J. Zhang, H.H. Ngo, W. Guo, Z. Hu, S. Liang, Decentralized domestic wastewater treatment using intermittently aerated vertical flow constructed wetlands: impact of influent strengths, Bioresour. Technol. 176 (2015) 163–168. <https://doi.org/10.1016/j.biortech.2014.11.041>.

F. Ye, Y. Li, Enhancement of nitrogen removal in towery hybrid constructed wetland to treat domestic wastewater for small rural communities, Ecol. Eng. 35(7) (2009) 1043–1050. https://doi.org/10.1016/j.ecoleng.2009.03.009.

J. Zhai, M.H. Rahaman, X. Chen, H. Xiao, K. Liao, X. Li, C. Duan, B. Zhang, G. Tao, Y. John, J. Vymazal, New nitrogen removal pathways in a full-scale hybrid constructed wetland proposed from high-throughput sequencing and isotopic tracing results, Ecol. Eng. 97 (2016) 434–443. https://doi.org/10.1016/j.ecoleng.2016.10.045.

F. Zurita, J. de Anda, M.A. Belmont, Treatment of domestic wastewater and production of commercial flowers in vertical and horizontal subsurface-flow constructed wetlands, Ecol. Eng. 35 (2009) 861–869. <https://doi.org/10.1016/j.ecoleng.2008.12.026>.
